# Physiological measurement of emotion from infancy to preschool: A systematic review and meta‐analysis

**DOI:** 10.1002/brb3.1989

**Published:** 2020-12-17

**Authors:** Lori‐Ann R. Sacrey, Sarah Raza, Vickie Armstrong, Jessica A. Brian, Azadeh Kushki, Isabel M. Smith, Lonnie Zwaigenbaum

**Affiliations:** ^1^ University of Alberta/Autism Research Centre Glenrose Rehabilitation Hospital Edmonton AB Canada; ^2^ Dalhousie University/Autism Research Centre IWK Health Centre Halifax NS Canada; ^3^ University of Toronto/Autism Research Centre Bloorview Research Institute Toronto ON Canada

**Keywords:** behavioral regulation, early development, emotion, emotion regulation, heart rate, meta‐analysis, meta‐regression, physiology, RSA

## Abstract

**Introduction:**

Emotion regulation, the ability to regulate emotional responses to environmental stimuli, develops in the first years of life and plays an important role in the development of personality, social competence, and behavior. Substantial literature suggests a relationship between emotion regulation and cardiac physiology; specifically, heart rate changes in response to positive or negative emotion‐eliciting stimuli.

**Method:**

This systematic review and meta‐analysis provide an in‐depth examination of research that has measured physiological responding during emotional‐evoking tasks in children from birth to 4 years of age.

**Results:**

The review had three main findings. First, meta‐regressions resulted in an age‐related decrease in baseline and task‐related heart rate (HR) and increases in baseline and task‐related respiratory sinus arrhythmia (RSA). Second, meta‐analyses suggest task‐related increases in HR and decreases in RSA and heart rate variability (HRV), regardless of emotional valence of the task. Third, associations between physiological responding and observed behavioral regulation are not consistently present in children aged 4 and younger. The review also provides a summary of the various methodology used to measure physiological reactions to emotional‐evoking tasks, including number of sensors used and placement, various baseline and emotional‐evoking tasks used, methods for extracting RSA, as well as percentage of loss and reasons for loss for each study.

**Conclusion:**

Characterizing the physiological reactivity of typically developing children is important to understanding the role emotional regulation plays in typical and atypical development.

## Graphical abstract

This review examines the physiological responses to emotion‐evoking tasks in children from birth to age 4. We reviewed 64 articles, allowing a thorough and in‐depth exploration of different baseline and emotion tasks across the preschool age. This review provides an overview of results as well as meta‐analyses to explore the strength of the findings.

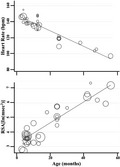

## 1 INTRODUCTION

Emotion regulation, the ability to regulate emotional reactions to environmental cues, develops during the first years of life (Calkins, 1994; Eisenberg et al., 1995, Eisenberg et al., 1996; Kopp, 1982; Thompson, 1994). The behavioral and cognitive constructs of emotion regulation have been extensively studied in the developmental psychology literature and suggest that personality, social competence, and problematic behavior have their origins in (or are influenced by) early emotional regulation (Calkins, 1994; Calkins & Keane, 2004; Cicchetti et al., 1991; Cole et al., 1994; Stifter et al., 1999). Yet, many behaviors of interest may be difficult to assess in young children who do not have the ability to communicate verbally. As such, physiological measurement is necessary to better understand age‐related changes and individual differences in response to environmental challenges (Campos, 1976; Davidson, 2001; Lacey et al., 1963). One such index of physiological arousal is heart rate. Measuring heart rate variability, the increase or decrease in time intervals between successive heart beats, has become an important component of psychophysiological research with the introduction of more accessible child and adult equipment and methodological guidelines (Berntson et al., 1997; Mailk, 1996).

Autonomic nervous system reactivity is assessed as the difference between resting (baseline) heart rate (or a calculated metric, such as heart rate variability) and heart rate during a physical or emotional challenge (e.g., still‐face paradigm; Critchley et al., 2005; Jones‐Mason et al., 2018). Early clinical uses of heart rate variability included identification of fetal distress (Hon, 1958; Lee & Hon, 1958) and contributions of the central nervous system to sudden cardiac death (Wolf, 1967). Lacey and Lacey (1958) were the first to describe how measurement of heart rate was sensitive to changes in one's environment, with deceleration associated with acceptance and acceleration associated with rejection of the environment (Lacey et al., 1963). From these early insights, we garnered an understanding that the autonomic nervous system (ANS) maintains homeostasis by facilitating responding to our internal and external environment (e.g., surprise; Bernston, Cacioppo, & Quigley, 1993; Mendes, 2009; Calkins & Marcovitch, 2010; Porges, 1985, 1992, 2007, 2011). These modifications are brought about by the sensory and motor neurons of the ANS, which connect the central nervous system to the internal organs and the endocrine system. The ANS itself is comprised of two systems, the sympathetic nervous system, which is responsible for our *flight or fight response* (to mobilize energy, accelerate heart rate, and slow digestion), and the parasympathetic nervous system, which is responsible for our *rest and digest response* (decelerate heart rate and increase blood flow to gastrointestinal organs to support digestion; Alkon et al., 2014; Sapolsky, 2004; Selye, 1956).

**Figure 1:**
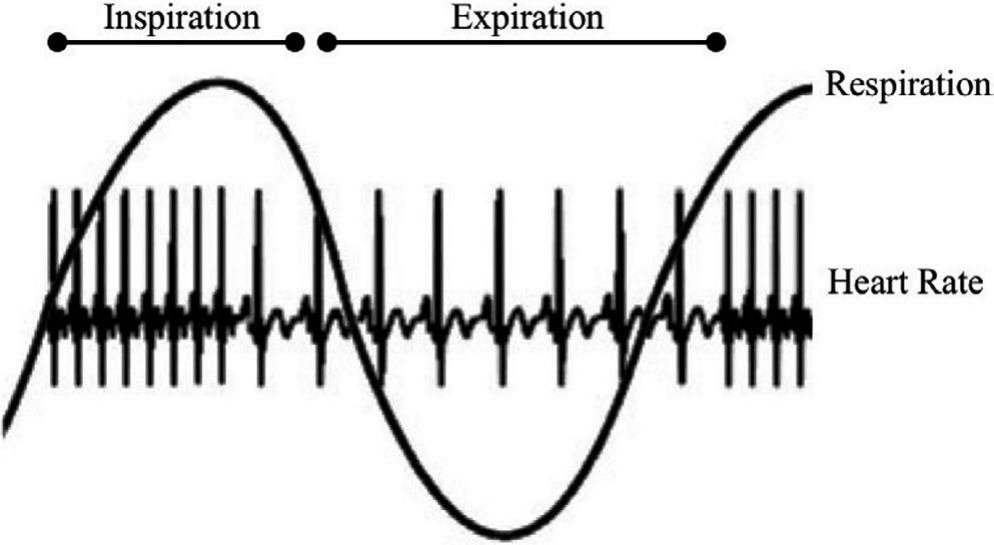
An oversimplified demonstration of the effect of respiration on heart rate. Heart rate accelerates during inspiration and decelerates during exhalation

The tenth cranial nerve, or vagus, is believed to be responsible for maintaining homeostasis via bidirectional messages between the internal organs (including the heart and lungs) and the brain (Porges, 2011; Cacioppo & Bernston, 2011). As such, *vagal tone*, a measure of parasympathetic activity, is often used as an indicator of self‐regulation (Porges, 1985, 2007). Because vagal tone cannot be measured directly, various indirect indices are used. Respiratory sinus arrhythmia (RSA), as illustrated in Figure 1, is a measure of changes in heart rate due to respiration (heart rate increases during inhalation and decreases during exhalation; Zisner & Beauchaine, 2016). Decreased RSA represents parasympathetic nervous system withdrawal (resulting in heart rate increase), and increased RSA represents parasympathetic nervous system activation (resulting in heart rate decrease; Moore & Calkins, 2004). In healthy children and adults, heart rate variability (HRV) tends to occur within the respiratory frequency of 0.15–0.4 Hz at rest (i.e., high‐frequency HRV; Wallis et al., 2005), although it can extend to frequencies between 0.15 Hz and up to 1.0 Hz for infants or adults when physically active (Bernston et al., 1997). To account for respiration changes across development, research that examines HRV or RSA in very young children often employ the respiration bandwidth filter of 0.24–1.04 Hz, which approximates 15–60 breaths per minute (Porges, 1985, 1985b; Shader et al., 2018).

A large body of work has suggested that HRV may serve as a physiological marker of emotion regulation in children (Beauchaine, 2015; Fox, 1989; Fox et al., 2000; Harper et al., 1977; Propper & Moore, 2006). A review of physiological measurement in healthy individuals has suggested that heart rate (HR) shows distinctive patterns of responding to different emotions (Kreibig, 2010). That is, HR increases during tasks designed to elicit negative (including anger, anxiety, fear, and sadness) and positive (including happiness, joy, and surprise) emotions, but decreases during tasks associated with passivity, including noncrying sadness, affection, contentment, and visual anticipatory pleasure. The studies reviewed by in Kreibig (2010) included “healthy individuals,” but there were no limits surrounding age. Because research shows broad individual differences in how young children respond to emotion‐evoking stimuli (Aureli et al., 2015; Buss et al., 2005; Calkins et al., 2007; Dale et al., 2011; Lewis et al., 2004; Quas et al., 2000), an examination of physiological reactivity in children aged 4 years and under is warranted. The purpose of this review is to provide an in‐depth examination of research that has measured physiological responses during emotion‐evoking tasks in children from birth to children age 4 years or younger. Specifically, we aim to (a) describe patterns of ANS activity across different baseline tasks, (b) describe patterns of ANS activity across different emotion‐evoking tasks, (c) describe relationships between behavioral and physiological responses (where available), and (*d*) conduct meta‐analyses to evaluate the presence of predictable patterns of reactions to emotion‐evoking tasks in children age 4 years or younger.

## 2 METHOD

### 2.1 Search strategy

A systematic literature review was completed in accordance with the Preferred Reporting Items for Systematic Reviews and Meta‐analyses (PRISMA; Moher et al., 2009) checklist. Searches were performed between 7 and 9 March 2019 in four databases: PsycINFO (date range: 1806–March Week 1 2019), Web of Science (date range: all years to 8 March 2019), CINAHL Plus (date range: all years to 8 March 2019), and Ovid MEDLINE(R) (date range: 1946–7 March 2019). Search terms and strategy were refined in collaboration with a University of Alberta health sciences librarian and included combinations of search terms for emotion, physiology, and child. Our complete search strategies can be found in Appendix S1. The search results were imported into Covidence (covidence.org) for review, resulting in 2,598 articles following duplicate removal. Using the same search terms and databases, a second search was completed covering the dates between 7 March 2019 to 11 February 2020 to identify any additional articles that met inclusion criteria to ensure the systematic review and meta‐analysis was updated prior to publication. Following duplicate removal, 121 additional articles were identified for potential inclusion.

### 2.2 Screening for inclusion and exclusion criteria

To be included in the review, a paper had to (1) use an emotion‐evoking task; (2) measure heart rate during baseline and emotion‐evoking tasks; and (3) include a sample of typically developing children aged four years or less. A paper was excluded if (1) physiological measures were collected during exercise, surgery, medical treatment, sleep, or intervention; or if it (2) was a case study/case series or (3) was a review article, commentary, or conference abstract; or (4) did not include a sample of typically developing children.

**Figure 2:**
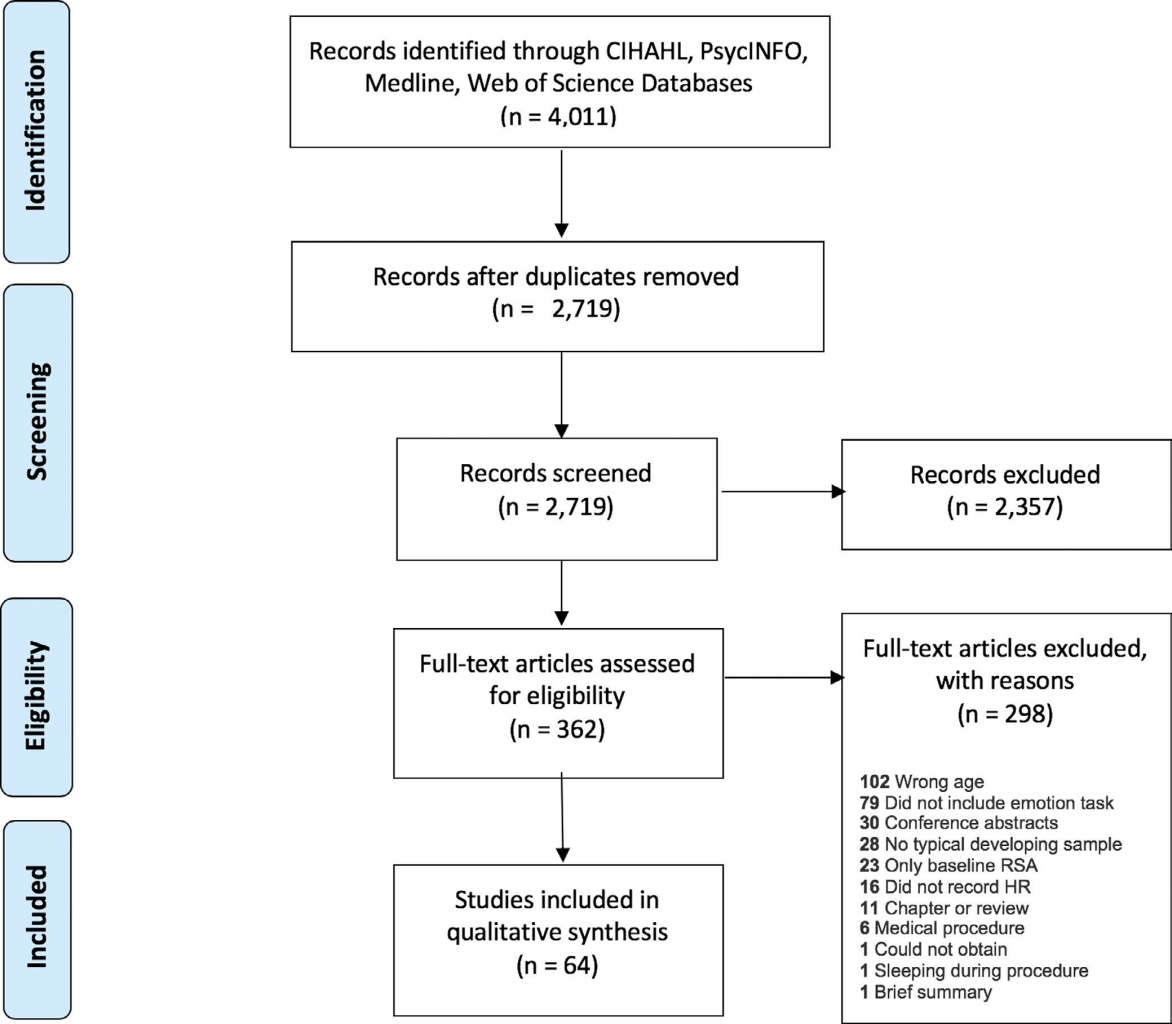
Systematic review strategy using the PRISMA method (Moher et al., 2009)

Titles and abstracts of 2,719 articles were independently screened using the inclusion and exclusion criteria in Covidence by two authors (LRS and SR) to identify relevant studies that merited a full‐text review. The reviewers had 97% agreement on article inclusion/exclusion and a third reviewer (VA) resolved disagreements (*n* = 66). The first author (LRS) completed a full‐text review of the 362 articles that passed the initial screen, with 65 articles being selected for full‐text extraction. The reasons for exclusion at the full‐text screen are listed in Figure 2.

### 2.3 Data extraction

Two primary reviewers developed a standardized data extraction form to collect relevant information, including the year of publication, sample size, participant age and sex, baseline task, emotion task, method of heart rate collection and analyses, physiological results, behavioral coding analyses, relationship between behavioral and heart rate measures, and information on missing data/data loss. The development of the structured data extraction form was an iterative process that allowed for flexibility and comprehensiveness in data extraction (Colquhoun et al., 2014).

### 2.4 Statistical considerations

To simplify the results, all heart period values were converted to HR using the calculation HR (bpm) = 60,000/HP (value in msec; Fisher & Ritter, 1998), focusing on HR, HRV, and RSA. The effect of emotion‐evoking task on reactivity was determined by a difference score (i.e., baseline values of HR, HRV, or RSA were subtracted from emotion‐evoking task values of HR, RSA, or HRV).

Meta‐regressions on physiological parameters were completed in Stata (Stata Statistical Software, Release 15; StataCorp LP, College Station, TX) using the *metareg* command (Palmer & Sterne, 2016), comparing age at assessment and physiological measurement (HR and RSA) during the baseline and emotion‐evoking tasks. Weighting of each study was computed as the standard error (calculated during the meta‐analyses, described below), with the results expressed as regression coefficients and 95% confidence intervals (CI). Meta‐analyses on physiological parameters were completed in Stata using the *metan* command (Palmer & Sterne, 2016). Separate meta‐analyses were conducted for HR, RSA, and HRV, with differences between emotion‐evoking tasks explored using the *subgrouping* command. Cohen's *d* effect sizes (calculated using the following formula: *d* = *M*_1_ − *M*_2_/*s*_pooled_ where $s_{\text{pooled}}=\surd \left[\left(s_{1}^{2}+s_{2}^{2}\right)/2\right]$) and standard error were computed for each study (where data were available) and used in the meta‐analyses, with 0.2–0.49 = small effect, 0.5–0.79 = medium effect, and ≥0.8 = large effect (Cohen, 1988). Heterogeneity was examined using confidence intervals (CI), the *I2* statistic, and forest plots. The *I2* statistic, which ranges from 0% to 100%, is a measure of the variability in effect estimates due to heterogeneity between studies rather than chance (e.g., sampling error). Heterogeneity values are considered low at <25%, modest at 25%–50%, and high at >50%. Preliminary analyses suggested our meta‐analyses had *I^2^* statistics > 50%; thus, we conducted random effects meta‐analyses. Funnel plot, trim and fill analyses, and Egger's tests for small study effects were completed using the *metafunnel*, *metatrim, and metabias* commands in Stata (Palmer & Sterne, 2016) to investigate publication bias and heterogeneity through visual and statistical examination of the data (Egger et al., 1997).

Overall, 53 of the 64 articles were included in the meta‐analyses. Of the remaining 11 articles, insufficient data were available to calculate an effect size for baseline and/or emotion‐evoking task. Of the 53 studies included in the meta‐analyses, data from 21 studies were included twice. The rationale for duplication was to explore task, age, and measurement effects broadly. Data from the same study were included if they compared baseline to emotion‐evoking task (a) at different time points (e.g., ages; *n* = 9; Zeegers et al., 2017), (b) by characteristic (e.g., sex; *n* = 1; Eiden et al., 2018), (c) across different tasks (*n* = 6; e.g., Calkins et al., 1998a), or (d) provided both HR and RSA data (*n* = 7; e.g., Busuito et al., 2019). Note that one study (Calkins & Keane, 2004) provided data across different ages, tasks, and measurements.

### 2.5 Ethical statement

Ethics approval was not required for this study.

### 2.6 Data sharing

We have shared the tables generated from our review as .doc files for use by other authors (see Appendices [Link], [Link], [Link], [Link]).

## 3 RESULTS

The systematic review and meta‐analysis examining the relationship between physiological indices of heart rate during emotion‐evoking tasks in children aged four years and under resulted in the inclusion of 64 articles. The Results section is organized as follows: (a) a descriptive overview of the included articles, with location and sample size, as well as age, ethnicity, and socioeconomic status of participants; (b) an overview of data collection and analyses, including electrode number and placement, RSA calculations used (if applicable), neutral events between tasks, and data loss; (c) descriptions of the baseline tasks; (d) descriptions of emotion‐evoking tasks; (e) meta‐regressions on HR and RSA baseline and emotion‐evoking tasks by age; (e) meta‐analyses on HR, RSA, and HRV; and (f) associations between physiological measurements and behavioral coding. Due to the large number of included articles, citations are presented in their respective tables and referenced in the text by category, unless specifically described in the text.

### 3.1 Overview of included articles

No language or publication date limits were placed during the search, yet all included articles were published in English and the earliest article meeting inclusion criteria was published in 1975 with the most recent published in 2019. The articles originated from the United States (*n* = 46), Europe (*n* = 9), Canada (*n* = 6), Israel (*n* = 2), and the Netherlands (*n* = 2), with 53 studies involving a cross‐section (single time point) methodological design and the remaining 11 studies being longitudinal (multiple time points) in design. Sample sizes ranged from 12 (Ham & Tronick, 2006) to 278 (Zeytinoglu et al., 2019). Descriptive details of the included studies are presented in Appendix S2.

Briefly, of the 64 studies, 19 included children under 6 months of age, 33 included children between 6 and 12 months of age, 16 included children between 13 and 24 months of age, 10 included children between 2 years and 3 years 11 months, and 5 included children between 4 years and 4 years 11 months. The studies consisted of primarily Caucasian participants from middle‐class backgrounds, with 13 studies not reporting on ethnicity descriptors and 24 studies not reporting on socioeconomic status.

### 3.2 Data collection and analyses summary

#### 3.2.1 Number and placement of electrodes

When considering the number of electrodes used, 3% of studies used 4 electrodes, 49% of studies used 3 electrodes, 23% used 2 electrodes, and 25% did not report on the number of electrodes attached to the child. Of the studies that reported placement of sensors, 94% reported that they were attached to differing areas on the chest.

#### 3.2.2 RSA calculation

Of the 46 studies that reported the method of RSA or HRV calculation, 30 (65.2%) used Porges' method (i.e., respiration bandwidth of 0.24–1.04 Hz) or the use of MXedit software (incorporated Porges' method into its calculation of RSA; Porges, 1985, 1985a). Of the remaining 16 studies, 8 (17.4%) mentioned the use of respiration bandwidth in their calculations of RSA, 5 of which used the “infant bandwidth” of 0.24–1.04 Hz, and 3 using different bandwidths in their calculations (0.30–0.75 or 0.24–0.40). The remaining 8 (17.4%) studies calculated HRV based on IBI data unfiltered by a respiration bandwidth.

#### 3.2.3 Neutral events between tasks

Of the 64 studies that analyzed the effect of emotion‐evoking tasks on physiological reactivity in children aged four and under, 53% (*n* = 34) did not mention the use of a “pause,” “break,” “inter‐trial interval,” or “return to resting baseline” in their methodological descriptions. Of the remaining studies, 25% (*n* = 16) employed the face‐to‐face/still‐face paradigm, in which cardiac measures during face‐to‐face play could be compared to those collected during reunion (i.e., a recovery period), although no mention of “pause,” “break,” “inter‐trial interval,” or “return to resting baseline” was found in their methodological descriptions. Fourteen studies (22%) did include a “pause,” “break,” “inter‐trial interval,” or “return to resting baseline” in their methodological descriptions. Of these, 11 mentioned including a short break, but did not provide nor use these data in their analyses; one included a “postrecovery phase,” but did not provide or use the data in their analyses (Fracasso et al., 1994); one included an “inter‐trial interval” to allow a return to baseline before the next task (but did not provide the data; Campos et al., 1975); and one included a 30‐s interval between tasks, with data provided and used in analyses (Provost & Gouin‐Decarie, 1979).

#### 3.2.4 Data loss and reasons

Of the 64 studies, 12.5% (*n* = 8) did not report the percentage of data loss nor the reasons for data loss. An additional 15.6% of studies (*n* = 10) did report percentage of data loss experienced, but did not provide reasons why data loss occurred. Of the studies that reported the amount of data loss (*n* = 56), 8.9% reported less than 10% data loss, 44.6% reported data loss between 11% and 20%, and 46.4% reported data loss greater than 20%. Reasons provided for loss of data included equipment failure, refusal to wear electrodes, artifacts in the data, child distress/refusal to continue, and human error.

### 3.3 Baseline (resting) tasks

Baseline tasks varied in duration from 5 s (Anderson et al., 1999; Bohlin & Hagekull, 1993; Skarin, 1977) to 420 s (Busuito et al., 2019), fell within five categories, as differentiated for the purpose of this review and categorized on the nature of the activities, described in Table 1, plus a category described as “baseline period” (*n* = 5) that ranged from 60 to 180 s.

**Table 1:** Baseline tasks characteristics

Article	Sample size, age assessed	Baseline description	Length (Epochs)	Measurement	Mean (*SD*)
*Unknown task (n = 5)*					
Hay et al. (2017)	*n* = 275, 12 m	"Baseline period"	180 s (15 s epochs)	Mean HR	132.81 (21.04) bpm
Santesso et al. (2007)	*n* = 39, 9 m	"Baseline period"	60 s (na)	Mean HP (to HR)	138 (NA) bpm
Schmidt et al. (2003)	*n* = 33 to 52 at 3, 6, 9, and 12 m	"Baseline period"	60 s (na)	Mean HP (to HR)	3 m: 147 (15.53) bpm; 6 m: 144 (11.74) bpm; 9 m: 136 (11.42) bpm; 12 m: 131 (11.56) bpm
Stone and Porter (2013)	*n* = 101, 6 m	"Baseline period"	180 s (na)	Mean HP and RSA	Bradycardia: 154 (12.08) bpm; NonBradycardia: 151 (11.88) bpm
Zeegers et al. (2017)	*n* = 135, 4 and 12 m	"Baseline period"	120 s (na)	Mean HRV	4 m: 16.57 (9.85) msec; 12 m: 29.88 (16.75) msec
*Sitting quietly (n = 12)*					
Busuito and Moore (2017)	*n* = 53, 6 m	Infants sat quietly in chair across from mothers while mother read description of study	180 s (30 s epochs)	Mean RSA	3.65 (1.07) In(msec2)
Calkins and Fox (1992)	*n* = 50, 5 m; *n* = 52, 14 m; *n* = 48; 24 m	Infant sat in mother's lap	300 s (na)	Mean HP (to HR) and RSA	NA
Campos et al. (1975)	*n* = 80, 5 m; *n* = 40, 9	Resting period	240–300 s (3 s epochs)	Mean HR	NA
Fracasso et al. (1994)	*n* = 58, 5 m; *n* = 53, 7 m; *n* = 44, 10 m; *n* = 49, 13 m	Infants sat on mothers’ laps in awake, quiet, and attentive state	300 s (30 s epochs)	Mean HP (to HR) and RSA	5 m: 142 (9.76) bpm; 7 m: 138 (9.90) bpm; 10 m: 135 (10.30) bpm; 13 m: 136 (10.80) bpm; 5 m: 3.02 (0.71) In(msec2); 7 m: 3.25 (0.72) In(msec2); 10 m: 3.27 (0.72) In(msec2); 13 m: 3.20 (0.71) In(msec2)
Holochwost et al. (2014)	*n* = 95, 6 m	Child seated facing away from mother on her lap	120–240 s (15 s epochs)	Mean RSA	3.72 (0.91) In(msec2)
Johnson et al. (2014)	*n* = 41, 6 m	Infant in car seat in quiet, low‐lit room while mother sat behind occlusion screen	15 s (na)	Mean RSA	3.58 (1.76) In(msec2)
Moore (2009)	*n* = 48, 6 m	Sit quietly	180 s (30 s epochs)	Mean RSA	NA
Moore and Calkins (2004)	*n* = 72, 3 m	Minimize stimulation, infants not receiving attention from mothers or toys	180 s (30 s epochs)	Mean HR and RSA	146 (10.89) bpm; 2.82 (0.75) In(msec2)
Moore et al. (2009)	*n* = 152, 6 m	Minimize stimulation, infants not receiving attention from mothers or toys	120 s (15 s epochs)	Mean HP (to HR) and RSA	3.68 (0.85) In(msec2)
Rash et al. (2015)	*n* = 194, 6 m	Seated on mother's lap, with movement/interaction at a minimum.	180 s (45 s epochs)	Mean RSA	461.93 (425.52) msec2/Hz
Rash et al. (2016)	*n* = 254, 6 m	Seated on mother's lap, with movement/interaction at a minimum.	180 s (45 s epochs)	Mean RSA	495.31 (402.72) msec2/Hz
Vaughn and Sroufe (1979)	*n* = 16, 8−16 m	Seated in high chair	180 s (na)	Mean HR	130 (NA) bpm
*Sedentary task (n = 5)*					
Busuito et al. (2019)	*n* = 140, 6 m	Infants seated in mother's lap, mother's asked to show/read book to infants.	420 s (30 s epochs)	Mean HP (to HR) and RSA	138 (9.79) bpm; 3.18 (0.85) In(msec2)
Cho and Buss (2017)	*n* = 62, 24 m	Engaged in quiet, sedentary activities (e.g., coloring) with experimenter.	300 s (30 s epochs)	Mean RSA	4.46(1.07) In(msec2)
Dawson et al. (2001)	*n* = 159, 13−15 m	Experimenter blew soap bubbles from behind black curtain	60 s (na)	Mean HR	128 (NA) bpm
Perry et al. (2016)	*n* = 230, 5 or 10 m	Seated with mother, watching RA manipulate toy with brightly colored balls	60 s (na)	Mean RSA	5 m: 3.88 (1.18) In(msec2); 10 m: 4.61 (1.10) In(msec2)
Scrimgeour et al. (2016)	*n* = 125, 42 m	Children sat quietly while coloring or reading a book with experimenter	NA (30 s epochs)	Mean RSA	NA
*Face‐to‐face episode/Play (n = 15)*					
Bush et al. (2017)	*n*−135, 6 m	Free play episode	120 s (30 s epochs)	Mean RSA	4.27 (1.04) In(msec2)
Baker et al. (2012)	*n* = 70, 12, 24, and 36 m	Mother and child quietly playing together	210 s (30 s epochs)	Mean HR	12 m: 136.43 (12.15) bpm; 24 m: 121.86(9.74) bpm; 36 m: 113.72 (9.84) bpm
Bazhenova et al. (2007)	*n* = 16, 4 m	Mother and child quietly playing together	210 s (30 s epochs)	Mean HP (to HR) and RSA	155 (7.75) bpm; 3.2 (0.3) In(msec2)
Feldman et al. (2010)	*n* = 53, 6 m	Free play episode	120 s (na)	Mean RSA	Touch: 3.56 (0.85) In(msec2); No Touch 3.65 (0.74) In(msec2)
Gray et al. (2017)	*n* = 167, 4 m	Free play episode	120 s (na)	Mean RSA	2.7 (0.47) In(msec2)
Haley and Stansbury (2003)	*n* = 43, 5 m	Free play episode	120 s (na)	Mean HR	146.60(11.92) bpm
Ham and Tronick (2006)	*n* = 12, 5 m	Free play episode	120 s (na)	Mean HR and RSA	Recovered: 145 bpm, 3.3 [units unknown]; Stable: 142 bpm, 3.7 [units unknown]; Dysregulated: 137 bpm, 3.5 [units unknown]; Protest: 152 bpm, 3.5 [units unknown]
Ham and Tronick (2009)	*n* = 18, 5 m	Free play episode	120 s (na)	Mean HR and RSA	143.57 (9.97) bpm
Hill‐Soderlund et al. (2008)	*n* = 132, 14 m	Free play episode	180 s (30 s epochs)	Mean RSA	3.70 (1.03) In(msec2)
Mireault et al. (2018)	*n* = 37, 5, 6, and 7 m; *n* = 46, 4, 6, and 8 m	"Ordinary play"	NA (45 s epoch)	Mean HR	139.4 (12.43) bpm
Pratt et al. (2015)	*n* = 122; 5 m	Free play episode	180 s (15 s epochs)	Mean RSA	NA
Provenzi et al. (2015)	*n* = 94, 4 m	Free play episode	120 s (10 s epochs)	Mean RSA	NA
Qu and Leerkes (2018)	*n* = 206, 14 m	Free play episode	120 s (15 s epochs)	Mean RSA	3.65 (0.99) In(msec2)
Spangler and Grossmann (1993)	*n* = 41, 12 m	Stranger approach episode 2	NA (na)	Mean HR	Insecure: 142.3 (18.2) bpm; Disorganized: 134.2 (6.9) bpm; Secure: 140.7(12.0) bpm
Weinberg and Tronick (1996)	*n* = 50, 6 m	Free play episode	120 s (10 s epochs)	Mean HR and RSA	138.20 (NA) bpm, 3.165 (NA) In(msec2)
*Before/Between tasks (n = 5)*					
Anderson et al. (1999)	*n* = 45, 5 and 10 m	Episode preceding the entrance of the stranger	5 s	Mean HR	NA
Bohlin and Hagekull (1993)	*n* = 31, 10−13 m	Episode preceding the entrance of the stranger	5 s (na)	Mean HR	Mpres: 130.2 bpm; Mabs: 146.8 bpm
Provost and Gouin‐Decarie (1979)	*n* = 40, 9−12 m	Immediately before each episode	15 s (5 s epochs)	Mean HR	With Mother: 147.2 bpm; Frustration: 148.5(NA) bpm; Isolation: 147.7 (NA) bpm; Reunion: 156.4 (NA) bpm
Skarin (1977)	*n* = 32, 5–7 or 10−12 m	Immediately before each approach step	5 s (500 msec epochs)	Mean HR	NA
Waters et al. (1975)	*n* = 26, 5 and 7 m	Immediately before task	10 s (na)	Mean HR	120 (NA) bpm
*Watched video (n = 22)*					
Blankson et al. (2012)	*n* = 263, 40 m	Watched video (Spot)	300 s (na)	Mean RSA	6.41 (1.32) In(msec2)
Brooker et al. (2013)	*n* = 124, 6 m	Watched video (Baby Mugs)	not provided (30 s epochs)	Mean RSA	3.55 (0.77) In(msec2)
Buss et al. (2004)	*n* = 80, 24 m	Watched video (Baby Mugs)	300 s (30 s epochs)	Mean HR and RSA	119.03 (8.53) bpm, 5.06 (0.95) In(msec2)
Buss et al. (2005)	*n* = 68, 24 m	Watched video (Baby Mugs)	300 s (30 s epochs)	Mean HP (to HR) and RSA	119.03 (8.53) bpm, 5.06 (0.95) In(msec2)
Calkins (1997)	*n* = 41, 24−36 m	Watched video (Spot)	300 s (30 s epochs)	Mean RSA	5.66 (NA) In(msec2)
Calkins and Dedmon (2000)	*n* = 50, 24 m	Watched video (Spot)	300 s (30 s epochs)	Mean HP (to HR) and RSA	5.53 (NA) In(msec2)
Calkins and Johnson (1998b)	*n* = 73, 18 m	Watched video (Barney)	300 s (30 s epochs)	Mean HP (to HR) and RSA	NA
Calkins and Keane (2004)	*n* = 154, 24 and 54 m	Watched video (Spot)	300 s (30 s epochs)	Mean HP (to HR) and RSA	24 m: 109 (10.89) bpm; 5.76 (1.4) In(msec2); 54 m: 97 (12.86) bpm, 5.95 (1.35) In(msec2);
Calkins et al. (1998a)	*n* = 65, 24 m	Watched video (Spot)	300 s (30 s epochs)	Mean RSA	5.36 (1.22) In(msec2)
Eiden et al. (2018)	*n* = 69, 9 m	Watched video (Baby Einstein)	180 s (na)	Mean RSA	Boys: 0.016 (0.01) sec; Girls: 0.02 (0.01) sec
Eisenberg et al. (2012)	*n* = 213, 18, 30, 42, and 54 m	Watched video (Neutral/smiling babies with cheerful music)	181 s (na)	Mean RSA	0.02 (0.01) sec
Gilissen et al. (2007)	*n* = 78, 36−48 m	Watched video (Tik Tak # 15)	90 s (na)	Mean HRV	Alone: 0.66 (12.6); With Parent: 0.50 (11.12); [units unknown]
Gilissen et al. (2008)	*n* = 78, 48 m	Watched video (Tik Tak # 15)	90 s (na)	Mean HRV	0.66 (12.69) [units unknown]
Liew et al. (2011)	*n* = 247, 18 and 30 m	Watched video (NA)	NA (na)	Mean RSA	18 m:0 0.19 (0.11); 30 m: 0.30 (0.17) [units unknown, multiplied by constant of 10]
Morasch and Bell (2012)	*n* = 106, 5 and 10 m	Watched video (Sesame Street)	45 s (na)	Mean HR and HRV	NA
Noten et al. (2019a)	*n* = 61, 45 months	Watched video (calm music and abstract animations)	60 s (na)	Mean HR	Happy: 107.74 (10.86) bmp, Sad: 105.46 (10.48) bpm, Fear: 106.25 (11.17) bmp
Noten et al. (2019b)	*n* = 125, 6 m	Watched video (relaxing movie)	120 s	RSA	Distress: 3.39 (0.38) In(msec2); Frustration: 3.3 (0.40) In(msec2)
Paret et al. (2015)	*n* = 48, 36−48 m	Watched video (NA)	120 s (15 s epochs)	Mean HR and RSA	103.47 (10.21) bpm, 7.37 (1.08) msec2
Perry et al. (2012)	*n* = 197, 36−48 m	Watched video (NA)	300 s (30 s epochs)	Mean RSA	6.60 (1.12) In(msec2)
Wagner et al. (2018a)	*n* = 108, 24 m	Watched video (NA)	300 s (na)	Mean RSA	4.86 (0.98) In(msec2)
Wagner et al. (2018b)	*n* = 88, 48 m	Watched video (NA)	120 s (120 s epoch)	Mean RSA	6.20 (1.18) In(msec2)
Zeytinoglu et al. (2019)	*n* = 278, 56 m	Watched video (swimming colorful fish)	120 s (30 s epochs)	Mean RSA	7.21 (1.11) In(msec2)

The first category of baseline tasks involved *sitting quietly* without toys (*n* = 12) either alone or with the mother and ranged from 15 to 300 s. The second category involved a *sedentary task* (*n* = 5) in which the child played with toys alone, watched an examiner play with a toy, or read a book with their mother or the examiner for 60–420 s. The third category involved *play with the mother* (*n* = 15) and was often the first play episode of still‐face or stranger approach or another type of play and ranged from 120 to 210 s. The fourth category included the *period immediately before* (*n* = 5) the emotion‐evoking task or an inter‐trial interval and ranged from 5 to 15 s. Finally, the fifth category consisted of *watching a video* (*n* = 22). Videos included “Spot”—a dog that explores his world; “Baby Mugs”—a parade of baby faces, drooling, giggling, yawning, etc.; “TikTak #15”—a series of short clips geared toward toddlers; “Sesame Street,” “Barney the Dinosaur,” ”Baby Einstein,” or other short unnamed videos, ranging from 45 to 300 s. Watching a video was separated from sedentary tasks as it involved screen time, rather than solitary play with toys or with a second person, and thus may have a different unknown effect on heart rate.

### 3.4 Emotion‐evoking tasks

The emotion‐evoking tasks *probed for various emotional responses,* classified by the category identified by the investigators in each study. These include anger (*n* = 2), disappointment (*n* = 1), distress (*n* = 31), fear (*n* = 23), frustration (*n* = 13), guilt (*n* = 1), “negative” (*n* = 2), and “positive” (*n* = 13). The emotion‐evoking tasks that probed for **“** *Distress”* included arm restraint, still‐face paradigm, parental separation, or distressing audio or video of a crying child; “*Fear”* included a stranger situation, fear‐relevant stimuli (e.g., masks), unpredictable toys, and videos; “*Frustration”* included toy removal or blocking, food denial, frustrating games, or drawing tasks; “*Anger”* included a narrated comic strip; “*Guilt”* included a mishap guilt paradigm; “*Disappointment”* included a disappointment task; “*Negative”* included a “negative response” video task; and “*Positive”* included puppet play, smiling faces, music, comforting audio, video of a happy child, and absurd events.

Because children may have varied (or nonprobed) reactions to probed emotions (e.g., laugh at a scary stimuli), the results will be summarized based on task used (e.g., arm restraint) rather than probed emotion (e.g., distress).The emotion‐evoking tasks are described in Table 2, the methods used to collect physiological data are presented in Table 3, and the results of the physiological tasks are presented in Table 4.

**Table 2:** Descriptions of emotional‐evoking tasks

Probed emotion	Task	Description	Duration	Article using task
Anger	Narrated Comic Strip	Children watched narrated comic strip film which presented multiple emotionally salient stories; Two anger‐inducing portions of video depicted child expressing anger toward antagonistic peer and child having heated argument with her/his mother; Both scenarios were accompanied by dramatic music and had positive resolution	Two 60 s clips	Wagner et al. (2018b)
Disappointment	Disappointment Task	Three phases: (1) Waiting for Gift—experimenter announced that prize had been earned following other task completion; experimenter left room and returned with wrapped box containing prize that had been ranked as least desired; (2) Wrong Gift—experimenter maintained neutral expression and detached demeanor as undesirable gift was opened, remained in the room, and then exited. Sixty seconds later, second experimenter entered, feigned surprise and asked how child felt when received least‐desired prize; (3) Resolution—experimenter re‐entered the room, explained the mix‐up with prizes and gave child the opportunity to exchange the prize	Waiting for Gift 30 s; Wrong Gift 40 s; Resolution 60 s	Scrimgeour et al. (2016)
Distress	Arm Restraint	Caregiver (Calkins & Fox, 1992) or Researcher (Johnson et al., 2014) hold child's arms at their side (discontinued if child cries)	Each trial 120 s	Calkins and Fox (1992), Johnson et al. (2014), Perry et al. (2016)
Distress	Arm Restraint—Modified 1	Mother stood behind child and gently grasped child forearms and firmly hold them to their side while an attractive toy was placed directly in front of child; Recovery period followed a second trial with child playing with toy or comforted by parent	Arm restraint 30 s; Recovery 15 s	Rash et al. (2015), Rash et al. (2016)
Distress	Arm Restraint—Modified 2	Mothers instructed to gently hold child's arms to their sides of the child and then to release the arms while maintaining a still‐face with no verbal interactions.	Both trials 90 s	Stone and Porter (2013)
Distress	Arm Restraint—Modified 3	During toy removal task, mother engaged her child in play with an interesting toy; mother then held toy out of child's reach, retaining eye contact but silent with still‐face. Mother next gently restrained her child's arms against his/her sides while maintaining a still‐face and silence.	Toy play 30 s; toy removal and arm restraint 120 s	Morasch and Bell (2012)
Distress	Arm Restraint and Toy Play	Child was first presented and encouraged to play with an attractive toy; experimenter stood behind child, placed hands on the child's forearms and moved them to child's sides and held them while maintaining a neutral expression; After first trial, child was allowed to play with the toy again followed by a second arm restraint. The child was again allowed to play with the toy after arm restraint	Toy presentation and Arm restraint each 30 s	Eiden et al. (2018)
Distress	Distressing Audio or Video	Child looked at books while audiotape of a crying toddler was played just outside the playroom door (at age 2) or child was shown a videotape in which a young child experiences the death of a pet (age 4)	Audio was 120 s; Video was 240 s	Calkins and Dedmon (2000), Calkins and Keane (2004)
Distress	still‐face Paradigm	During Play, parent plays with child without toys. During Still‐Face, parent maintains a neutral expression and does not touch or interact with child. During Reunion, there is a resumption of play with parent responding to child; Feldman et al. (2010) had a 3‐min Play; Gray et al. (2017) had 2.5‐min episodes for each phase	Each episode 120 s	Bush et al. (2017), Busuito and Moore (2017), Busuito et al. (2019), Gray et al. (2017; Ham and Tronick (2006), Holochwost et al. (2014), Moore and Calkins (2004), Moore (2009), Moore et al. (2009), Provenzi et al. (2015), Qu and Leerkes (2018), Weinberg and Tronick (1996), Noten et al. (2019b)
Distress	Still‐Face Paradigm Modified	Second Still‐Face and Reunion added. During Play, parent played with child and were given a toy. Parents did not touch their child during the procedure. During Still‐Face, parents expressed a neutral expression, remained still, and looked slightly above child's head to avoid eye contact. During Reunion, there is a resumption of play with parent responding to child	Each episode 120 s	Haley and Stansbury (2003)
Distress	Still‐Face with Touch	During Play, parent plays with child without toys. During Still‐Face, parent maintains a neutral expression and does not touch or interact with child. Mother may be asked to provide tactile contact during Still‐Face. During Reunion, there is a resumption of play with parent responding to child	Each episode 180 s	Feldman et al. (2010)
Distress	Still‐Face with Touch or Arm Restraint	During Play, parent plays with child without toys. During Still‐Face, parent maintains a neutral expression and does not touch or interact with child. Mother may be asked to provide tactile contact or restrain arms of child during Still‐Face. During Reunion, there is a resumption of play with parent responding to child	Play 180 s; still‐face and Reunion 120 s	Pratt et al. (2015)
Distress	Strange Situation Modified	Play with parent, followed by a brief separation from mother, and then a reunion	Play 600 s; Separation 180 s; Reunion 300 s	Calkins and Fox (1992)
Distress	Teddy Bear Picnic	Two costumed characters (the “Birthday Lady” and the “Teddy Bear”): Birthday Lady encouraged children to sit around picnic mat and play with plastic food and parents return to sofa in room. The Teddy Bear entered, pausing in doorway until each child had seen him. Birthday Lady invited Teddy Bear to sit down near picnic blanket, and he offered each child a piece of plastic birthday cake. Birthday Lady and Bear then danced while singing “Round and Round the Garden” before offering each child, with the help of their parents, the opportunity to dance with Bear. Birthday Lady instructed families to let children play in any way they would like and then she left the room. .	NA	Hay et al. (2017)
Distress	Videos—Crying	Children seated in high chair with mother's present; video depicted babies crying	Clip 42 s	Eisenberg et al. (2012), Liew et al. (2011)
Fear	Interesting but Scary	Mothers opened cupboard containing witch‐like mask with speaker inserted behind it; Mother shown mask prior and asked to act as normally would if child became frightened; Experimenter, from another room, engaged child in conversation (via the mask) in a friendly voice. First, examiner asked child about toys in which he or she had played then invited child to touch her nose (the mask)	120 s	Paret et al. (2015)
Fear	Multiple Stranger Approach	Using four different combinations, Stranger 1 enters and departs, then Stranger 2 enters and departs. Mother departs and Mother re‐enters	Four 15 s phases	Campos et al. (1975)
Fear	Spider	Experimenter presented child with large, realistic, moving spider and encouraged child to touch it	120 s	Calkins and Dedmon (2000)
Fear	Strange Situation—Ainsworth	Child seated in high chair and completed seven episodes: mother and child together, stranger enters room with mother and child, mother leaves stranger and child alone, stranger leaves and mother and child together, mother leaves and child is alone, stranger re‐enters with child, and stranger leaves and mother returns with child.	NA	Hill‐Soderlund et al. (2008), Spangler and Grossmann (1993)
Fear	Stranger Approach Modified	Child seated and affectively neutral, male stranger entered; Stranger paused near door before approaching half of distance toward child; Stranger then paused, addressed child, approached rest of the distance to child, and knelt near him/her for period of time; At the end of this period, stranger rose and exited room	Knelt 120 s	Brooker et al. (2013), Buss et al. (2004), Buss et al. (2005), Skarin (1977)
Fear	Stranger Approach Modified 3	Mothers seated on chair behind child; During task, male stranger approached and talked to the child and then picked up and held child	Talking 30 s; Holding 30 s	Zeegers et al. (2017)
Fear	Stranger Challenge	Male stranger entered room and stared at child without speaking then left the room	Episode each 60 s	Wagner et al. (2018a)
Fear	Stranger Wariness	Mother put child in high chair; Female stranger entered room and approached child in a standard stepwise fashion consisting of six episodes (pause and call child's name 1, pause and call child's name 2, approach, pause at a distance of 1 meter, reach out to child, and touch child); Stranger then left room without any comments	Six episodes each 5 s (30 s total)	Anderson et al. (1999), Bohlin and Hagekull (1993), Waters et al. (1975)
Fear	Unpredictable Mechanical Toy	Mother leaves room and unfamiliar experimenter enters and placed robot 1.5 m away from child; Experimenter makes robot approach child, stopping 15 cm from child, while making movements with its arms and emitting noise. The robot then walks backward and stops at back of room for 10 s before moving forward again; This was repeated three times	NA	Baker et al. (2012)
Fear	Video	Episodes contained one neutral and one fear‐inducing video clip; Neutral clips were from a videotape (Tik Tak 15), which showed colorful moving shapes; Fear‐inducing clips chosen from movie “Dinosaur,” (Walt Disney); Children watched two episodes; one with parent and one alone; Clips were counterbalanced	Each clip 60 s	Gilissen et al. (2007), Gilissen et al. (2008)
Frustration	Food Denial	Experimenter entered room and placed sealed crackers (in a plastic bag) on sofa and told child that crackers could not be eaten until play was finished	120 s	Calkins and Johnson (1998b), Calkins and Dedmon (2000)
Frustration	Frustrating Puzzle Task	Child given a wood toy with many holes with string laced through the holes (middle of string was glued to inside of toy, making it impossible to untangle completely). Experimenter asked child to untangle toy while he/she worked on paperwork in other room. The experimenter left the room and upon return, experimenter presented second unglued puzzle to child and allowed child to completely unlace string and solve puzzle	180 s	Perry et al. (2012)
Frustration	Green Circles	Experimenter repeatedly asks child to draw circles with green marker. Experimenter criticizes child's circles but does not say how to do better. Experimenter continues to prompt, “I need the perfect green circle” for the duration of the task	240 s	Blankson et al. (2012)
Frustration	High Chair	Experimenter placed child in high chair and told child to wait for a special toy; mother sat nearby with magazine and responded normally to child if child spoke to her but did not remove child from chair	300 s	Calkins and Johnson (1998b)
Frustration	Locked Box with Snack	Experimenter placed clear plastic container of cookies on table that child is unable to open and left room; Child was free to manipulate container while experimenter was gone; Mother was instructed not to open container	120 s	Calkins and Keane (2004) (@ 2 years)
Frustration	Locked Box with Toy	Child offered choice of two highly desirable toys; after child makes selection, toy is placed in transparent box that is locked with a padlock. After showing child how to open lock with key, experimenter gave child large ring of keys, none of which was the correct key, and told child to open box to get toy. Experimenter leaves room while child attempts to open box and re‐enters to present child with correct key. Child then opens box and plays with toy	240 s	Blankson et al. (2012), Calkins and Keane (2004) (@ 4.5 years); Zeytinoglu et al. (2019)
Frustration	Plexiglass Barrier	Experimenter and child play with musical telephone; After play, experimenter took toy away and placed it behind Plexiglas barrier out of child's reach	Play 60 s; Removal 120 s	Calkins and Johnson (1998b)
Frustration	Plexiglass Barrier 2	Child engaged with stuffed rabbit. After play, experimenter placed plexiglass barrier in front of child and asked parent to remove toy from child and place it behind barrier; Toy remained behind barrier and then child was allowed to play with it again; repeated two additional times	Play 15 s; Removal 30 s	Rash et al. (2015), Rash et al. (2016)
Frustration	Toy Removal 1	After child played with toy for a few minutes, parent takes toy away saying, “I don't want you to play with this anymore.” For task, mother stands up and places toy on shelf directly in front of child and sits back down in her chair; toy then returned to child	Removal 30 s; Returned 60 s	Buss et al. (2005), Calkins et al. (1998a), Calkins and Johnson (1998b)
Frustration	Toy Removal 2	Experimenter takes toy away from child and plays with it for 2 min, commenting on how fun it is to play with	120 s	Zeytinoglu et al. (2019)
Frustration	Toy Removal in Box	Experimenter gave child attractive electronic musical toy to play with; Experimenter then tasked toy away, placed it in clear plastic box that child was not able to open, and put box on the table in front of child	Play 60 s; Removal 120 s	Calkins (1997)
Frustration	Toy Retraction	Child played with novel toy, after which mother removed toy from child's reach; Toy then returned to the child; repeated two additional times	Play 15 s; Removal 30 s	Rash et al. (2015), Rash et al. (2016)
Frustration	Car Seat Task	Child was bucked into car seat and mother stood behind child (who the child could see if they turned their head)	60 s	Noten et al. (2019b)
Guilt	Mishap Guilt Paradigm	Child presented with tower, which experimenter says is her favorite toy and had made it herself; She told child that she would share it as long as they were very careful. Because tower is rigged, it fell apart as soon as child began to handle it; Experimenter then says “Oh my” with regret and sits still in front of child with her face covered with her hands She asked, “What happened?”, “Who did it?”, and “Did you do it?”; Child is told that it was not their fault and there was a problem with tower. She gives partially built tower to child and asks child to help her make it; Experimenter tells child damage was not their fault and assumed responsibility for it	Face covering 30 s; Other parts NA	Baker et al. (2012)
Positive	Absurd Event	Research assistant showed parents two ordinary events (narration of playing with a ball/drinking from a cup and read a book) and two absurd events (ball worn as a clown nose and continuously poked while saying “beep” and book/cup worn like hat and continuously raised and lowered while saying “zoop”). Each absurd event was presented twice, once with parents holding neutral affect and once with positive affect (i.e., smiling and laughing). Parental affect was not manipulated during ordinary events	Each event 45 s	Mireault et al. (2018)
Positive	Peek‐a‐Boo Play	Mother played peek‐a‐boo with child and familiar experimenter played peek‐a‐boo	Each session 60 s	Dawson et al. (2001)
Positive	Puppet Play	Game of peek‐a‐boo with a puppet named Spot	NA	Calkins et al. (1998a), Calkins and Dedmon (2000), Calkins (1997), Cho and Buss (2017)
Positive, Angry, Neutral	Emotion‐Evoking Task	Mothers instructed to turn toward experimenter while experimenter enacted script directed toward them in angry, excited, or neutral tone of voice; Same script used for each emotion; Mothers were instructed not to respond in any way	Episodes each 60 s	Moore (2009)
Positive, Fear	Audio—ID speech	Mothers expressed either comfort, surprise, or fear as they said “Hey, honey, come over here” to their children; Conditions were constructed such that samples expressing that emotion were played in random order	Episode each 60 s with 30 s inter‐trial pause	Santesso et al. (2007)
Positive, Fear	Peek‐a‐boo then scary mask	Child and mother played peek‐a‐boo; After child was positively engaged, mother called child name and appeared from behind screen wearing a full‐face mask; She then returned behind screen and repeated procedure; On second trial, stranger wore mask, after which, mask was removed and stranger approached child	NA	Vaughn and Sroufe (1979)
Positive, Fear, Frustration	Strange Situation and Toy Box	Six episodes: (1) exploration—child explores with mom in room, (2) play with mom—mom shows child how pull toy works, (3) frustration—mom puts toys in box and restrains child on his back, (4) reaction to stranger—experimenter approaches child, (5) isolation—child is left alone in room, and (6) reunion—mom comes back in with child and shows child how the pull toy works	Episodes each 180 s	Provost and Gouin‐Decarie (1979)
Positive, Fear, Sadness	Musical Pieces	Three orchestra excerpts that are known to vary in affective valence and intensity: Adagio by Barber reflected sadness; Peter and the Wolf by Prokofiev reflected fear; and Spring by Vivaldi (second movement) reflected joy	Pieces each 30 s	Schmidt et al. (2003)
Positive, Negative	Videos	Children placed in seat and shown variety of stimuli designed to elicit both positive and negative emotions	20 min (7, 10 months); 30 min (13 months)	Fracasso et al. (1994)
Positive, Neutral	Smiling/Blank Face	Female experimenter sat facing child and completed two trials: (1) Smiling Face—experimenter smiled while looking at child without moving her head, touching, or speaking to engage child, (2) Blank Face—experimenter stopped smiling and held a blank face while looking at child (discontinued if child became distressed)	Smiling Face 30 s; Blank Face 50–130 s	Bazhenova et al. (2007)
Positive, Sadness, Fear	Videos	Children watched three different videos (presented in random order); happy (a happy child opening a gift), fear (a child being scared by a toy spider), and sadness (a sad child flushing his dead goldfish down the toilet)	Each video clip was 50 s	Noten et al. (2019a)

**Table 3:** Description of physiological measurement

First author and year	Emotion/Emotion Task/Baseline Task	Electrode Number/Placement	Num/Duration of epochs	Measurement(s)	HR, HP, HRV, or RSA description	Data loss	Data editing	Behavioral coding
Anderson et al. (1999)^a^	**Fear**/Stranger Wariness/Before	3 electrodes/chest	8/5 s	Mean HR for eight episodes	Mean HR in beats per minute	20%–25% loss across tasks at 5 months and 23%–25% at 10 months due to incomplete data, equipment failure, or refusal of electrodes	Artifacts handled by abbreviating scoring period to leave a minimum of three artifact free seconds	Verbal and physical fear
Baker et al. (2012)^a^	**Fear and Guilt**/Unpredictable Toy and Mishap Guilt Paradigm/Play	3 electrodes/back	7/30 s	Mean HR for fear and guilt tasks	Mean HR in beats per minute	0%–13% loss across tasks due to equipment failure or refusal to participate	Data imputation was implemented using linear trend computation	Bodily and facial tension and fear and distress vocalizations
Bazhenova et al. (2007)^a^, ^b^	**Positive and Neutral**/Smiling Face and Blank Face/Play	3 electrodes/chest	1−4/30 s	Mean HP and RSA for Blank and Smiling episode	ECG signal amplified and HP times to nearest millisecond and sampled every 250 msec; RSA quantified using Porges method with MXedit Software (respiration bandwidth range of 0.24–1.04)	19% loss due to artifacts	Editing consisted of visual detection of outlier points followed by integer division or summation in MXedit Software	Gaze, affect, motor movements
Blankson et al. (2012)^b^	**Frustration**/Locked Box Task and Green Circle Task/Video	2 electrodes/chest	NA/NA	Mean RSA and Vagal Withdrawal for frustration task	Quantified using Porges method with MXedit Software (respiration bandwidth range not provided)	% data loss not reported	Edited and corrected by visual inspection using MXedit Software	Verbal and physical frustration and global regulation
Bohlin & Hagekull, 1993)^a^	**Fear/**Stranger Wariness/Before	3 electrodes/chest	7/5 s	Mean HR for each of the seven episodes	Mean HR in beats per minute	6% loss due to equipment failure	Artifacts controlled for by excluding all segments showing evidence of body movements and substituting them with the mean of their adjacent values	Verbal and physical fear
Brooker et al. (2013)^b^	**Fear**/Stranger Approach/Video	NA/NA	NA/30 s	Mean RSA for each episode	Quantified using Porges method with MXedit Software (respiration bandwidth range of 0.24–1.04)	29% loss, reasons not provided	Edited and corrected by visual inspection using MXedit Software	Facial fear
Bush et al. (2017)^b^	**Distress/**still‐face Procedure/Sitting Quietly	4 electrodes/right clavicle, lower left rib, right abdomen	4/30 s	Mean RSA for each episode	Quantified using Porges method (respiration bandwidth range of 0.24–1.04)	50% loss due to artifacts and incomplete data	Data deleted if more than 25% of epoch was unscorable; Data cleaning procedures included checking all outliers (0.3 *SD*) by interval and summary scores using MXedit Software	Affect and gaze
Buss et al. (2004)^a^, ^b^	**Fear**/Stranger Approach/Video	NA/NA	NA/30 s	Mean HR and RSA for each episode	Quantified using Porges method with MXedit Software (respiration bandwidth range of 0.24–1.04)	25%–43% loss across tasks, reasons not provided	Less than 3% of data points edited and majority of 5‐min baseline signal and the 2 1⁄2‐min stranger signals preserved; Edited and corrected by visual inspection using MXedit Software	Facial fear
Buss et al. (2005)^a^, ^b^	**Fear and Distress**/Stranger Approach and Toy Removal/Video	4 electrodes/NA	1−3/20 s	Mean HP and RSA for each episode and/or task	Quantified using Porges method with MXedit Software (respiration bandwidth range of 0.24–1.04)	29%–37% loss across tasks due to equipment failure, sensor removal, and artifact	Edited data for 75% of sample; Edited and corrected by visual inspection using MXedit Software	Facial fear, and anger using AFFEX system; crying
Busuito and Moore (2017)^b^	**Distress**/Face‐to‐Face Still‐Face/Sitting Quietly	2 electrodes/chest	8/15 s	Mean RSA from each episode	Quantified using Porges method with MXedit Software (respiration bandwidth range of 0.24–1.04)	32%–36% loss across tasks due to artifacts, child distress, and equipment failure	Outliers replaced by dividing or summing them so they were consistent with adjacent data. Data files requiring editing of more than 2% or had a standard deviation across epochs greater than 1.00 excluded using MXedit Software	Affect and gaze
Busuito et al. (2019)^a^, ^b^	**Distress**/Face‐to‐Face Still‐Face/Sedentary Task	3 electrodes/chest	4/30 s	Mean HP and RSA for each episode	Quantified using Porges method with MXedit Software (respiration bandwidth range of 0.24–1.04)	4%–27% across tasks for HP and 4−32% for RSA; reasons not provided	Data were edited using the R‐peak Editor system, which uses an algorithm to systematically insert missed or correct outlier R‐peaks using MXedit Software	Affect and gaze
Calkins, 1997)^b^	**Positive and Frustration**/Puppet Play, Toy Removal in Box/Video	3 electrodes/inverted triangle on chest	8/15 s	Mean RSA for each task	Quantified using Porges method with MXedit Software (respiration bandwidth range not provided)	7%–16% loss across tasks due to refusal to participate, other reasons not provided	Data scanned for outlier points relative to adjacent data and replacing those points by dividing them or summing them; Files requiring editing of more than 2% of data were excluded; Typically, less than 1% of HR data required editing using MXedit Software	Duration and latency to smile, fuss, or frown; temperamental reactivity; ER strategies
Calkins and Fox (1992)	**Distress and Fear**/Arm Restrain, Strange Situation, Stranger Approach/Sitting Quietly	NA/NA	NA/NA	Mean HP and RSA for each episode and/or task	Quantified using Porges method (respiration bandwidth range not provided)	% data loss not reported	Edited and corrected by visual inspection using MXedit Software	Frequency of crying
Calkins and Dedmon (2000)^b^	**Positive, Fear, Frustration, and Distress/**Puppet Play, Spider, Food Denial, and Crying Audio/Video	3 electrodes/inverted triangle on chest	8/15 s	Mean HP and RSA for each task	Quantified using Porges method with MXedit Software (respiration bandwidth range of 0.24–1.04)	32% loss due to refusal to wear electrodes, equipment failure, and artifacts	Data scanned for outlier points relative to adjacent data and replacing those points by dividing them or summing them; Files requiring editing of more than 2% of data were excluded using MXedit Software	Verbal and facial affect and behavior
Calkins and Johnson (1998b)	**Frustration**/Plexiglass Barrier, Food Denial, High Chair, and Toy Removal/Video	3 electrodes/inverted triangle on chest	4/30 s	Mean HP and RSA for each task	Quantified using Porges method with MXedit Software (respiration bandwidth range not provided)	12% loss due to refusal of electrodes, equipment failure, and artifacts	Edited and corrected by visual inspection using MXedit Software	Latency to cry; intensity of distress; frequency of fussing; duration of crying
Calkins and Keane (2004)^a^, ^b^	**Frustration and Distress**/Crying Audio and Locked Box/Video	3 electrodes/inverted triangle on chest	4/30 s (audio) and 8/30 s (video)	Mean HP and RSA for each task	Quantified using Porges method with MXedit Software (respiration bandwidth range of 0.24–1.04)	8%–11% loss at 2 years and 3%–7% at 4 years due to refusal of electrodes, equipment failure, and artifacts	If *SD* across epochs greater than 1.00 for RSA, episode was excluded; Data scanned for outliers relative to adjacent data and replacing those points by dividing or summing them; Data requiring editing of more than 5% were excluded using MXedit Software	Verbal and physical frustration and global regulation
Calkins et al. (1998a)^b^	**Positive and Frustration**/Puppet Play and Toy Removal/Video	3 electrodes/inverted triangle on chest	NA/NA	Mean RSA for each task	Quantified using Porges method with MXedit Software (respiration bandwidth range not provided)	4%–20% loss across tasks due to artifacts or refusal of electrodes	Edited and corrected by visual inspection using MXedit Software	Duration of and latency to smile or fuss; facial and vocal affect
Campos et al. (1975)^a^	**Fear**/Multiple Stranger Approach/Sitting Quietly	NA/NA	5/every 3 s per phase	Mean HR for each episode	HR sampled every 3 s during each phase	19% loss due to equipment failure or experimental error	NA	Facial affect, gaze, motor activity, and global distress
Cho and Buss (2017)^b^	**Positive**/Puppet Play/Sedentary Task	NA/NA	NA/30 s	Mean RSA for task	Quantified using Porges method with Mindware software (respiration bandwidth range of 0.24–1.04)	45% loss due to invalid data	Visual inspection and editing of artifacts in data completed by three scorers who achieved good interrater reliability (agreement = 86%) on 25% of the files using Mindware Software	Duration and latency to freeze, duration of facial fear and bodily fear
Dawson et al. (2001)	**Positive**/Peek‐A‐Boo Play/Sedentary Task	2 electrodes/sternum and left costal region	NA/NA	Mean HR for task	HR samples, collected every 10 millisecond (ms), used to calculate mean HR	% data loss not reported	Output of the R‐wave detection was edited and corrected by visual inspection using MXedit Software	NA
Eiden et al. (2018)^c^	**Distress**/Arm Restraint/Video	3 electrodes/triangulated on chest; respiration bellows at bottom of the sternum	NA/NA	Mean RSA for task	Quantified using Grossman's method with IBI Analysis software (James Long Company)	17% loss, reasons not provided	Data files of R‐wave intervals were manually edited to remove incorrect detection of R‐wave or artifacts by blind assessor	Demandingness, affect, responsiveness to mother, and self‐reliance
Eisenberg et al. (2012)^c^	**Distress**/Videos—Crying/Video	2 electrodes/armpits at chest level and on back; Respiration bellows placed around abdominal area	NA/NA	Mean RSA for task	ECG data analyzed with IBI analysis software using peak‐to‐valley method; Baseline RSA was regressed onto Distressed RSA and multiplied by −1 to create measure of RSA suppression that is orthogonal to baseline RSA	14% loss due to child distress, equipment failure, and artifacts	Edited and corrected by manual visual inspection	NA
Feldman et al. (2010)^b^	**Distress**/still‐face, Face‐to‐Face/Play	NA/NA	NA/NA	Mean RSA for episodes	Quantified using Porges method with MXedit Software (respiration bandwidth range of 0.24–1.04)	% data loss not reported	Edited and corrected by visual inspection using MXedit Software	Gaze, affect, vocalizations, and touch
Fracasso et al. (1994)^a^, ^b^	**Positive and Negative**/Positive and Negative Stimuli/Sitting Quietly	NA/chest	40/30 s (7 and 10 months) and 60/30 s (13 months)	Mean HP and RSA for episodes	Quantified using Porges method with MXedit Software (respiration bandwidth range of 0.24–1.04)	21%–40%, reasons not provided	Edited and corrected by visual inspection using MXedit Software; aberrant values adjusted by integer addition and division of sequential R‐intervals; less than 1% of data adjusted because of artifacts	NA
Gilissen et al. (2007)	**Fear**/Video—Scary/Video	3 electrodes/inverted triangle on chest	1/60 s	Mean HRV for task	RMSSD computed from raw IBI data to index HRV; RMSSD sampled every 10 s	20% loss due to refusal to participate and equipment failure	Unacceptable physiological values (*z* > 3.29) were found and changed into the next most extreme score	Blocking and escape behaviors
Gilissen et al. (2008)	**Fear**/Video—Scary/Video	3 electrodes/inverted triangle on chest	1/60 s	Mean HRV for task	RMSSD measured as index of HRV	11% loss due to equipment failures and outliers	Missing and unacceptable values replaced with mean HRV values for subgroup matched for child gender and age.	NA
Gray et al. (2017)^b^	**Distress**/still‐face Paradigm/Play	NA/NA	NA/NA	Mean RSA for episodes	IBIs detrended using moving polynomial filter to remove slow trends; RSA estimated from power spectral analysis using discrete Fourier transform and quantified in range for 4‐month‐old infants (0.30–0.75 Hz); RSA values were winsorized to 3 standard deviations from mean and natural log transformed	17% loss due to artifacts, equipment failure, human error, or incomplete data	NA	NA
Haley and Stansbury (2003)^a^	**Distress**/still‐face Paradigm/Play	2 electrodes/chest	1/120 s	Mean HR for episodes	NA	12% loss due to distress or data loss	Data files digitally filtered and reviewed for artifacts; filtered data replaced with mean values of the surrounding intervals; no more than 2%–3% of any file required filtering	Gaze, affect, fussing, and crying
Ham & Tronick (2006)	**Distress**/still‐face Paradigm/Play	NA/NA	1/120 s	Mean HR and RSA for episodes	Pilot study; details not provided	25% due to equipment failure	NA	Used Infant Caregiver Engagement Phases, codes affect, gaze, and vocalizations
Ham & Tronick (2009)^a^, ^b^	**Distress**/still‐face Paradigm/Play	3 electrodes/shoulders and side	1/120 s	Mean HR and RSA for episodes	Quantified using algorithm with filter for infant respiration (respiration bandwidth range of 0.24–1.04) in Chart software	6%–28% loss across tasks due to equipment failure or noncompliance	NA	Used Infant Caregiver Engagement Phases, codes affect, gaze, and vocalizations
Hay et al. (2017)^a^	**Distress**/Teddy Bear Picnic/Unknown Task	NA/upper left leg	NA/15 s	Mean HR for episodes	HR sampled at 30 HZ and represented as bpm	7% loss due to incorrect procedure, wrong sensors, refusal to wear electrodes, and equipment failure	Logarithmic transformation applied to HR data to improve normality of distribution	Used Distress Observation System (DOS) to code vocal distress
Hill‐Soderlund et al. (2008)^b^	**Fear**/Ainsworth Strange Situation/Play	2 electrodes/chest	4/15−30 s	Mean RSA for episodes	Quantified using Porges method (respiration bandwidth range of 0.24–1.04)	46%–62%% loss across tasks due to equipment failure and artifacts; Excluded only if they were completely missing all physiological data.	Data files that required editing of more than 10% of the data were not included in the analyses and were considered missing.	NA
Holochwost et al. (2014)^b^	**Distress**/still‐face Paradigm/Sitting Quietly	2 electrodes/chest	8/15 s	Mean RSA for episodes	Quantified using Porges method with MXedit software (respiration bandwidth range of 0.24–1.04)	12%–23% loss across tasks, reasons not provided	Edited for artifacts using MXEdit software; Data files requiring editing of more than 10% were excluded	NA
Johnson et al. (2014)^b^	**Distress**/Arm Restraint, still‐face Paradigm/Sitting Quietly	3 electrodes/forehead and chest	8/15 s	Mean RSA for episodes	Computed using Fast Fourier transformation with Mindware software (respiration bandwidth range of 0.24 to 0.40)	24% loss due to artifacts, electrode placement difficulties, and equipment failure	Correction of artifact, motion, and error in automated marking of R‐waves was performed manually;	Facial affect coded using Mangold INTERACT software
Liew et al. (2011)^c^	**Distress**/Videos—Crying/Video	3 electrodes/lower ribs and back; respiration cord around abdominal area	1/42 s	Mean RSA for task	“Peak‐to‐valley” metric using James Long software; RSA data multiplied by constant value of 10 for analyses. RSA suppression indexed by reversed score of standardized residualized RSA change score (calculated by computing regression with RSA during neutral film as predictor and RSA during emotion film as outcome and multiplying value by − 1)	13% loss at 18 months and 10% at 30 months, reasons not provided	For baseline, scores three SDs above/below mean coded as missing so values were estimated in *SEM* or multiple imputations procedures.	NA
Mireault et al. (2018)^a^	**Positive**/Absurd Event/Play	3 electrodes/inverted triangle on chest	1/45 s	Mean HR for episodes	HR in beats per minute	50% loss across sample, reasons not provided; included only children with complete data	NA	Duration of smiling/laughing and gaze
Moore (2009)	**Positive, Angry, Neutral, and Distress**/Emotion‐Evoking Task and still‐face paradigm/Sitting Quietly	NA/chest	8/15 s	Mean RSA for episodes and/or task	Quantified using Porges method with MXedit software (respiration bandwidth range not provided)	44% loss due to distress, equipment failure, and artifacts	Data scanned for outliers; editing of more than 2% or RSA standard deviation across epochs greater than 1.00 were excluded using MXedit Software	Affect and gaze
Moore and Calkins (2004)^a^, ^b^	**Distress**/still‐face Paradigm/Sitting Quietly	3 electrodes/inverted triangle on chest	8/15 s	Mean HR and RSA for episodes	Quantified using Porges method with MXedit software (respiration bandwidth range of 0.24 to 1.04)	14%–17% loss due to equipment failure and movement artifacts	Data scanned for outliers relative to adjacent data and replacing those points by dividing them or summing them; Data files requiring editing of more than 2% of the data were excluded using MXedit software	Affect and gaze
Moore et al. (2009)^b^	**Distress**/still‐face Paradigm/Sitting Quietly	NA/chest	8/15 s	Mean HP and RSA for episodes	Quantified using Porges method with MXedit software (respiration bandwidth range not provided)	37%–45% loss across tasks due to equipment failure, artifacts, distress, falling asleep	Data scanned for outliers using MXedit Software	Affect and gaze
Morasch and Bell (2012)	**Distress**/Arm Restraint/Video	3 electrodes/right collarbone, lower left rib, ground at scalp	NA/NA	Mean HR and HRV for task	Mean heart rate in beats per minute and heart rate variability	19% loss at 5 months and 9% at 10 months due to artifacts, equipment failure, and electrode misplacement	Artifact scored by trained research assistant for movements using IBI Analysis software developed by James Long Company	Gaze and affect
Noten et al. (2019a)^a^	**Positive, Sadness, and Fear**/Video clips/Video clip	3 electrodes/right collarbone, left apex of heart, and right side between lower two ribs	1/50 s	HR	Mean heart rate in beats per minute	11% loss due to refusal to wear electrodes or loose electrodes	Data were visually checked by trained researcher and adjusted manually	NA
Noten et al. (2019b)^b^	**Distress and Frustration**/Still‐Face Paradigm and Car Seat Task/Video	3 electrodes/right collarbone, left apex of heart, and right side between lower two ribs	1/120 s for Still‐Face and 1/60 s for Car Seat	RSA	Quantified using Grossman's method with IBI Analysis software (VU‐DAMS software)	8% loss for still‐face paradigm and 15% loss for car seat task due to artifacts, technical problems, and child refusal	Data were visually checked by trained researcher and adjusted manually	Vocalizations, self‐soothing, and escape behaviors
Paret et al. (2015)^a^, ^b^	**Fear**/Interesting but Scary/Video	3 electrodes/inverted triangle on chest	8/15 s	Mean HR and RSA for tasks	RMSSD is sensitive to fluctuations in IBI in respiratory range, representing a high‐pass filter that captured high‐frequency RSA; its natural logarithm (lnRMSSD) used in analyses	45% loss due to equipment failure, artifacts, and child refusal	Waveforms examined for artifacts that interfere with accurate extraction of IBI length; edited by replacement with nonvoltage peak values; Epochs requiring more than 10% editing or with standard deviation greater than 1.00 were excluded	Nonverbal anxiety and verbal responsiveness
Perry et al. (2012)^b^	**Frustration**/Frustrating Puzzle Task/Video	2 electrodes/chest and stomach	12/15 s	Mean RSA for task	Quantified using Porges method with MXedit software (respiration bandwidth range of 0.24 to 1.04)	19% loss due to refusal to wear electrodes, artifacts	Editing consisted of examining outliers and dividing or summing; Data files requiring editing of more than 10% of the data were excluded using MXedit software	Verbal and physical distractions
Perry et al. (2016)^b^	**Distress**/Arm Restraint/Sedentary Task	2 electrodes/right collarbone and lower left rib	NA/NA	Mean RSA for task	Spectral analysis used to calculate RSA using discrete Fourier transform with frequency band for quantification of 0.24–1.04 Hz. The RSA data were transformed using natural log to normalize the distribution.	47% loss due to artifacts, equipment failure, and incomplete data	ECG signal visually inspected for software‐detected R‐waves; Movement artifact was designated by absence of at least three consecutive R‐waves. These epochs were excluded	Mother orientation and distraction
Pratt et al. (2015)^b^	**Distress**/still‐face with Touch or Arm Restraint/Play	3 electrodes/NA	8−12/15 s	Mean RSA for episode	Quantified using Porges method with MXedit software (respiration bandwidth range of 0.24 to 1.04)	16% due to equipment failure, and child discontinuation	Data scanned for outliers using MXedit Software	Gaze, affect, vocalizations, touch, autonomic response, motor response, and self‐soothing behavior
Provenzi et al. (2015)	**Distress**/Face‐to‐Face Still‐Face/Play	3 electrodes/inverted triangle on chest	12/10 s	Mean RSA for episode	Quantified using Porges method with MXedit software (respiration bandwidth range of 0.24 to 1.04)	41% due to equipment failure, missing data, and artifacts	IBI data screened offline to correct potential errors in automatic detection of R‐wake peaks	Negative, positive, and object/environment engagement, and social monitoring
Provost and Gouin‐Decarie (1979)^a^	**Fear and Frustration**/Stranger Situation with Locked Box/Before	2 electrodes/left nipple and left shoulder blade in lining of jacket	36/5 s	Mean HR for episode and/or task	Mean heart rate in beats per minute	35% loss due to child distress and unusable recordings	NA	Motor, hedonic tone, affect, and vocalizations
Qu and Leerkes (2018)^b^	**Distress**/Face‐to‐Face Still‐Face /Play	3 electrodes/collarbone and lower ribs	8/15 s epochs	Mean RSA for episode	Quantified using Porges method (respiration bandwidth range not provided)	20% loss due to artifacts	NA	Affect coded using INTERACT
Rash et al. (2015)^c^	**Distress and Frustration**/Toy Retraction, Plexiglass Barrier, Arm Restraint/Sitting Quietly	2 electrodes/clavicle and ribcage	3/45 s	Mean RSA for task	Quantified as average power spectral density of R‐R fluctuations occurring in respiratory band (0.24—1.04 Hz) using Grossman method	28% loss due to acquisition error, unusable data, incomplete recordings, artifacts, and infant fussing	Outliers from previous and subsequent 50 IBIs by a value greater than 20% were interpolated; No participant in final sample required more than 5% data interpolation	NA
Rash et al. (2016)^c^	**Distress and Frustration**/Toy Retraction, Plexiglass Barrier, Arm Restraint/Sitting Quietly	2 electrodes/clavicle and ribcage	3/45 s	Mean RSA for task	Quantified using power spectral density of R‐R fluctuations during respiration (bandwidth range of 0.24–1.04)	17% loss due to equipment failure, artifacts, missing data, and outliers	Outliers identified as values exceeding z‐score of 3.29 and adjusted according to recommendations; No more than four values (1.5%) were adjusted for any variable	NA
Santesso et al. (2007)^a^	**Positive and Fear**/Audio—ID speech/Unknown Task	2 electrodes/chest	1/60 s	Mean HP for task	HR bandpass filtered between 1–100 Hz and sampled at 512 Hz	% loss and reasons not provided	All data were visually inspected for artifact identification and edited by a second team of coders.	NA
Schmidt et al. (2003)^a^	**Positive, Fear, and Sadness**/Musical Pieces/Unknown Task	2 electrodes/chest	1/30 s	Mean HP for task	HR bandpass filtered between 1–100 Hz and sampled at 512 Hz	2% at 9 months and 38% at 12 months due to equipment failure	All data were visually inspected for artifact identification and edited by a second team of coders.	NA
Scrimgeour et al. (2016)	**Disappointment**/Disappointment Task/Sedentary Task	3 electrodes/torso	1/30 Seconds	Mean RSA for task	RSA defined as natural log integral of 0.24–1.04 Hz power band using Mindware HRV	56% had incomplete data (but were imputed in SPSS); reasons not provided	All data were visually inspected for artifact identification and edited by a second team of coders.	NA
Skarin (1977)^a^	**Fear**/Stranger Approach/Before	3 electrodes/breasts and ankle	NA/0.5 s	Mean HR for task	Each 5 s segment of HR was subtracted from baseline HR in each segment of approach	3% loss due to equipment failure	NA	Facial expressions
Spangler and Grossmann (1993)^a^	**Fear/**Ainsworth Strange Situation/Play	3 electrodes/inverted triangle on chest	NA/NA	Mean HR for episodes	HP converted to HR in beats per minute for each 1‐s interval	26% loss due to nonvalid HR scores	HP were controlled for by artifacts by computer using Foerster (1984) criteria	Facial emotion and orientation to mother or objects
Stone and Porter (2013)	**Distress**/Arm Restraint and still‐face‐Face‐to‐Face/Unknown Task	NA/NA	NA/NA	Mean HP and RSA for episodes and/or task	Quantified using Porges method with MXedit software (respiration bandwidth range not provided)	23% loss due to incomplete data	MXedit software used to edit outliers due to movement and recording artifacts	NA
Vaughn and Sroufe (1979)^a^	**Positive and Fear**/Peek‐a‐Boo then Mask/Sitting Quietly	NA/sternum	NA/NA	Mean HR and time to crying for task	Nothing reported on HR	Only included infants with complete records, original sample not reported	NA	First cried and facial expression changed to cry face
Wagner et al. (2018)a^b^	**Fear**/Stranger Challenge/Video	3 electrodes/chest	NA/NA	Mean RSA for task	MxEdit computed RSA in 0.24–1.04 Hz frequency bandwidth	4% loss for baseline and 10% for stranger procedure due to unusable cardiac data	Recording errors in IBIs edited using MxEdit software	NA
Wagner et al. (2018b)^b^	**Anger**/Narrated Comic Strip/Video	3 electrodes/chest	1/60 s	Mean RSA for task	MxEdit computed RSA in 0.24–1.04 Hz frequency bandwidth	5% loss, reasons not provided	Recording errors in IBIs edited using MxEdit software	NA
Waters et al. (1975)	**Fear**/Stranger Wariness/Before	NA/NA	NA/NA	Mean HR and time to peak HR	NA	% data loss not reported; removed participants who smiled or cried from analyses	NA	Positive, neutral, and negative responses
Weinberg and Tronick (1996)^a^, ^b^	**Distress/**Face‐to‐Face still‐face/Play	NA/NA	12/10 s	Mean HR and RSA for episodes	Analyzed with MXEdit software (respiration bandwidth range not reported	% data loss not reported	Recording errors in IBIs edited using MxEdit software	Affect coded using AFFEX system and behavior coded using Infant Regulatory Scoring System
Zeegers et al. (2017)^c^	**Fear**/Stranger Approach/Unknown Task	2 electrodes/collarbone and rib	1/30 s	Mean HRV for task	RMSSD of successive normal‐to‐normal intervals	17% loss at 4 months and 27% at 12 months for Baseline; 28% loss at 4 months and 38% at 12 months for tasks due to families not visiting laboratory, artifacts, and equipment failure	R‐waves were identified and adjusted for artifacts.	NA
Zeytinoglu et al. (2019)^b^	**Frustration**/Locked Box Task and Toy Removal/Videos	2 electrodes/collarbone, lower left rib, and lower right rib	8/30 s for lock box and 4/30 s for toy removal	Mean RSA	Computed using Fast Fourier transformation with Mindware software (respiration bandwidth range of 0.24 to 1.04)	11% loss in locked box and 13% in toy removal due to artifacts, equipment failure, and electrode placement errors	All data were visually inspected for artifact identification and edited by trained researchers.	Overall composite of global regulation, verbal negativity, and latency to distress

^a^ Included in HR meta‐analysis.

^b^ Included in RSA meta‐analysis.

^c^ Included in HRV meta‐analysis.

**Table 4:** Description of physiological results

Measure	Article	Sample Size/Age	Baseline Mean (*SD*)	Task Mean (*SD*) and Effect Size (Cohen's *d*)	Significant Differences (Condition, Age, and Sex)	Significant Relationship(s) with Behavior
*Heart Rate (HR)*						
HR	Anderson et al. (1999)^*^	**45**/5 and 10 months	NA	5 months: −4.1 bpm; 10 months + 1.5 bpm from baseline; all ds = NA	Condition x Age Effect (↓ HR at 5 months and ↑ HR at 10 months from baseline); Did not test for Sex Effect	↑ wariness = ↑ HR
HR	Baker et al. (2012)^*^	**70, 64, 61**/12, 24, and 36 months	Year 1:136.43 (12.15) bpm; Year 2:121.86(9.74) bpm; Year 3:113.72 (9.84) bpm	Year 1:141.34 (15.78) bpm, *d* = 0.35; Year 2:133.11 (13.97) bpm, *d* = 0.94; Year 3:117.72 (14.77) bpm, *d* = 0.32	Age (HR ↓ with age) and Condition Effect; No Sex Effect	None reported
HR	Bazhenova et al. (2007)^*,^^	**16/**3–4 months	155 (7.75) bpm	148 (4.69) bpm, *d* = 1.13	Condition Effect; Did not test for Sex Effect	None reported
HR	Bohlin and Hagekull (1993)^*^	**31**/10–13 months	Mpres: 130.2 bpm; Mabs: 146.8 bpm	Distance Phases Mpres = 130.3 bpm, *d* = NA and Mabs = 147.7 bpm; *d* = 0.98; Intrusion Phases were Mpres = 136.4 bpm, *d* = NA and Mabs = 150.9 bpm, *d* = 1.02	Condition Effect (Intrusion versus Baseline); Did not test for Sex Effect	↑ wariness = ↑ HR
HR	Buss et al. (2004)^*,^^	**46**/24 months	119.03 (8.53) bpm	122.98 (9.27) bpm, *d* = 0.45	Did not test for Condition or Sex Effect	↑ Freeze composite = ↓HR
HR	Buss et al. (2005)^*,^^	**68/24 months**	119.03 (8.53) bpm	Stranger: 122.75 (9.27) bpm, *d* = 0.42; Toy Removal: 126.98 (14.46) bpm, *d* = 0.68	Condition Effect (Toy Removal versus. Stranger and Baseline; Stranger versus Baseline;) No Sex Effect	↑ negative affect = ↑ HR in Stranger Approach and Toy Removal
HR	Busuito et al. (2019)^*,^^	**116/**6 months	138 (9.79) bpm	Play: 137 (10.72) bpm, *d* = 0.10; SF: 145 (12.49) bpm, *d* = 0.63; Reunion: 144 (13.14) bpm, *d* = 0.52	Condition Effect (SF versus Play); No Sex Effect	↑ positive and negative affect = ↓HR in play and reunion; ↑ negative affect = ↓ HR in SF
HR	Calkins and Fox (1992)	**48−52**/5, 14, and 24 months	NA	NA; *d* = NA	Did not test for Condition or Sex Effect	None reported
HR	Calkins and Keane (2004)^*,^^	**135 and 115/**2 and 4.5 years	Age 2:109 (10.89) bpm; Age 4.5:97 (12.86) bpm	Age 2‐Empathy: 113 (10.16) bpm, *d* = 0.38; Frustration: 116 (10.91) bpm, *d* = 0.64; Age 4.5‐Empathy: 98 (12.11) bpm, *d* = 0.08; Frustration: 101 (11.24) bpm, *d* = 0.33	Did not compare HR by Condition; Did not test for Sex Effect	None reported
HR	Calkins and Johnson (1998b)	**73**/18 months	NA	147 bpm; *d* = NA	Did not test for Condition Effect; No Effect of Sex	None reported
HR	Campos et al. (1975)^*^	**80**/5 and 9 months	NA	NA; all ds = NA	Age Effect (9 months ↑ HR versus. 5 months ↓HR during Task); No Sex Effect	↑ negative affect = ↑ HR
HR	Dawson et al. (2001)	**61/**13−15 months	128 bpm	Mother Play: 131 bpm, *d* = 0.44; Experimenter play: 126 bpm, *d* = NA	Condition Effect (Both tasks versus Baseline); Did not test for Sex Effect	NA
HR	Fracasso et al. (1994)^*,^^	**44−58**/5, 7, 10, and 13 months	5 months: 142 (9.76) bpm; 7 months: 138 (9.90) bpm; 10 months: 135 (10.30) bpm; 13 months: 136 (10.80) bpm	7 months: 144 (10.05) bpm, *d* = 0.61; 10 months: 137 (9.89) bpm, *d* = 0.20; 13 months: 139 (10.92) bpm, *d* = 0.28	Age and Condition effects; Did not test for Sex Effect	NA
HR	Haley and Stansbury (2003)^*^	**43**/3–4 months	Play 1:146.60(11.92) bpm	SF1: 148.79 (11.60) bpm, *d* = 0.19; Reunion 1:146.55 (12.07) bpm, *d* = 0.004; SF2: 151.15 (12.21) bpm, *d* = 0.38; Reunion 2:149.11(13.15) bpm, *d* = 0.20	Condition Effect (SF1 vs. Play1 and Reunion; SF2 vs. Reunion 1); Sex Effect (Boys had ↓ HR than girls)	↑ negative affect = ↑ HR in SF II
HR	Ham and Tronick (2006)	**12/5 months**	FF: Recovered: 145 bpm Stable: 142 bpm; Dysregulated: 137 bpm; Protest: 152 bpm	SF‐Recovered: 145 bpm; Stable: 145 bpm; Dysregulated: 155 bpm; Protest: 153 bpm; Reunion‐Recovered: 150 bpm; Stable: 145 bpm; Dysregulated: 170 bpm; Protest: 160 bpm; all ds = NA	Descriptive; Did not test for Condition or Sex effect	↑ protest = ↑ HR in all SF episodes
HR	Ham and Tronick (2009)^*,^^	**18/5 months**	143.57 (9.97) bpm	151.67 (11.93) bpm, *d* = 0.76	Condition Effect (SF versus FF;) Did not test for Sex Effect	↑ mother engagement = ↓ HR; ↑ negative affect = ↑ HR in SF and Reunion; ↑ social engagement = ↑ HR overall
HR	Hay et al. (2017)^*^	**255**/12 months	132.81 (21.04) bpm	141.16 (15.21) bpm, *d* = 0.46	Sex Effect (Boys had ↓ HR during task)	↑ vocal distress = ↑ HR
HR	Mireault et al. (2018)^*^	**34, 17**/4, 6 months	139.4 (12.43) bpm	4 months: Absurd Neutral: 136.1 (10.85) bpm, *d* = 0.29; Absurd Cued: 138.9 (11.62) bpm, *d* = 0.04; 4 Months: HR Absurd Neutral 136.5 (10.65) bpm, *d* = 0.25; 6 months: 130.7 (11.07) bpm, *d* = 0.74	Condition Effect (Absurd Cued versus Play, Absurd Ordinary at 4 and 6 months), Age Effect (4 mos had ↑ HR than 6 mos for Absurd Neutral); Did not test for Sex Effect	↓ gaze to parent = ↑ HR at 4 months; ↑ gaze to parent = ↑ HR at 6 months
HR	Moore and Calkins (2004)^*,^^	**60**/3 months	146 (10.89) bpm	Play: 147(10.33) bpm, *d* = 0.010; SF: 152 (12.19) bpm, *d* = 0.52; Reunion: 148 (11.90) bpm, *d* = 0.18	Condition Effect (Baseline versus SF and Reunion; SF versus Play and Reunion; Reunion versus Play; Change SF versus Play and Reunion; Reunion versus Play)	↑ negative affect = ↑ HR during in Reunion; ↑ negative affect = ↑ change HR in SF and Reunion
HR	Morasch and Bell (2012)	**75 and 84/5 and 10 months**	NA	5 months: NA; 10 months: NA; *d* = NA	Condition Effect (for HR, not HRV at 5 mos and both at 10 mos); No Effect of Sex; Did not test for Age Effect	↑ negative affect = ↑ HR in postdistress phase at 5 and 10 months; ↑ negative affect = ↑ HRV and ↓ gaze duration = ↑ HR in postdistress phase at 10 months
HR	Noten et al. (2019a)^*^	**54/45 months**	Happy: 107.62 (11.08) bpm; Sad: 105.49 (10.48) bpm; Fear: 106.25 (11.17) bpm	Happy: 105.35 (10.87) bpm, *d* = 0.21; Sad: 101.49 (11.31) bpm, *d* = 0.36; Fear: 103.22 (11.47) bpm, *d* = 0.27	Condition Effect (Each video versus baseline); No Effect of Sex	NA
HR	Paret et al. (2015)^*,^^	**48**/44 months	103.47 (10.21) bpm	111.55 (11.00) bpm, *d* = 0.77	Condition Effect (↑ HR for IbS ); No Effect of Sex	None reported
HR	Provost and Gouin‐Decarie (1979)^*^	**26/9–12 months**	With Mother: 147.2 bpm; Frustration Baseline: 148.5 bpm; Isolation Baseline: 147.7 bpm; Reunion Baseline: 156.4 bpm	Play with Mother: 144.2 bpm, *d* = 0.28; Frustration: 156.5 bpm, *d* = 0.87; Isolation: 162.5 bpm, *d* = 0.70; Reunion: 148.0 bpm, *d* = 0.38	Condition Effect (↑ HR in anger and distress tasks); Did not test for Sex Effect	None reported
HR	Santesso et al. (2007)^*^	**34/**9 months	138 bpm	Comfort: 134 bpm, *d* = 0.97; Surprise: 135 bpm, *d* = 0.88; Fear: 134 bpm, *d* = 0.69	Condition Effect (↓ HR in task); Did not test for Sex Effect	NA
HR	Schmidt et al. (2003)^*^	**33−52/**3, 6, 9, and 12 months	3 months: 147 (15.53) bpm; 6 months: 144 (11.74) bpm; 9 months: 136 (11.42) bpm; 12 months: 131 (11.56) bpm	3 months: 142(14.13) bpm, *d* = 0.34; 6 months: 139 (10.48) bpm, *d* = 0.46; 9 months: 139 (11.54) bpm, *d* = 0.26; 12 months: 130 (11.74) bpm, *d* = 0.09	Condition Effect (Music versus Baseline at 3, 6, and 9 months); Did not test for Age or Sex Effect	NA
HR	Skarin (1977)^*^	**32**/5–7, 10–12 months	NA	NA; *d* = 0.83	Condition Effect (HR↑ with task); Age Effect (HR↑ with age); No Effect of Sex	None reported
HR	Spangler and Grossmann (1993)^*^	**30**/12 months	Insecure: 142.3 (18.2) bmp; Disorganized: 134.2 (6.9) bpm; Secure: 140.7(12.0) bpm	Infant Alone 1 Insecure: 148.5 (17) bpm, *d* = 0.36; Disorganized: 139.8 (22) bpm, *d* = 0.35; Secure: 149.6 (11.8) bpm, *d* = 0.76; Infant and Stanger 2 Insecure: 139.9(17.6) bpm, *d* = 0.14; Disorganized: 150 (22.8) bpm, *d* = 0.95; Secure: 147.2 (19.6) bpm, *d* = 0.41	Condition Effect (Infant Alone versus Baseline); Did not test for Sex Effect	None reported
HR	Stone and Porter (2013)	**78**/6 months	Bradycardia: 154 (12.08) bpm; NonBradycardia: 151 (11.88) bpm	Bradycardia: 153 (11.94) bpm, *d* = 0.08; NonBradycardia: 152 (20.97) bpm, *d* = 0.06	Did not test for Condition Effect; No Effect of Sex	NA
HR	Vaughn and Sroufe (1979)^*^	**16**/8–16 months	130 bpm	NA, *d* = 1.08	Condition (↑ HR prior to crying onset)	Each child showed a “cry face” prior to crying
HR	Waters et al. (1975)	**26**/5 and 7 months	120 bpm	Wary versus NonWary Peak: 5 months: + 4 bpm for both; 7 months: + 12 bpm versus + 4 bpm; 9 months: +10 bmp versus + 4 bpm; Peak HR Pick‐Up 5 months: +16 bmp versus + 6 bpm; 7 months: +10 bpm versus + 6 bpm; 9 months: +14 bpm versus + 4 bpm; all ds = NA	Did not test for Condition, Age, or Sex Effect	↑ gaze aversion just before ↑ peak HR acceleration
HR	Weinberg and Tronick (1996)^*,^^	**45**/6 months	138.20 bpm	SF: 143.88 bpm; Reunion: 139.91 bpm; *d* = 0.73	Condition Effect (↓HR in SF); Did not test for Sex Effect	None reported
*Heart Rate Variability (HRV)*						
HRV^$^	Eiden et al. (2018)^#^	**57/9 months**	Boys: 0.016 (0.01) sec; Girls: 0.02 (0.01) sec	Boys change: −0.003 (0.006) sec, *d* = 0.35; Girls change: −0.004 (0.01) sec, *d* = 0.40	Did not test for Condition Effect; No Effect of Sex	↑ regulation = ↓ RSA suppression
HRV^$^	Eisenberg et al. (2012)^#^	**202/18 months**	0.02 (0.01) sec	Distress: 0.03 (0.02) sec, *d* = 0.5; Suppression: 0.00 (0.01), *d* = 0.00	No Effects of Age or Sex	NA
HRV^$^	Gilissen et al. (2007)	**78/44 months**	Alone: 0.66 (12.6); With Parent: 0.50 (11.12); [units unknown]	Fearful alone: −5.06 (11.55), *d* = 0.63; Fearful with parent: −4.21 (13.63), *d* = 0.30 [units unknown]	Condition Effect (no effect of Parent presence); Did not test for Sex Effect	None reported
HRV^$^	Gilissen et al. (2008)	**78/44 months**	0.66 (12.69) [units unknown]	Fear: −5.06 (11.55), *d* = 0.63 [units unknown]	Condition Effect; Did not test for Sex Effect	NA
HRV^$^	Liew et al. (2011)^#^	**247/18 and 30 months**	Residuals 18 months: 0.19 (0.11); 30 months: 0.30 (0.17) [units unknown]	Suppression 18 months: 0.00 (0.07), *d* = 0.00; 30 months: 0.00 (0.07), *d* = 0.00 [units unknown]	Did not test for Condition Effect; No Effect of Sex; Age Effect (Baseline 18 months vs. 30 months)	NA
HRV^$^	Rash et al. (2015)^#^	**194/6 months**	461.93 (425.52) ms2/Hz	396.92 (320.24) ms2/Hz, *d* = 0.16	Condition Effect (↓ RSA in task); No Effect of Sex	NA
HRV^$^	Rash et al. (2016)^#^	**254/6 months**	495.31 (402.72) ms2/Hz	Change = −55.14 (387.70), *d* = 0.14	Condition Effect (↓ RSA in task); No Effect of Sex	NA
HRV^$^	Zeegers et al. (2017)^#^	**84, 97/4, 12 months**	4 months: 16.57 (9.85) msec; 12 months: 29.88 (16.75) msec	Decline 4 months: −1.16 (10.01) msec, *d* = 0.12; 12 months: −1.92 (15.30) msec, *d* = 0.12	Did not test for Condition, Age or Sex Effect	NA
*Respiratory Sinus Arrhythmia (RSA)*						
RSA	Bazhenova et al. (2007)^*,^^	**16/3–4 months**	3.2 (0.3) In(msec2)	2.8 (0.3) In(msec2), *d* = 1.38	Condition Effect	None reported
RSA	Blankson et al. (2012)^^^	**263/42 months**	6.41 (1.32) In(msec2)	Locked Box RSA withdrawal 1.25 (0.84) In(msec2), *d* = 1.09; Green circle RSA withdrawal 0.67 (0.69) In(msec2), *d* = 0.64	Did not test for Condition or Sex Effect	None reported
RSA	Brooker et al. (2013)^^^	**88/6 months**	3.55 (0.77) In(msec2)	Suppression = 0.01 (0.96) In(msec2), *d* = 0.01	Did not test for Condition or Sex Effect	↑ stranger fear = ↓ RSA at baseline
RSA	Bush et al. (2017)^^^	**135/6 months**	Play 1 = 4.27 (1.04) In(msec2)	SF1 = 3.99(1.21) In(msec2), *d* = 0.18; Reunion 1 = 4.21 (1.21) In(msec2), *d* = 0.05; SF2 = 3.73 (1.16) In(msec2), *d* = 0.49; Reunion 2 = 4.10 (1.15) In(msec2), *d* = 0.16; Last SF (1 or 2) = 3.69 (1.27) In(msec2), *d* = 0.50	Condition Effect (SF 1 and 2 versus Play); Did not test for Sex Effect	None reported
RSA	Buss et al. (2004)^*,^^	**46**/24 months	5.06 (0.95) In(msec2)	4.71 (1.05) In(msec2), *d* = 0.35	Did not test for Condition or Sex Effect	None reported
RSA	Buss et al. (2005)^*,^^	**46/24 months**	5.06 (0.95) In(msec2)	Stranger: 4.71 (1.05) In(msec2), *d* = 0.35; Toy Removal: 4.48 (1.37) In(msec2), *d* = 0.50	Condition Effect (Stranger and Toy Removal versus Baseline); No Sex Effect	None reported
RSA	Busuito and Moore (2017)^^^	**50/6 months**	3.65 (1.07) In(msec2)	SF = 3.40 (0.93) In(msec2), *d* = 0.25; Reunion = 3.61 (1.09) In(msec2), *d* = 0.04	Condition Effect (Play versus SF); Sex Effect (Boys ↑ RSA at Baseline)	None reported
RSA	Busuito et al. (2019)^*,^^	**109/6 months**	3.18 (0.85) In(msec2)	SF: 3.85, (1.13) In(msec2), *d* = 0.59; Reunion: 3.79 (1.19) In(msec2), *d* = 0.59	No Effect of Task (by episode); Sex Effect (Boys ↑ RSA at Baseline)	↑ positive affect = ↑ RSA in play
RSA	Calkins (1997)^^^	**37/24–36 months**	5.66 In(msec2)	RSA Positive: 5.4 In(msec2), *d* = 0.48; Negative: 5.27 In(msec2), *d* = 0.7; RSA difference Positive: 0.30 (range 2.56 to 2.42), Negative: 0.33 (range 1.25 to 1.97);	Condition Effect (all tasks vs. Baseline); No Effect of Age or Sex	↑ positive and negative reactivity = ↑ RSA in baseline
RSA	Calkins and Fox (1992)	**48−52**/5, 14, and 24 months	NA	NA; *d* = NA	Did not test for Condition or Sex Effect	None reported
RSA	Calkins and Dedmon (2000)^^^	**85/24 months**	5.75 (11.41) In(msec2)	Puppets: 5.17 (1.24) In(msec2), *d* = 0.07; Spider: 4.94 (1.15) In(msec2), *d* = 0.10; Audio: 5.25 (1.28) In(msec2), *d* = 0.06; Food Denial: 5.00 (1.25) In(msec2), *d* = 0.09	Condition Effect (all tasks versus Baseline); Sex Effect for Baseline (Boys ↑ RSA than girls)	None reported
RSA	Calkins and Keane (2004)^*,^^	**135 and 115/24 and 53 months**	Age 2:5.76 (1.4) In(msec2); Age 4.5:5.95 (1.35) In(msec2);	Age 2: Empathy: 5.26 (1.25) In(msec2), *d* = 0.38; Frustration: 5.02 (1.24) In(msec2), *d* = 0.56; Age 4.5: Empathy: 6.00 (1.39) In(msec2), *d* = 0.04; Frustration: 5.65 (1.34) In(msec2), *d* = 0.22	Condition Effect (Baseline versus Empathy and Frustration at Age 2 and versus Frustration at age 4.5); Age Effect; Did not test for Sex Effect	None reported
RSA	Calkins and Johnson (1998b)	**73/18 months**	NA	4.05 In(msec2), *d* = NA	Did not test for Condition Effect; No Effect of Sex	↑ distress = ↑ RSA in barrier task
RSA	Calkins et al. (1998a)^^^	**52/18 months**	5.36 (1.22) In(msec2)	Positive: 4.98 (1.19) In(msec2), *d* = 0.32; Negative: 4.60 (1.13) In(msec2), *d* = 0.65; Suppression‐positive: 0.33 (0.77), *d* = NA; negative = 0.72 (0.71), *d* = NA	Did not test for Condition Effect; no Effect of Sex	↑ distraction and ↓ aggression = ↑ RSA suppression in Barrier task
RSA	Cho and Buss (2017)^^^	**62/24 months**	4.46(1.07) In(msec2)	Change: 0.00 (0.99), *d* = 0.00	Did not test for Condition Effect; No Effect of Sex	↓ fear = ↑ RSA suppression
RSA	Feldman et al. (2010)^^^	**53/6 months**	Touch: 3.56 (0.85) In(msec2); No Touch 3.65 (0.74) In(msec2)	Touch SF: 3.37 (0.56) In(msec2), *d* = 0.27; Reunion: 3.51 (0.87) In(msec2), *d* = 0.06; No Touch SF: 2.74 (0.68) In(msec2), *d* = 1.29; Reunion: 2.83 (0.75) In(msec2), *d* = 1.11	Condition Effect (SF and Reunion versus Baseline in both tasks); Did not test for Sex Effect	None reported
RSA	Fracasso et al. (1994)^*,^^	**44−58/5, 7, 10, and 13 months**	5 months: 3.02 (0.71) In(msec2); 7 months: 3.25 (0.72) In(msec2); 10 months: 3.27 (0.72) In(msec2); 13 months: 3.20 (0.71) In(msec2)	7 months: 2.97 (0.86) In(msec2), *d* = 0.37; 10 months: 3.25 (0.68) In(msec2), *d* = 0.03; 13 months: 3.13 (0.75) In(msec2), *d* = 0.10	Condition effect; No Age Effect; Did not test for Sex Effect	NA
RSA	Gray et al. (2017)^^^	**167/4 months**	PP1 = 2.7 (0.47) In(msec2	SF = 2.53 (0.55), *d* = 0.33; PP2 = 2.56 (0.61) In(msec2), *d* = 0.26	Did not test for Condition Effect; Sex Effect (Boys with prenatal stress ↑ RSA in PPI compared to girls)	NA
RSA	Ham and Tronick (2006)	**12/5 months**	FF: Recovered: 3.3; Stable: 3.7; Dysregulated: 3.5; Protest: 3.5 [units unknown; pilot study]	SF‐Recovered: 3.5; Stable: 3.5; Dysregulated: 3.5; Protest: 3.0; Reunion: Recovered: 4.4; Stable: 4.2; Dysregulated: 3.9; Protest: 3.0, all ds = NA [units unknown]	Descriptive; Did not test for Condition or Sex effect	None reported
RSA	Hill‐Soderlund et al. (2008)^^^	**84/13 months**	3.70 (1.03) In(msec2)	Episode 3:3.71 (1.06) In(msec2), *d* = 0.01; Episode 4:3.64 (1.10) In(msec2), *d* = 0.06; Episode 5:3.53 (0.90) In(msec2), *d* = 0.18; Episode 6:3.39(1.16) In(msec2), *d* = 0.21; Episode 7:3.5 (0.98) In(msec2), *d* = 0.20; Episode 8:3.66 (1.07) In(msec2), *d* = 0.04	Condition Effect (Baseline versus Episodes 5, 6, and 7); No Effect of Sex	NA
RSA	Holochwost et al. (2014)^^^	**95/6 months**	3.72 (0.91) In(msec2)	Normal: 3.57 (1.00) In(msec2), *d* = 0.16; SF: 3.46 (1.09) In(msec2), *d* = 0.26; Reunion: 3.51 (1.08) In(msec2), *d* = 0.31	Did not test for Condition or Sex Effect	NA
RSA	Johnson et al. (2014)^^^	**41/6 months**	3.58 (1.76) In(msec2)	Start Arm Restraint: 3.42 (1.71) In(msec2), *d* = 0.09; End Arm Restraint: 2.91 (2.43) In(msec2), *d* = 0.32; Start SF: 3.42 (2.62) In(msec2), *d* = 0.07; End SF: 2.81 (2.31) In(msec2), *d* = 0.38	Did not test for Condition Effect; No Effect of Sex	None reported
RSA	Moore (2009)	**48/6 months**	NA	NA, *d* = NA	Condition Effect (SF, reunion versus Play); Sex Effect (boys had ↑ RSA at baseline)	↑ negative affect = ↓ ΔRSA in each SFP episode
RSA	Moore and Calkins (2004)^*,^^	**60/3 months**	2.82 (0.75) In(msec2)	Play: 2.84 (0.76) In(msec2), *d* = 0.04; SF: 2.64(0.71) In(msec2), *d* = 0.24; Reunion: 2.92 (0.89) In(msec2), *d* = 0.12; Change in RSA Play: −0.02 (0.47), *d* = 0.03; SF: 0.18 (0.46), *d* = 0.28; Reunion: −0.11(0.65), *d* = 0.14	Condition Effect (Baseline versus SF; Play and SF; SF versus Reunion; RSA change SF versus Play and Reunion); No Sex Effect	None reported
RSA	Moore et al. (2009)^^^	**89/6 months**	3.68 (0.85) In(msec2)	FF: 3.50 (0.93) In(msec2), *d* = 0.20; SF: 3.38 (0.96) In(msec2), *d* = 0.33; Reunion: 3.44 (0.95) In(msec2), *d* = 0.27	Condition Effect (Baseline versus SF); No Effect of Sex	↑ positive and negative affect = ↑ RSA in FP; ↑ positive affect = ↑ RSA in Reunion; ↑ positive and negative affect = ↑ HP in reunion
RSA	Noten et al. (2019b)^^^	**116/6 months**	Still‐face: 3.39 (0.38) In(msec2); car seat: 3.30 (0.40) In(msec2)	Still‐face: 3.22 (0.42) In(msec2), *d* = 0.44; Car seat: 3.30 (0.55) In(msec2), *d* = 0	Condition Effect (SF versus Baseline); Did not test for Sex Effect	↑ comfort seeking = ↑ RSA for still‐face
RSA	Paret et al. (2015)^*,^^	**48**/44 months	7.37 (1.08) In(msec2)	6.68 (1.11) In(msec2), *d* = 0.64	Condition Effect (RSA ↓ in IbS); No Effect of Sex	No correlations
RSA	Perry et al. (2012)^^^	**197/42 months**	6.60 (1.12) In(msec2)	5.34 (1.07) In(msec2), *d* = 1.15; Suppression: 1.26 (0.73)	Did not test for Condition Effect; No Effect of Sex	↑ distraction = ↑ RSA suppression
RSA	Perry et al. (2016)^^^	**230/5 and 10 months**	5 months: 3.88 (1.18) In(msec2); 10 months: 4.61 (1.10) In(msec2)	Withdrawal 5 months: −0.54 (1.45), *d* = 0.41; 10 months: −0.10 (1.40), *d* = 0.08	Did not test for Condition or Age Effect; No Effect of Sex	↑ maternal orienting = ↑ RSA withdrawal at 10 months
RSA	Pratt et al. (2015)^^^	**122/5 months**	NA	Change: 0.4 (0.73), *d* = 0.71; Reunion: 2.83 (1.01) In(msec2); all *d* = NA	Did not test for Condition Effect; No Effect of Sex	↑ distress and ↓ disengagemen*t* = ↑ Change RSA
RSA	Provenzi et al. (2015)	**65/4 months**	NA	NA, *d* = NA	No Effect of Condition or Sex.	None reported
RSA	Qu and Leerkes (2018)^^^	**206/14 months**	3.65 (0.99) In(msec2)	SF: 3.40 (1.09) In(msec2), *d* = 0.24; Reengage: 3.51 (1.31) In(msec2), *d* = 0.12	Condition Effect (↓ RSA in SF and Reengagement); No Effect of Sex	↑ negative affect = ↓ RSA in Reengage
RSA	Scrimgeour et al. (2016)	**125/41 months**	NA	Change: 0.00 (1.0), *d* = NA	Did not test for Condition or Sex Effect	NA
RSA	Stone and Porter (2013)	**78/6 months**	If infant has bradycardia or not	Bradycardia: 3.22 (0.75) In(msec2); NonBradycardia: 3.43 (0.78) In(msec2), all ds = NA	Did not test for Condition Effect; No Effect of Sex	NA
RSA	Wagner et al. (2018a)^^^	**97/24 months**	4.86 (0.98) In(msec2)	Suppression: 0.71 (0.77), *d* = 0.88	Did not test for Condition Effect; No Effect of Sex	NA
RSA	Wagner et al. (2018b)^^^	**84/48 months**	6.20 (1.18) In(msec2)	Anger: 6.34 (1.09) In(msec2), *d* = 0.12; Augmentation: −0.14 (range = 0.83 to −2.19)	Condition Effect; No Effect of Sex	NA
RSA	Weinberg and Tronick (1996)^*,^^	**45/6 months**	3.165 In(msec2)	SF: 3.03 In(msec2); Reunion: 3.237 In(msec2), *d* = 0.36	Condition Effect (↓RSA in SF); Did not test for Sex Effect	None reported
RSA	Zeytinoglu et al. (2019)^^^	**244/56 months**	7.21 (1.11) In(msec2)	Locked box: 6.17 (1.14) In(msec2), *d* = 0.92; Toy removal: 6.84 (1.13) In(msec2), *d* = 0.33	Did not test for Condition or Sex Effect	↑ RSA = emotion regulation composite for toy removal only

Abbreviations. HR, heart rate; HRV, heart rate variability; RSA, respiratory sinus arrhythmia; NA, not available.

Notes: (1) Studies included in meta‐analysis are denoted by a symbol; ^*^HR, ^^^RSA, ^#^HRV; (2) $ is used to denote studies that calculated HRV on raw data or used performed transformations on the data that did not allow collapsing into overall RSA meta‐analysis.

### 3.5 Meta‐regressions exploring relationships between physiological measurement and age

**Figure 3:**
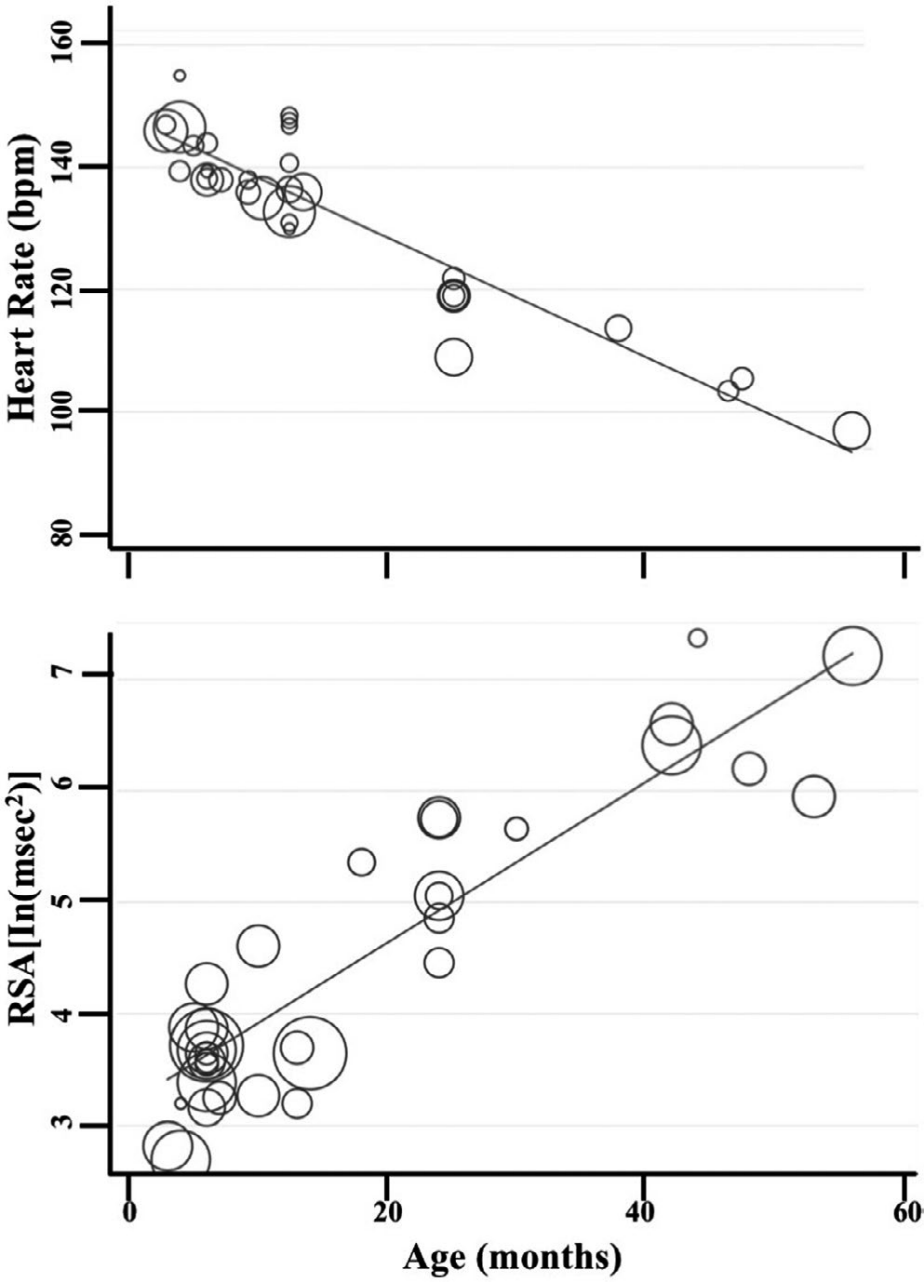
Meta‐regression on baseline means for heart rate (HR; top) and respiratory sinus arrhythmia (RSA; bottom) by age. Note: A paper comparing children at different ages may appear more than once

Figure 3 summarizes the results of the meta‐regression on baseline HR (bpm; top) and RSA (In(msec^2^); bottom) by age, with mean values (where available) for each study plotted against the age of the participants and weighted by the standard error calculated from the meta‐analysis. In general, baseline values for HR decrease with age (*Coefficient* = −1.04, 95% CI = −1.20 to −0.87), with 84.21% of the proportion of between‐study variance accounted for by age. Weighted point estimates (± standard deviation) for age markers were 147 (2.22) bpm for ≤5 months, 138 (0.89) bmp for 6–11 months, 139 (2.98) bmp for 12–23 months, 118 (1.39) bpm for 24–35 months, and 105 (2.03) bpm for ≥36 months. In contrast, baseline values for RSA increase with age (*Coefficient* = 0.07, 95% CI = 0.06–0.08), with 80.97% of the proportion of between‐study variance accounted for by age. Weighted point estimates (± standard deviation) for age markers were 3.18 (0.13) In(msec^2^) for ≤5 months, 3.64 (0.11) In(msec^2^) for 6–11 months, 4.40 (0.32) In(msec^2^) for 12–23 months, 5.33 (0.15) In(msec^2^) for 24–36 months, and 6.64 (0.18) In(msec^2^) for ≥36 months.

**Figure 4:**
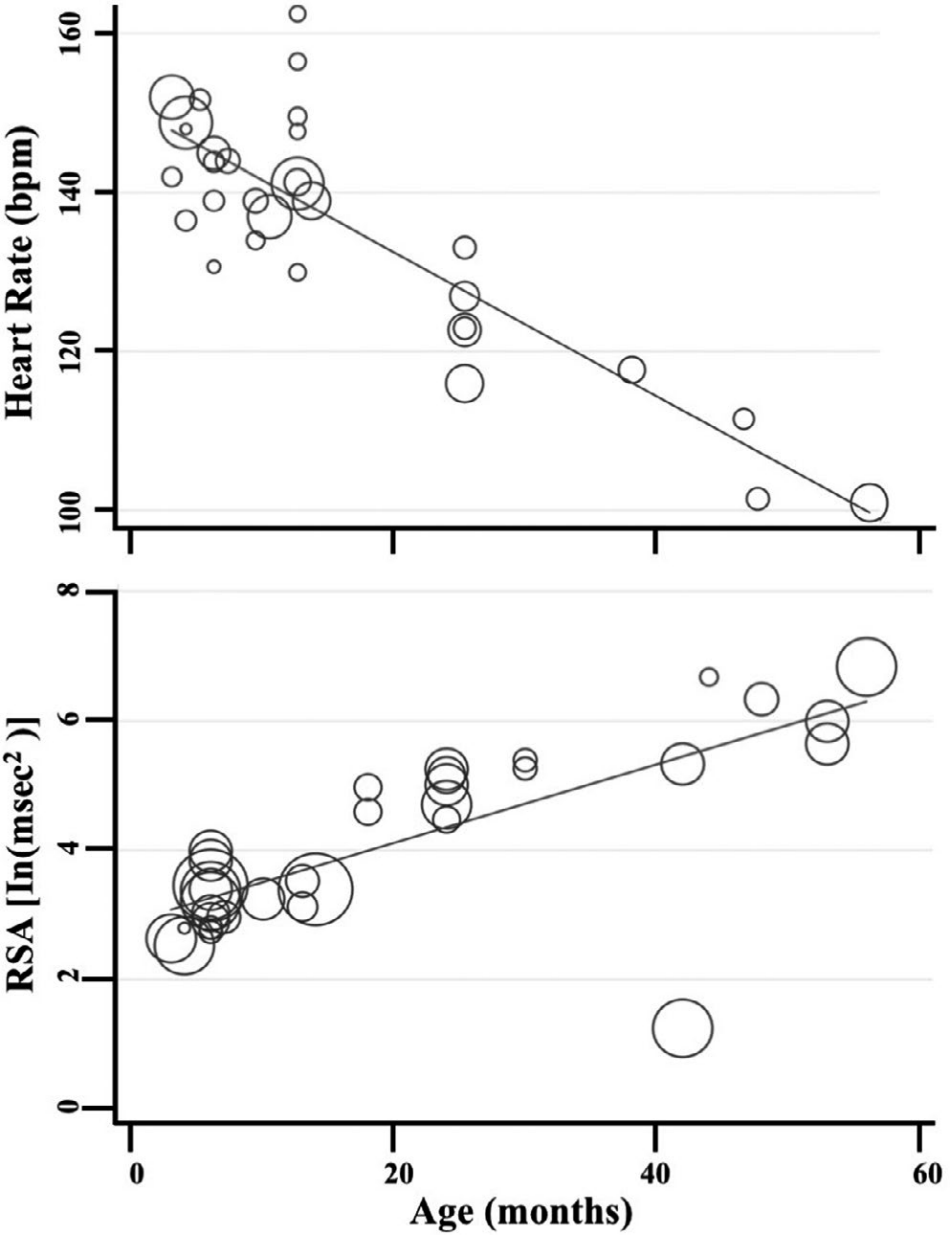
Meta‐regression on task means for heart rate (HR; top) and respiratory sinus arrhythmia (RSA; bottom) by age. Note: A paper comparing children at different ages may appear more than once

Figure 4 summarizes the results of the meta‐regression on HR (bpm; top) and RSA (In(msec^2^); bottom) during the emotion‐evoking tasks, with mean values (where available) for each study plotted against the ages of participants and weighted by the standard error calculated from the meta‐analysis. In general, task values for HR decreased with age (*Coefficient* = −0.96, 95% CI = −1.19 to −0.73), with 71.12% of the proportion of between‐study variance accounted for by age. Weighted point estimates (± standard deviation) for age markers during emotion‐evoking tasks were 145 (2.08) bpm for ≤5 months, 138 (1.94) bmp for 6–11 months, 147 (4.00) bmp for 12–23 months, 125 (1.92) bpm for 24–35 months, and 108 (2.55) bpm for ≥36 months. In contrast, task values for RSA increased with age (*Coefficient* = 0.06, 95% CI = 0.04 to 0.08), with 54.66% of the proportion of between‐study variance accounted for by age. Weighted point estimates (± standard deviation) for age markers during emotion‐evoking tasks were 2.74 (0.04) In(msec^2^) for ≤5 months, 3.17 (0.11) In(msec^2^) for 6–11 months, 4.05 (0.25) In(msec^2^) for 12–23 months, 5.07 (0.11) In(msec^2^) for 24–36 months, and 5.19 (0.46) In(msec^2^) for ≥36 months.

When the baseline values were subtracted from the task values, the relationship with age disappeared for both HR (*Coefficient* = 0.09, 95% CI = −0.06 to 0.25; *R^2^* = 1.57%), and RSA (*Coefficient *= −0.01, 95% CI = −0.03 to 0.003; *R^2^* = 4.56%).

### 3.6 Meta‐analyses on physiological responses to emotion‐evoking tasks

#### 3.6.1 Heart Rate (HR)

**Figure 5:**
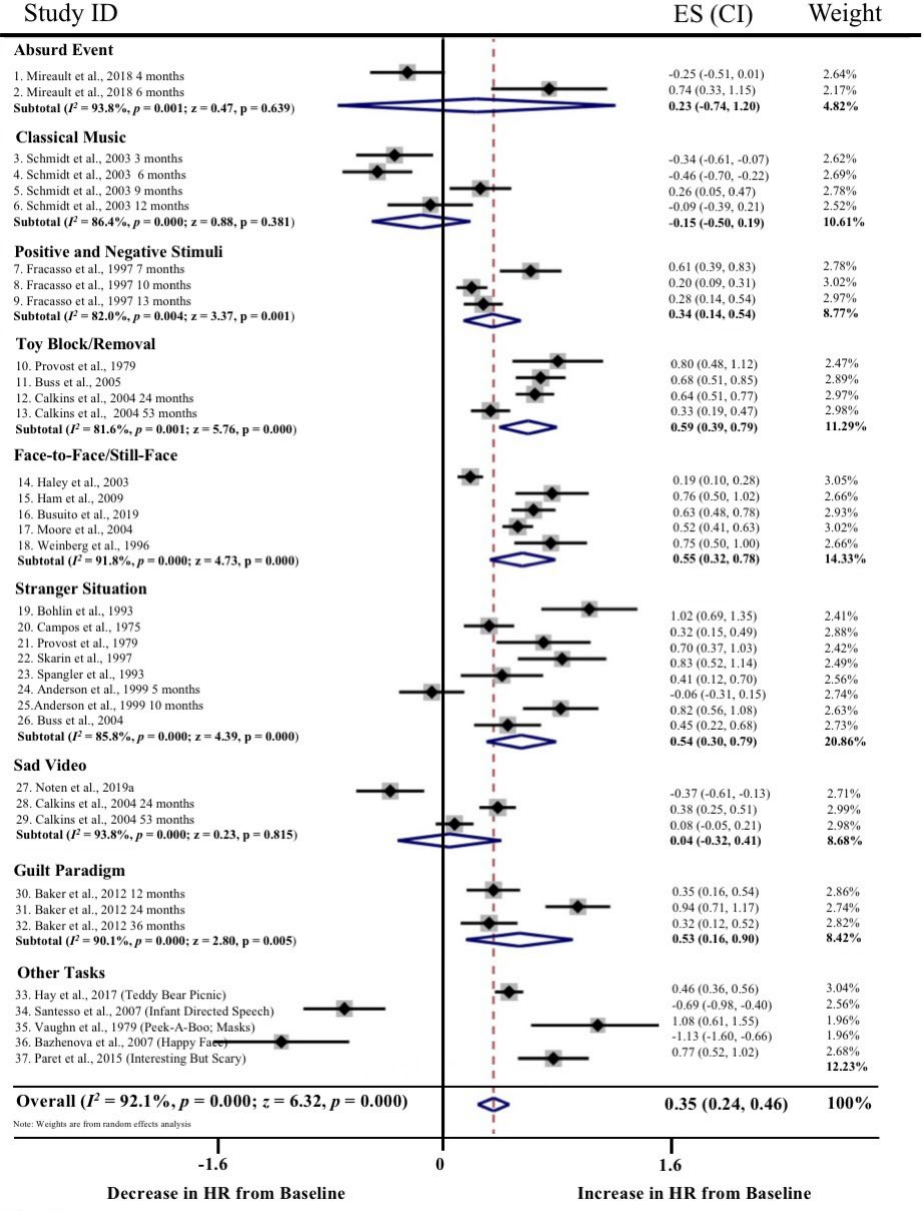
Meta‐analyses on studies examining heart rate (HR) reactivity during an emotion‐evoking task. Note: A paper comparing children at different ages or across different tasks may appear more than once. Abbreviations: ES = Cohen's *d* effect size

**Figure 6:**
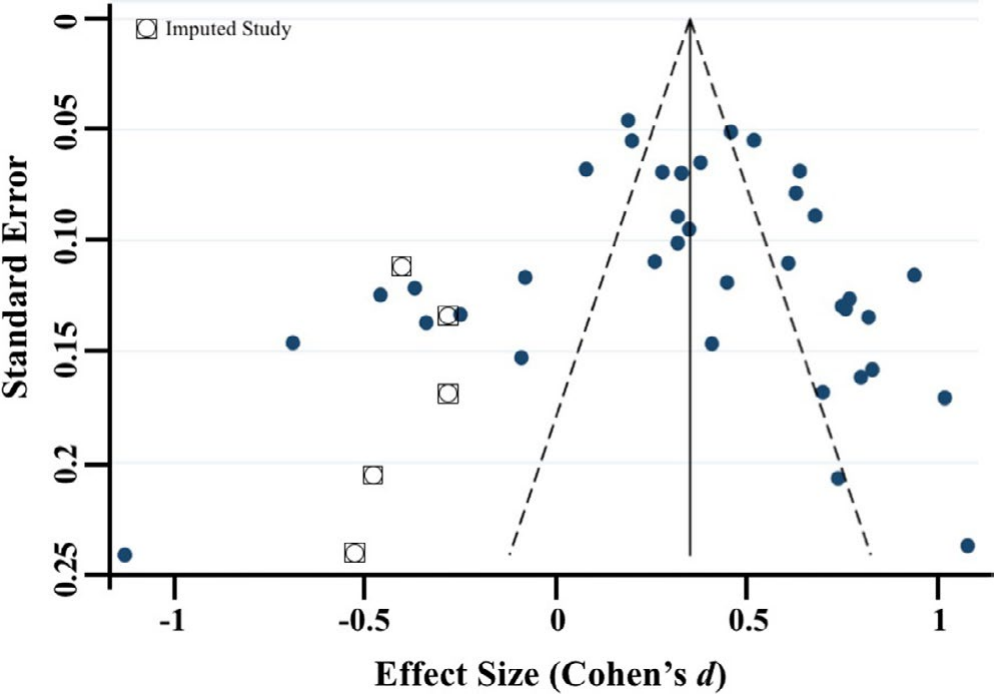
Funnel plot of studies examining heart rate (HR) reactivity during an emotion‐evoking task. Note: A paper comparing children at different ages or across different tasks may appear more than once

A total of 24 studies measuring HR were included in a meta‐analysis. There was a significant effect of emotion‐evoking task, suggesting that tasks produced an increase in HR compared to baseline (Cohen's *d* ES = 0.35, 95% CI = 0.24–0.46, *z* = 6.32, *p* < .001, Figure 5). There was a large heterogeneity effect among the included studies (*I* ^2^ = 92.1%); thus, we adopted a random effects model to pool the relevant data and explored subgrouping analyses to determine any differential effects of the type of emotion‐evoking task on HR. As shown in Figure 5, five of the eight tasks (positive and negative stimuli, toy block/removal, face‐to‐face/still‐face, stranger situation, and guilt paradigm) produced significant effects (all *p*s < 0.005), all resulting in an increase in HR compared to baseline. In contrast, three tasks (absurd event, classical music, and sad videos) did not have a significant effect on HR (all *p*s > 0.38), with no clear pattern of increasing or decreasing HR relative to baseline. Funnel plot analyses on Cohen's *d* ES for HR demonstrated asymmetry, suggesting that bias was present (Figure 6). The trimmed set of data systematically removed each “outlier” one at a time and recalculated the resulting Cohen's *d* ES. The resultant value became lower with each iteration, with the lowest value at 0.27. The missing studies were then imputed with Stata and filled in (symmetrically replacing the trimmed studies and imputing a reflected Cohen's *d* ES around the mean). The addition of the 5 imputed studies resulted in overall Cohen's *d* ES of 0.27 (95% CI = 0.16–0.38) and although lower maintained significance (*z* = 4.75, *p* < .001). Evaluation of the Egger test provided little evidence of small study effects impacting Cohen's *d* ES (bias coefficient = 0.02, standard error = 1.46; *t* = 0.02, *p* = .99).

#### 3.6.2 Respiratory Sinus Arrhythmia (RSA)

**Figure 7:**
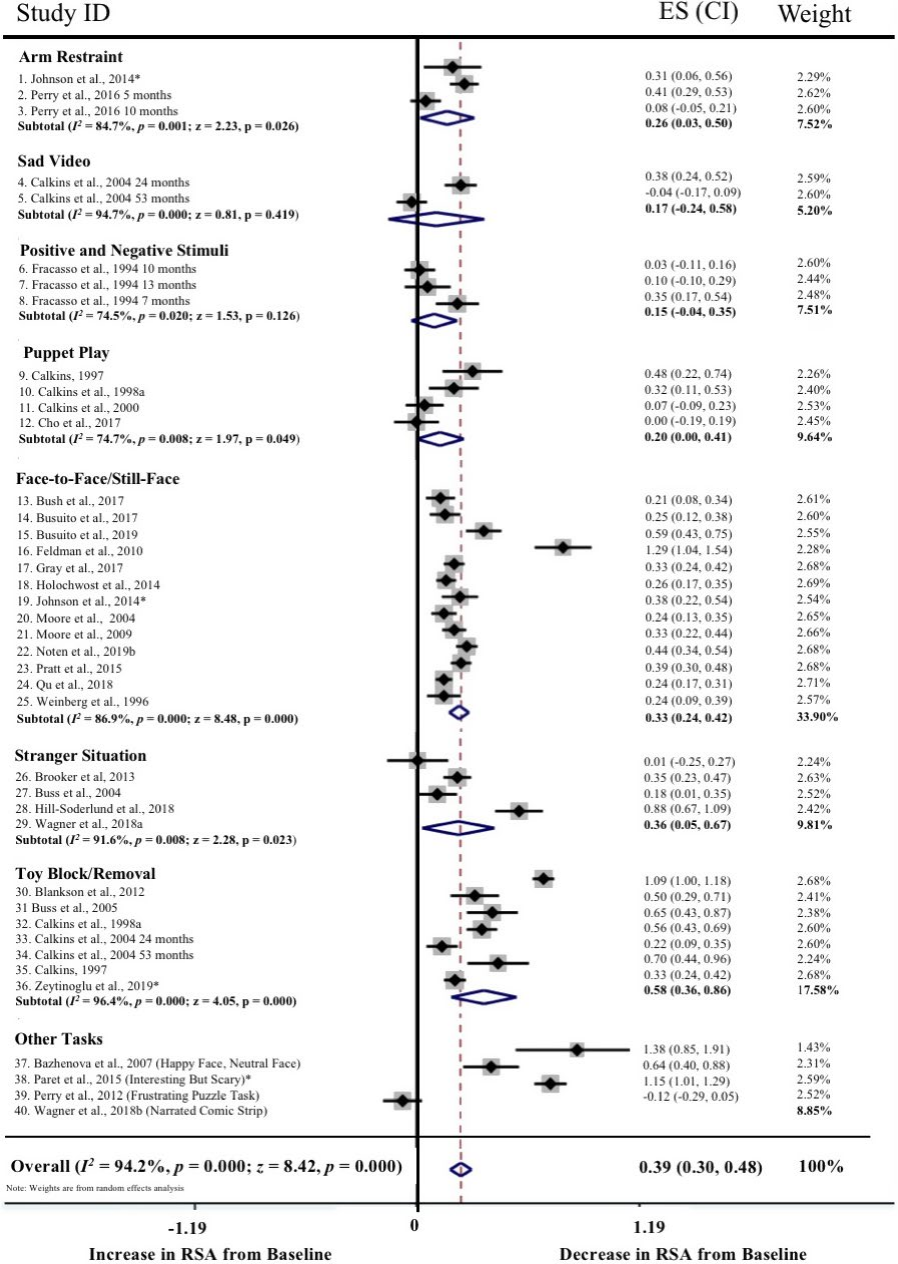
Meta‐analyses on studies examining respiratory sinus arrhythmia (RSA) reactivity during an emotion‐evoking task. Note: A paper comparing children at different ages or across different tasks may appear more than once. Abbreviations: ES = Cohen's *d* effect size; * paper used high‐functioning heart rate variability (HR‐HRV) to calculate RSA

A total of 32 studies measuring RSA were included in a meta‐analysis. There was a significant effect of emotion‐evoking task, suggesting that tasks produced a decrease in RSA compared to baseline (Cohen's *d* ES = 0.39, 95% CI = 0.30–0.48, *z* = 8.42, *p* < .001, Figure 7). There was large heterogeneity among the included studies (*I* ^2^ = 94.2%); thus, we adopted a random effects model to pool the relevant data and explored subgrouping analyses to determine any differential effects of the type of emotion‐evoking task on RSA. As shown in Figure 7, five of the seven tasks (arm restraint, puppet play, face‐to‐face/still‐face, stranger situation, and toy block/removal) produced significant effects (all *ps* < 0.05), all resulting in a decrease in RSA compared to baseline. In contrast, two tasks (“sad videos” and “positive and negative tasks”) did not have a significant effect on RSA (all *p*s > 0.13).

**Figure 8:**
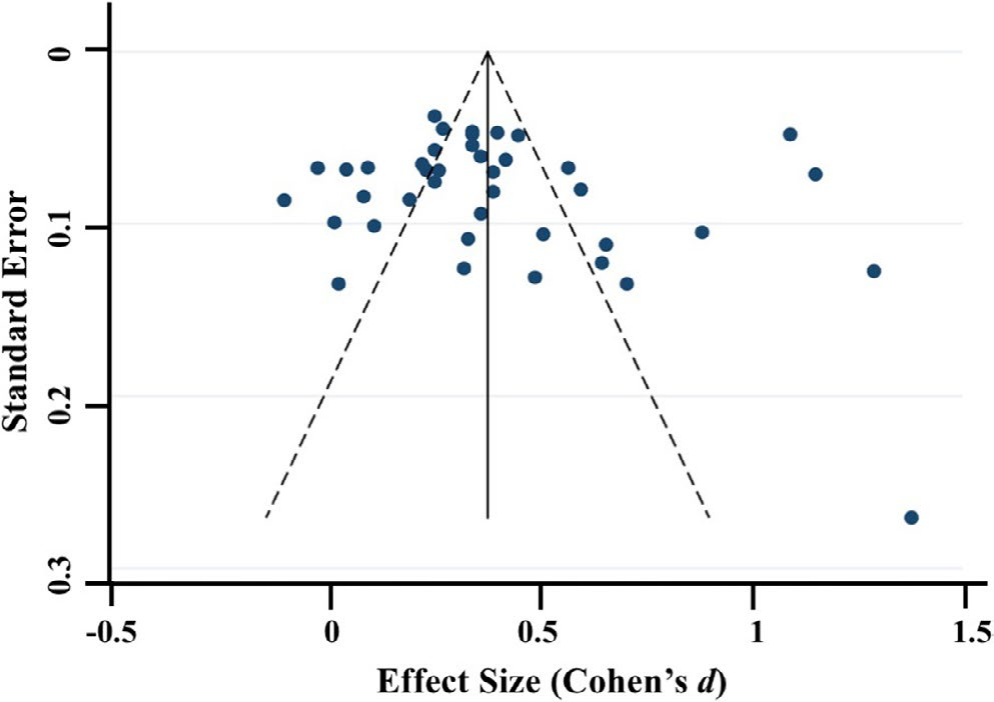
Funnel plot of studies examining respiratory sinus arrhythmia (RSA) reactivity during an emotion‐evoking task. Note: A paper comparing children at different ages or across different tasks may appear more than once

Funnel plot analyses on Cohen's *d* ES for RSA demonstrated symmetry, suggesting that bias was not present (Figure 8). Trim and fill analyses left the data unchanged and the Egger test provided little evidence of small study effects impacting Cohen's *d* ES (bias coefficient = 1.02, standard error = 1.91; *t* = 0.54, *p* = .56).

#### 3.6.3 Heart Rate Variability (HRV)

**Figure 9:**
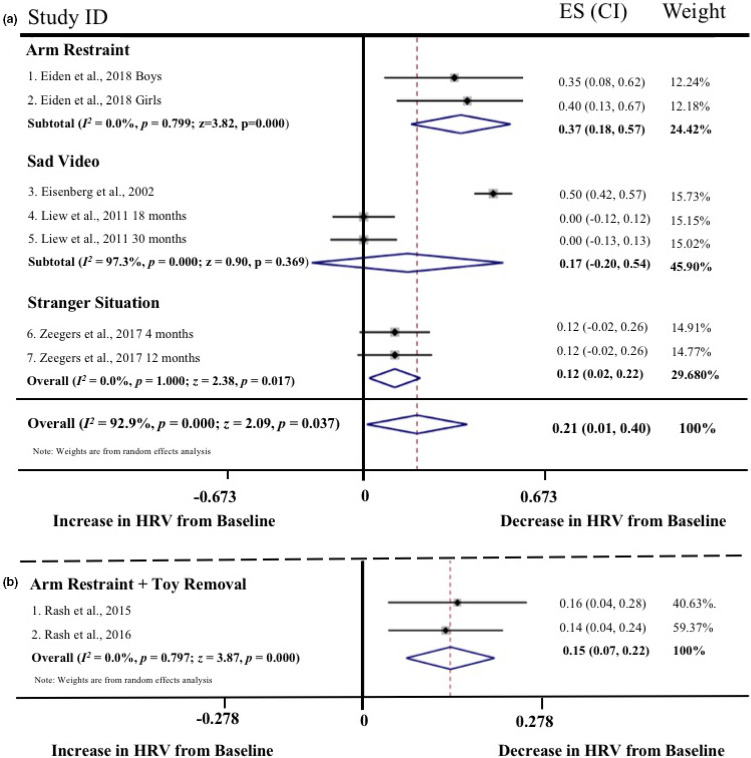
Meta‐analyses on studies examining respiratory heart rate variability (HRV) during an emotion‐evoking task for (a) HRV calculated in milliseconds or seconds and (b) HRV calculated in milliseconds/Hz. Note: A paper comparing children at different ages or across different tasks may appear more than once. Abbreviations: ES = Cohen's *d* effect size

Two separate meta‐analyses were completed for HRV data. The first included four studies that measured HRV in msec or sec (Figure 9a), and the second included two studies that measured HRV in msec^2^/Hz (Figure 9b).

The first meta‐analysis produced a significant effect of emotion‐evoking task, suggesting that tasks produced a decrease in HRV compared to baseline (Cohen's *d* ES = 0.21, 95% CI = 0.01–0.40, *z* = 2.09, *p* = .037, Figure 9a). There was large heterogeneity among the included studies (*I* ^2^ = 92.9%); thus, we adopted a random effects model to pool the relevant data and explored subgrouping analyses to determine any differential effects of the type of emotion‐evoking task on HRV. As shown in Figure 9a, two of the three tasks (arm restraint and stranger situation) produced significant effects (all *p*s < 0.017), with both resulting in a decrease in HRV compared to baseline. In contrast, one task (sad videos) did not have a significant effect on HRV (*p* = .37).

**Figure 10:**
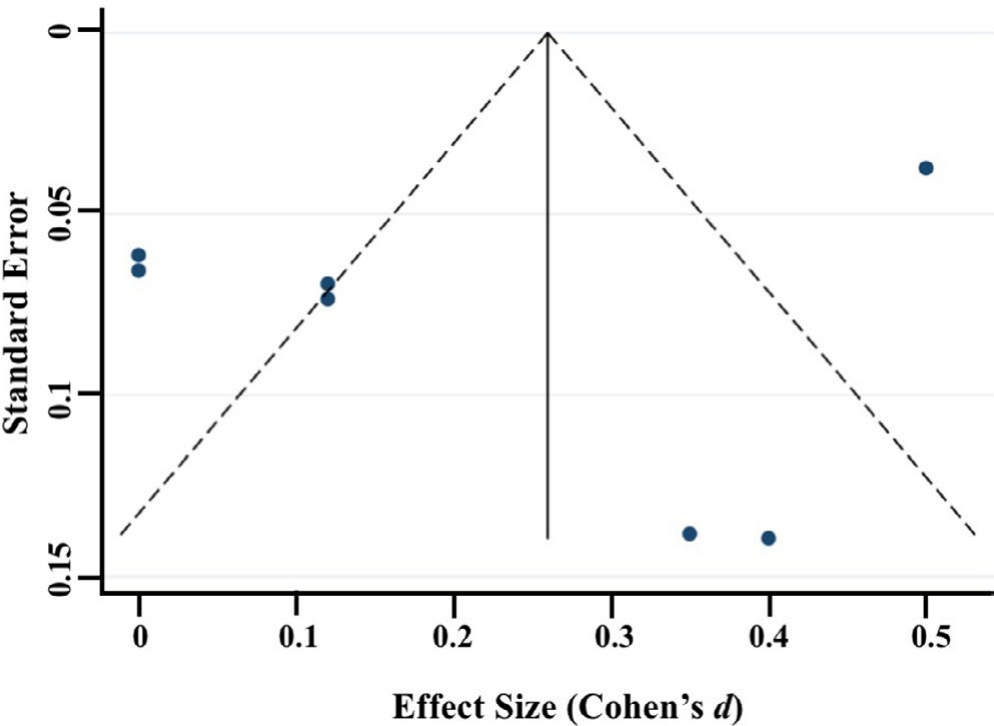
Funnel plot of studies examining heart rate variability reactivity (from A above) during an emotion‐evoking task. Note: A paper comparing children at different ages or across different tasks may appear more than once

Funnel plot analyses on Cohen's *d* ES for HRV demonstrated symmetry, suggesting that bias was not present (Figure 10). Trim and fill analyses left the data unchanged and the Egger test provided little evidence of small study effects impacting Cohen's *d* ES (bias coefficient = −3.77, standard error = 3.59; *t *= −1.05, *p* = .34).

The second meta‐analysis produced a significant effect of emotion‐evoking task, suggesting that the task produced a decrease in HRV compared to baseline (Cohen's *d* ES = 0.15, 95% CI = 0.07–0.22, *z* = 3.87, *p* < .001, Figure 9b). There was no heterogeneity between the two studies (*I* ^2^ = 0%). Due to the small number of studies included, funnel plot and Egger test were not performed.

### 3.7 Relationships between physiological measurement and behavioral coding

In addition to collecting electrocardiograph (ECG) data, the majority of studies also coded affective behavior in response to the emotion‐evoking tasks. Most studies included coding of observed behavior in their protocols, as summarized in Table 3. Behavioral coding included verbal responses (e.g., laughing, signs of distress), physical reactions (e.g., pushing away, covering face), body tension/posture, facial affect (e.g., smiling, frowning), gaze (e.g., look at object, look to mother), motor reactivity, regulatory behaviors, duration/frequency of crying, signs of distress or fear, and touch.

HR was positively associated with:

1. wariness during stranger tasks (Anderson et al., 1999; Bohlin & Hagekull, 1993)
2. gaze aversion during stranger tasks (Waters et al., 1975)
3. negative affect during stranger tasks (Buss et al., 2005; Campos et al., 1975; Ham & Tronick, 2009)
4. vocal distress during Teddy Bear picnic (Hay et al., 2017)
5. gaze to parent during an absurd task in 6‐month‐olds (Mireault et al., 2018)
6. social engagement during play (Ham & Tronick, 2009)
7. protest behavior during a still‐face paradigm (Ham & Tronick, 2009)
8. negative affect during reunion (Moore & Calkins, 2004)

HR was inversely related to:

1. gaze to parents during an absurd task at 4 months (Mireault et al., 2018)
2. freezing behavior during a stranger task (Buss et al., 2004)
3. mother engagement during play (Ham & Tronick, 2009)
4. positive and negative affect in free play and reunion (Busuito et al., 2019)
5. negative affect during still‐face (Busuito et al., 2019)

RSA was positively associated with:

1. positive and negative affect during play with mother (Moore et al., 2009)
2. mother engagement during play with mother (Ham & Tronick, 2009)
3. positive affect during reunion (Busuito et al., 2019; Moore et al., 2009)
4. distress during a barrier task (Calkins & Johnson, 1998b)
5. distraction during a barrier task (Calkins & Johnson, 1998b)
6. comfort seeking during still‐face task (Noten et al., 2019b)
7. emotion regulation during a toy removal task

RSA was inversely related to:

1. fear in stranger tasks (Brooker et al., 2013)
2. negative affect during still‐face paradigm (Qu & Leerkes, 2018)
3. aggression during a barrier task (Calkins & Johnson, 1998b)

RSA suppression was inversely related to:

1. fear during arm restraint (Cho & Buss, 2017)
2. negative affect in a still‐face paradigm (Moore, 2009)

HRV suppression was inversely related to:

1. regulation during clips of positive and negative stimuli (Eiden et al., 2018)

The relationships between physiology and observed behaviors described in the 64 studies are presented in Table 4.

## 4 DISCUSSION

The present review summarized the results of research that has examined physiological measurements of emotional regulation in children age 4 years or younger. Three measures of cardiac activity were reported here for use in developmental psychobiological research in young children—heart rate, heart rate variability (HRV), and respiratory sinus arrhythmia (RSA). The review had three main findings. First, meta‐regressions exploring the relationship between age and physiological measurement during baseline and emotion‐evoking task showed a significant effect of age for both baseline and task during measurement of heart rate and RSA. Second, the three meta‐analyses on the impact of emotion‐evoking task on physiological measurement resulted in significant Cohen's *d* effect sizes, with a resulting increase in heart rate from baseline, and a resulting decrease in HRV and RSA from baseline. Third, physiological measurement was related to observed behaviors (e.g., facial affect and gaze) during emotion‐evoking tasks. Having a good understanding of physiological and age‐related changes of typically developing children for both baseline and emotion‐evoking tasks is vital when understanding the role of emotional regulation in typical and atypical development.

### 4.1 Review of the findings

Physiological measurement of responses to an emotion‐evoking task typically begins with a baseline (resting) period. This period is intended to reflect the individual's innate capacity to regulate their emotions (Appelhans & Luecken, 2006), such that higher baseline heart rate variability (higher PNS activity) reflects more regulatory capacity (Propper & Moore, 2006) and is associated with positive psychosocial outcomes (Scarpa et al., 2010). An overview of the baseline tasks reviewed here indicated variability, both in terms of duration, from 5 (Anderson et al., 1999; Bohlin & Hagekull, 1993; Skarin, 1977) to 420 s (Busuito et al., 2019), and task (including sitting quietly, playing alone or with mother, time immediately before or between trials, and watching short videos, and “baseline period”). An examination of the plot of mean durations for the two physiological measurements (as weighted by standard error) revealed that mean baseline HR decreased with age, whereas mean RSA increased with age. These results echo previous reports of age‐related changes in samples of children who were repeatedly assessed between birth and 4 to 5 years of age (Bar‐Haim et al., 2000; Bornstein & Suess, 2000; Porges et al., 1994; Sheinkopf et al., 2019). Although there is no consensus on age‐related values for heart rate or RSA during baseline or emotion‐evoking tasks, the weighted point estimates derived from the meta‐regressions in this study may serve as tentative guidelines for developmental changes in children aged 4 and younger.

Although the emotion‐evoking tasks probed for a variety of emotional responses, the vast majority explored responses to (putative) negative emotions, including anger, disappointment, distress, fear, frustration, and guilt, with only a minority of studies exploring positive emotion‐evoking tasks. Our results are similar to those reported by Kreibig (2010), who noted that studies examining fear, anger, or anxiety (as probed by stranger situation, still‐face, arm restraint, or toy removal/block in the studies reviewed here) reliably produce an increase in HR or a decrease in RSA and HRV from baseline in children 6 months of age and older. Of the studies that did not have an effect, or produced a discordant result (decrease in HR, increase in RSA or HRV from baseline), the participants were either under 6 months of age (Anderson et al., 1999; Bazhenova et al., 2007; Mireault et al., 2018), or the task or had an element of passivity and may not have been sufficiently salient to produce reactivity (e.g., classical music, infant directed speech, puppet play, narrated comic strip; or watching a sad video; Calkins et al., 2007; Calkins & Keane, 2004; Cho & Buss, 2017; Liew et al., 2011; Noten et al., 2019a; Schmidt et al., 2003; Wagner et al., 2018b). Passivity was identified by Kreibig (2010) as having differential impacts on reactivity.

Of importance, there were instances in which studies had no effect (confidence interval of the effect size crossed zero) but did not conform to the above reasons. Each of these studies was explored to determine whether methodological differences could explain their discrepant results. Although Perry et al. (2016) did not find a difference between baseline and task in 10‐month‐old infants when assessing arm restraint, this may be explained by the role of the mother. The methods described that the mother first played with toys with her child, followed by a game of peek‐a‐boo, and then engaged in arm restraint. It is possible that infants interpreted this as another game due to the preceding play, giving a different tone to the “negative” arm restraint task. Two studies that used “stranger situation” also found discordant results. An examination of their methods suggested that the participant pool of Brooker et al. (2013) consisted of 6‐month‐old twin pairs, who have different developmental trajectories due to variations in gestational age. Zeegers et al. (2017) identified that they did not use age‐adjusted respiration rates in their analyses. Finally, although Fracasso et al. (1994) did not describe the specific emotion‐evoking task used, they reported that “a variety of stimuli designed to elicit both positive and negative emotions” (p. 279). Because the emotion‐evoking tasks were not described, an evaluation based on methodological differences cannot be provided, beyond considering that combining positive and negative values potentially washed out any differential responses (Kreibig, 2010).

In addition to describing age‐related changes in HR and RSA, we also detailed relationships between physiological measurements and observed behavioral responses. For example, increases in heart rate were associated with increased wariness, gaze aversion, and negative affect during stranger situation and decreases in RSA were associated with increased negative affect in the still‐face paradigm. Exploring relationships between external (behavioral) and internal (physiological) reactivity may provide important insights into overall emotional regulation. Calkins and Dedmon (2000) noted that physiological and behavioral regulation may be interdependent components with changes to emotional reactivity apparent on both physiological and behavioral levels. If observed behavior and physiological reactivity are indeed interdependent, one would expect to find a significant association between behavioral and physiological reactivity for a majority (if not all) of the studies in this review that included behavioral coding. Yet several did not report any such significant relationship. Identifying congruence and incongruence between behavioral and internal reactivity is important, especially when one consider atypical populations. For example, heart rate and facial affect were compared in 4–6 year old typically developing children and children with autism spectrum disorder (ASD) who were presented with a fear‐inducing robot. Both the typically developing children and children with ASD showed task‐related heart rate changes (and did not differ from one another) but only the typically developing children showed changes to facial affect reflecting a fearful response (no affect changes were seen in the children with ASD; Zantinge et al., 2019). This example is particularly illustrative of the importance of describing associations between internal and external reactivity and how they may differ in childhood disorders.

### 4.2 Physiological reactivity

Emotional regulation is an adaptive skill that children develop during their early years (Calkins, 1994; Eisenberg et al., 1995; Eisenberg et al., 1995; Kopp, 1982; Thompson, 1994) and undergirds components of personality, social competence, and externalizing and internalizing behavior (Calkins, 1994; Calkins & Keane, 2004; Cicchetti et al., 1991; Cole et al., 1994; Stifter et al., 1999). If this process is interrupted, emotion dysregulation may contribute to the development of psychopathology (Calkins & Dedmon, 2000; Calkins & Fox, 1992; Keenan, 2000; Shaw, Keenan, Vondra, Delliquadri, & Giovannelli, 1997). The polyvagal theory is one framework for understanding how the PNS fosters adaptive engagement with the environment (Porges, 2007), and authors of several studies in this review explored this framework within their research. The direction and magnitude of PNS activity can help differentiate how stimuli are interpreted (termed *neuroception)*. Small‐to‐medium increases in RSA, for example, connote that the individual interprets the environment as socially engaging and safe (i.e., rest and digest), whereas small‐to‐medium decreases in RSA (as identified by the studies in this review that used RSA as their physiological measure) connote that the individual interprets the environment as threatening, thus preparing the body for action (i.e., fight or flight; Appelhans & Luecken, 2006; Calkins & Keane, 2004; Hastings et al., 2014; Porges, 2007; Porges et al., 1996). These states are mutually exclusive—if you do not feel safe, you are more likely to be in a physiologically aroused state, affording less time for social engagement—which can have cascading impacts on development. As such, understanding how typically developing children respond to various environmental challenges, such as those modeled in the emotion‐evoking tasks described in this review, will allow us to better understand atypical development. This is important because atypical emotional responses and dysregulation may have implications for functional outcomes including language and social skills (Carpenter & Tomasello, 2000; Mundy & Sigman, 2006; Woods & Wetherby, 2003).

Important to understanding emotion reactivity is delineating if emotions are associated with differential reactivity. Feldman‐Barrett (2006) contends that emotions do not have unique autonomic signatures, but rather may denote differences between positive and negative states. The ANS is activated in response to actual or expected behavior, and because behavior is not emotion‐specific, Feldman‐Barrett (2006) contends that emotion‐specific ANS patterns are improbable. Furthermore, ANS differences between emotions are viewed as *dimensional differentiation*, with mediators purported to explain the heterogeneity of findings in meta‐analyses (Feldman‐Barrett, 2006). Cacioppo et al. (1997) contend that there is a degree of differentiation between emotions, with differences in valence‐specific tasks (e.g., negative versus positive) producing more consistent results than emotion‐specific (joy, fear) tasks. In contrast, Stemmer has posited that different emotions have inherently different goals (e.g., fear to escape situation, happiness to engage in situation) and thus should have different ANS responses (Stemmler, 2004, 2009). Our findings examining physiological measurement in children aged 4 and under support Feldman‐Barrett's (2006) and Cacioppo et al., (1997) position. When looking at the pattern of HR, RSA, and HRV data in this review, there appears to be support for Stemmer (2004, 2009) supposition of valence‐specific task differences, but as addressed above, these differences may result from the methodology employed. Other reviews that included adolescents and adults as their population of interest lend more support to Stemmer's position. In her review of 134 studies examining 22 different emotion categories, Kreibig (2010) identified differential responses in HR depending on the probed emotion. The results were less straight‐forward when considering HRV (including RSA), as there are various methods of calculating values and patterns of results differ depending on the method used. There is still additional research needed to fully address this debate.

### 4.3 Considerations for data collection and analyses

When assessing emotions in young children, it is important to determine whether the task is producing the desired response (i.e., does a “negative” task produce negative emotional responses in the child?). For example, in the laboratory temperament assessment battery (Lab‐TAB; Goldsmith & Rothbart, 1996), children are presented with four masks, an evil queen (from *Snow White*), a glow‐in‐the‐dark vampire, an old man, and a gas mask. These masks are designed to elicit a fear response and the scoring of this task reflects this, coding for both fear and sadness. Although some children may show these affective responses when presented with these masks, some children may react by smiling or laughing. In addition, there could be a group of nonresponders (for observed affect) who show a cardiac response and another group of nonresponders (for observed affect) who do not. This inter‐individual variability could wash out general associations.

Individual variability may also be impacted by psychomotor activity. It may be helpful for researchers to note the effects of movement, as well as postural differences and supports required for infants, toddlers, and preschoolers as a part of their data collection and analysis. The studies reviewed here made little mention of the impact of movement, other than editing or removing movement artifacts in their data, nor of the relative contributions of postural support. Data that require editing due to movement or postural instability (i.e., sections of the data that are edited or removed from analyses) may be confounded by psychomotor activity and thus may under‐ or over‐estimate the impact of emotion‐evoking stimuli (Bush et al., 2011).

Another consideration for physiological measurement when examining age‐related changes is body size. Body size was not mentioned in the studies, nor body size included as a factor in analyses of HR, RSA, or HRV. The allometric law of mammal's states that as body size increases, heart rate decreases (Meijler, 1985). Because basal cardiac activity reflects the ability of an individual to utilize their ANS to appropriately respond to environmental challenges (Porges, 2007), future research will need to consider body size in addition to age when assessing cardiac responses in early childhood, as rates of obesity are increasing in children (World Health Organization, 2018), and when assessing children with developmental disabilities, including autism spectrum disorder, in whom weight‐related challenges may be particularly common (Levy et al., 2019).

Finally, when studying various emotion‐evoking tasks and comparing reactivity across tasks, such as puppets, toy play, toy removal, and others included in Lab‐TAB (Goldsmith & Rothbart, 1996), it may be important to include a neutral event between tasks to ensure reactivity in one task does not bleed into or influence reactivity in following task. This is especially important when examining the differential roles of SNS and PNS activity, in which a period of recovery from stress is warranted (Suurland et al., 2017). In light of this, unexpected responses and variability should be reported for both positive and negative tasks, and a neutral event should be included between emotion‐evoking tasks to reduce potential carry‐over effects that may influence variability, as well as at the end to allow for SNS and PSN comparison and recovery.

With the findings of the review and the above considerations in mind, we recommend the following for future physiological studies of emotion reactivity in children. First, there should be a baseline period of a minimum of 30 s and preferably two minutes prior to the onset of an emotion task, to allow for data loss due to artifacts, movement, possible electrode removal by the child, and distress. Second, the emotion‐evoking task should be a minimum of 30 s (again, longer durations are preferred to allow for data loss). Third, any study that presents more than one emotion‐evoking task should include a recovery period between tasks to allow for a return to baseline and reduce the likelihood of reactivity in one task bleeding into reactivity related to subsequent tasks. Fourth, a recovery period should be included at the end of the emotion task protocol to allow for PNS and SNS recovery and comparison. Fifth, if RSA (or other HRV) is calculated, age‐corrected respiration frequencies should be employed and clearly stated in the methods section. Sixth, behavioral coding (e.g., affect, gaze, vocalizations) should be included (where appropriate to research question) to allow for an examination of the association between physiological and behavioral indices of reactivity. Seventh, body measurements (height, weight, BMI) should be collected and included in analyses to control for the impact of body size on physiological reactivity. Eighth, the activity of the child during baseline and emotion task should be measured and included in analyses to control for movement.

## 5 CONCLUSION

This methodological review examined physiological responses to emotion‐evoking tasks in children age 4 years or younger. Although we aimed to be inclusive by searching four databases without language or publication date restrictions, we cannot discount that articles may have been missed that were not indexed in our chosen databases. Furthermore, in our aim to be inclusive of all available data, the results from some studies were included more than once in the analyses. We recognize that this is a bias and potential limitation of the analyses.

This review summarized the results of research that has examined physiological measurement of emotional regulation in children age 4 years or younger. Overall, heart rate showed an age‐related decrease for both baseline and emotion‐evoking tasks whereas RSA showed an age‐related increase for both baseline and emotion‐evoking tasks, regardless of task valence. This review was both necessary and timely to review existing literature for assessing internal responses to emotion in childhood, as emotion regulation has implications for later functional outcomes (Carpenter & Tomasello, 2000; Mundy & Sigman, 2006; Weiss et al., 2014; Woods & Wetherby, 2003), and is a focus of much recent research attention, including in children at risk for mental health disorders (e.g., Eiden et al., 2018) and neurodevelopmental disorders (Sheinkopf et al., 2019). Understanding the potential differences that may arise due to methodological and analytical differences can inform future studies as researchers continue to investigate emotional regulation and reactivity differences in typically developing children and children with an atypical presentation.

## CONFLICT OF INTEREST

The authors have no conflict of interest to disclose.

## AUTHOR CONTRIBUTIONS

LRS, SR, and VA reviewed the literature and approved articles to be included in the review. JAB, AK, IMS, and LZ provided guidance to the research question, search terms, search strategy, technical advice on statistical analyses, and provided constructive feedback on the first draft. LRS did the statistical analyses and wrote the first draft of the manuscript. All authors approved the final draft prior to submission.

### Peer Review

The peer review history for this article is available at https://publons.com/publon/10.1002/brb3.1989.

## Supporting information

Appendix S1

Appendix S1

Table S3

Table S4

Table S5

Table S6
