# Supplementary material for: Physiological measurement of emotion from infancy to preschool: A systematic review and meta‐analysis
Source: Brain Behav. 2020 Dec 17;11(2):e01989. doi: 10.1002/brb3.1989 (PMC7882167; doi:10.1002/brb3.1989)
Supplement: Supplementary file 1 — Appendix S1 [file BRB3-11-e01989-s001.pdf]

Appendix 1

PsycINFO 1806 to March Week 1 2019
Results: 770 on March 8, 2019

1. (emotion* or anxi* or fear or happiness or surprise or guilt or frustrat* or anger or angry).mp.

2. (RSA or heart rate or respiration or respiratory or heart period or physiological reactivity or autonomic nervous system or psychophysiology or pulse or (Physiolog* adj5 measure*)).mp.

3. 1 and 2

4. limit 3 to 160 preschool age <age 2 to 5 yrs>

5. (infant* or toddler* or preschool* or pre-school* or kindergar* or "1-y*-old*" or "2-y*-old*" or "3-y*-old*" or "4-y*-old*" or "5-y*-old*" or "6 m* old*" or "9 m* old*" or "12 m* old*" or "18 m* old*" or "24 m* old*").mp.

6. (3 and 5) or 4

7. Limit 6 to animals

8. 6 not 7

Web of Science Timespan: All years. Indexes: SCI-EXPANDED, SSCI, A&HCI, ESCI.
Results: 1101 on March 8, 2019

1. TOPIC: (emotion* or anxi* or fear or happiness or surprise or guilt or frustrat* or anger or angry)

2. TOPIC: (RSA or heart rate or respiration or respiratory or heart period or physiological reactivity or autonomic nervous system or psychophysiology or pulse or (Physiolog* NEAR/5 measure*))

3. TOPIC: (infant* or toddler* or preschool* or pre-school* or kindergar* or "1-y*-old*" or "2-y*-old*" or "3-y*-old*" or "4-y*-old*" or "5-y*-old*" or "6 m* old*" or "9 m* old*" or "12 m* old*" or "18 m* old*" or "24 m* old*")

CINAHL Plus with Full Text
Results: 518 on March 8, 2019

(emotion* or anxi* or fear or happiness or surprise or guilt or frustrat* or anger or angry) AND (RSA or heart rate or respiration or respiratory or heart period or physiological reactivity or autonomic nervous system or psychophysiology or pulse or (Physiolog* N5 measure*)) AND (infant* or toddler* or preschool* or pre-school* or kindergar* or "1-y*-old*" or "2-y*-old*" or "3-y*-old*" or "4-y*-old*" or "5-y*-old*" or "6 m* old*" or "9 m* old*" or "12 m* old*" or "18 m* old*" or "24 m* old*")

Ovid MEDLINE(R) and Epub Ahead of Print, In-Process & Other Non-Indexed Citations and Daily 1946 to March 07, 2019
Results: 1358 on March 9, 2019

1. (emotion* or anxi* or fear or happiness or surprise or guilt or frustrat* or anger or angry).mp.

2. (RSA or heart rate or respiration or respiratory or heart period or physiological reactivity or autonomic nervous system or psychophysiology or pulse or (Physiolog* adj5 measure*)).mp.

3. 1 and 2

4. (infant* or toddler* or preschool* or pre-school* or kindergar* or "1-y*-old*" or "2-y*-old*" or "3-y*-old*" or "4-y*-old*" or "5-y*-old*" or "6 m* old*" or "9 m* old*" or "12 m* old*" or "18 m* old*" or "24 m* old*").mp.

5. 3 and 4

6. Limit 5 to animals

7. 5 not 6
