# Supplementary material for: Physiological measurement of emotion from infancy to preschool: A systematic review and meta‐analysis
Source: Brain Behav. 2020 Dec 17;11(2):e01989. doi: 10.1002/brb3.1989 (PMC7882167; doi:10.1002/brb3.1989)
Supplement: Supplementary file 2 — Appendix S1 [file BRB3-11-e01989-s002.docx]

| Appendix 2. Study Descriptive Characteristics | | | | |
| --- | --- | --- | --- | --- |
|  | **Design and Setting** | **Participants Characteristics**  **Original sample size; (n girls); ethnicity; SES status** | **Inclusion Criteria** | **Origin** |
| **Under 6 months of age (n = 19)** | | | | |
| Anderson et al 1999 | Longitudinal Laboratory | 45 infants (22 girls) assessed at 5 and 10 months; ethnicity not provided; SES not provided | Healthy, full-term, first-born, single born | Sweden |
| Bazhenova et al 2007 | Cross-Section Laboratory | 16 infants (9 girls) assessed at 4 months; 100% Caucasian; middle class sample | Full-term, no medical complications | USA |
| Calkins et al. 1992 | Longitudinal Laboratory | 50 infants (25 girls) assessed at 5 months, 52 toddlers (27 girls) assessed at 14 months, and 48 (25 girls) assessed at 24 months; 92% Caucasian; middle class sample | Gestational age 38-42 weeks, birth weight 2,500-4,090g, Apgar scores >7 at 1 min and 8 at 5 min, and normal pediatric examination | USA |
| Campos et al 1975 | Longitudinal  Laboratory | 80 infants, 40 (22 girls) assessed at 5 months and 40 (11 girls) assessed at 9 months; ethnicity not provided; SES not provided | NA | USA |
| Fracasso et al 1994 | Longitudinal Laboratory | 58 infants (32 girls) assessed at 5 months, 53 (29 girls) at 7 months, 44 (25 girls) at 10 months, and 49 (24 girls) at 13 months; 100% Caucasian; upper-middle class sample | First-born, Caucasian from upper-middle-class, two-parent families | USA |
| Gray et al. 2017 | Cross-Section Laboratory | 167 infants (82 girls) assessed at 4 months; 61% African American, 39% Caucasian; SES not provided | Mothers >18 years of age and English-speaking | USA |
| Haley et al 2003 | Cross-Section In Home | 43 infants (gender NA) assessed at 5 months; 57% European American, 28% Hispanic, 12% mixed ethnicities; SES not provided | Infants not preterm and no serious/chronic medical condition since birth | USA |
| Ham et al 2006 | Cross-Section Laboratory | 12 infants (gender NA) assessed at 5 months; ethnicity not provided; SES not provided | NA | USA |
| Ham et al 2009 | Cross-Section Laboratory | 18 infants (7 girls) assessed at 5 months; ethnicity was diverse, % not provided; SES not provided | Born without complication | USA |
| Mireault et al 2018 | Longitudinal Home | Two groups: (1) 37 infants (18 girls) assessed at 5, 6, and 7 months; ethnicity not provided; middle class sample; (2) 46 infants (27 girls) assessed at 4, 6, and 8 months; ethnicity not provided; middle class sample | Infants were full-term | USA |
| Moore et al 2004 | Cross-Section Laboratory | 72 infants (30 girls) assessed at 3 months (n=60 non-depressed mothers included here); 81% European American, 14% African American, 5% other; primarily middle- to upper class | Delivered full-term singletons, and did not have a score higher than 11 on Becks Depression Inventory | USA |
| Morasch et al 2012 | Longitudinal Laboratory | 106 infants (56 girls) assessed at 5 and 10 months; 88% Caucasian, 5% Hispanic, 7% other; SES not provided | Born within two weeks of due date and no neurological or developmental problems | USA |
| Perry et al 2016 | Cross-Section Laboratory | 230 infants (124 girls) assessed at 5 or 10 months; 78% Caucasian, 13% African American, 9% other; SES not provided | Born within 15 days of calculated due dates and healthy at the time of testing | USA |
| Pratt et al. 2015 | Cross-Section Laboratory | 122 infants (57 girls) assessed at 5 months; 100% Israeli-Jewish; middle class sample | NA | Israel |
| Provenzi et al 2015 | Cross-Section Laboratory | 94 infants (44 girls) assessed at 4 months; ethnicity not provided; middle class sample | Full-term, birth weight > 2500 g, Apgar > 8 at 5 min, no congenital abnormalities; uncomplicated prenatal, perinatal, and neonatal courses | Italy |
| Schmidt et al 2003 | Longitudinal Laboratory | Infants assessed 3 (n=33), 6 (n=42), 9 (n=52), and 12 (n=40) months (gender NA); primarily Caucasian; SES not provided | recruited from a large database that contained the birth records | Canada |
| Skarin 1977 | Cross-Section Laboratory and Home | 32 infants (16 girls) assessed at 5-7 months or 10-12 months; 100% Caucasian; middle class sample | white, middle class families | USA |
| Waters et al 1975 | Cross-Section Laboratory | 26 infants assessed at 5 (12 girls) and 7 (14 girls) months; 100% Caucasian; middle class sample | white middle class family from pool of volunteers | USA |
| Zeegers et al 2017 | Longitudinal Laboratory | 135 infants (75 girls) assessed at 4 and 12 months; primarily Caucasian; SES not provided | excluded if infant’s birth weight under 2500 g, infant had neurological disorders, or APGAR score < 8 | Netherlands |
| **Between 6 and 12 months of age (n = 33)** | | | | |
| Anderson et al 1999 | Longitudinal Laboratory | 45 infants (22 girls) assessed at 5 and 10 months; ethnicity not provided; SES not provided | Healthy, full-term, first-born, single born | Sweden |
| Baker et al 2012 | Longitudinal Laboratory | 70 toddlers (34 girls) assessed at 12, 24, and 36 months; ethnicity not provided; SES not provided | NA | England |
| Bohlin & Hagekull 1993 | Cross-Section Laboratory | 31 infants (19 girls) assessed at 10-13 months; ethnicity not provided; SES not provided | NA | Sweden |
| Brooker et al 2013 | Cross-Section Laboratory | 88 infants (gender NA) assessed at 6 months; over 90% sample Caucasian; middle class sample | All twins | USA |
| Bush et al 2017 | Cross-Section Laboratory | 135 toddlers (66 girls) assessed at 6 months; 85% self-report as ethnic or racial minority; low to middle class sample | Singleton pregnancy, low-middle income, without medical conditions affecting gestational weight gain | USA |
| Busuito et al 2017 | Cross-Section Laboratory | 53 infants (20 girls) assessed at 6 months; 53% European American; low- and middle-class sample | Married, cohabitating, or had weekly contact with the infants’ fathers | USA |
| Busuito et al 2019 | Cross-Section Laboratory | 140 infants (60 girls) assessed at 6 months; 75% Caucasian; SES not provided | Low-risk, non-smoking women carrying singleton pregnancies | USA |
| Campos et al 1975 | Longitudinal Laboratory | 80 infants, 40 (22 girls) assessed at 5 months and 40 (11 girls) assessed at 9 months; ethnicity not provided; SES not provided | NA | USA |
| Eiden et al 2018 | Cross-Section Laboratory | 77 infants (36 girls) assessed at 9 months; 82.6% non-Caucasian; low- to -middle class sample | Non-smoking women | USA |
| Feldman et al 2010 | Cross-Section Laboratory | 53 infants (28 girls) assessed at 6 months; ethnicity not provided; SES not provided | Singleton healthy infant, mother married/cohabitating with infant’s father, mother at least high-school education and over age 21. | Israel |
| Fracasso et al 1994 | Longitudinal Laboratory | 58 infants (32 girls) assessed at 5 months, 53 (29 girls) at 7 months, 44 (25 girls) at 10 months, and 49 (24 girls) at 13 months; 100% Caucasian; upper-middle class sample | First-born, Caucasian from upper-middle-class, two-parent families | USA |
| Hay et al 2017 | Cross-Section Laboratory | 275 infants (124 girls) assessed at 12 months; 93% Caucasian; middle class sample | NA | UK |
| Holochwost et al 2014 | Cross-Section Laboratory | 95 infants (50 girls) assessed at 6 months; 54% African American, 46% European American; SES sampled from low- and high-income groups | NA | USA |
| Johnson et al 2014 | Cross-Section Laboratory | 41 infants (17 girls) assessed at 6 months; 54% African American, 46% European American; SES sampled from low- and high-income groups | No Axis I disorder; excluded for maternal diagnosis of substance abuse/dependence during pregnancy, and major congenital malformations/disorders | USA |
| Mireault et al 2018 | Longitudinal Home | Two groups: (1) 37 infants (18 girls) assessed at 5, 6, and 7 months; ethnicity not provided; middle class sample; (2) 46 infants (27 girls) assessed at 4, 6, and 8 months; ethnicity not provided; middle class sample | Infants were full-term | USA |
| Moore 2009 | Cross-Section Laboratory | 48 infants (19 girls) assessed at 6 months; 52% European American, 45% African American; 31% lower class, 69% middle class sample | Full-term, healthy infants; married or cohabiting mothers | USA |
| Moore et al 2009 | Cross-Section Laboratory | 152 infants (74 girls) assessed at 6 months; 57% African American, 43% European American; 52% lower class, 48% middle class sample | Drawn from larger study - had to have 6-month ax data | USA |
| Morasch et al 2012 | Longitudinal Laboratory | 106 infants (56 girls) assessed at 5 and 10 months; 88% Caucasian, 5% Hispanic, 7% other; SES not provided | Born within two weeks of due date and no neurological or developmental problems | USA |
| Noten et al., 2019b | Cross-Section Laboratory | 126 infants (57 girls) assessed at 6 months, 13 days; 85% Caucasian; SES not provided | Dutch speaking mothers between 17 and 25 years without birth complication | Netherlands |
| Perry et al 2016 | Cross-Section Laboratory | 230 infants (124 girls) assessed at 5 or 10 months; 78% Caucasian, 13% African American, 9% other; SES not provided | Born within 15 days of calculated due dates and healthy at the time of testing | USA |
| Provost et al 1979 | Cross-Section Laboratory | 40 infants (20 girls) assessed at 9 or 12 months; ethnicity not provided; SES not provided | NA | Canada |
| Qu et al 2018 | Longitudinal Laboratory | 206 infants (gender NA) assessed at 6 and 14 months; 49% Caucasian, 51% African American; middle class sample | From larger study | USA |
| Rash et al 2015 | Cross-Section Laboratory | 194 infants (gender NA) assessed at 6 months; ethnicity not provided; middle and upper-middle class sample | Women excluded if non-singleton pregnancy, using steroid medication, smoking, consuming alcohol or illicit drugs, or pregnancy or fetal complications | Canada |
| Rash et al 2016 | Cross-Section Laboratory | 254 infants (120 girls) assessed at 6 months; 77.3% Caucasian; middle class sample | Women excluded if non-singleton pregnancy, using steroid medication, smoking, consuming alcohol or illicit drugs, or pregnancy or fetal complications | Canada |
| Santesso et al 2007 | Cross-Section Laboratory | 39 infants (15 girls) assessed at 9 months; primarily Caucasian; SES not provided | recruited from a large database that contained the birth records | Canada |
| Schmidt et al 2003 | Longitudinal Laboratory | Infants assessed 3 (n=33), 6 (n=42), 9 (n=52), and 12 (n=40) months (gender NA); primarily Caucasian; SES not provided | recruited from a large database that contained the birth records | Canada |
| Skarin 1977 | Cross-Section Laboratory and Home | 32 infants (16 girls) assessed at 5-7 months or 10-12 months; 100% Caucasian; 100% middle class sample | white, middle class families | USA |
| Spangler et al 1993 | Cross-Section Laboratory | 41 infants (17 girls) assessed at 12 months; 100% Caucasian; 27% lower class, 24% middle class, 49% upper middle class | NA | Germany |
| Stone et al 2013 | Cross-Section Laboratory | 101 infants (53 girls) assessed at 6 months; 94% Caucasian, 5% Hispanic, 1% Asian; middle class sample | healthy, full-term with no pregnancy, birth, or perinatal complications | USA |
| Vaughn et al 1979 | Cross-Section Laboratory | 16 infants (gender NA) assessed at 8-16 months; 100% Caucasian; middle class sample | Artifact free HR data and had to cry during assessment | USA |
| Waters et al 1975 | Cross-Section Laboratory | 26 infants assessed at 5 (12 girls) and 7 (14 girls) months; 100% Caucasian; middle class sample | white middle class family from pool of volunteers | USA |
| Weinberg et al 1996 | Cross-Section Laboratory | 50 infants (25 girls) assessed at 6 months; 100% Caucasian; middle class sample | randomly selected from larger longitudinal study | USA |
| Zeegers et al 2017 | Longitudinal Laboratory | 135 infants (75 girls) assessed at 4 and 12 months; primarily Caucasian; SES not provided | excluded if infant’s birth weight under 2500 g, infant had neurological disorders, or APGAR score < 8 | Netherlands |
| **Between 13 and 24 months of age (n = 16)** | | | | |
| Baker et al 2012 | Longitudinal Laboratory | 70 toddlers (34 girls) assessed at 12, 24, and 36 months; ethnicity not provided; SES not provided | NA | England |
| Bohlin & Hagekull 1993 | Cross-Section Laboratory | 31 infants (19 girls) assessed at 10-13 months; ethnicity not provided; SES not provided | NA | Sweden |
| Buss et al 2004 | Cross-Section Laboratory | 80 toddlers (39 girls) assessed at 24 months; 95% Caucasian; middle class sample | Not selected for any behavior or characteristic | USA |
| Buss et al 2005 | Cross-Section Laboratory | 68 toddlers (33 girls) assessed at 24 months; 94% Caucasian; middle class sample | Not selected for any behavior or characteristic | USA |
| Calkins et al. 1992 | Longitudinal Laboratory | 50 infants (25 girls) assessed at 5 months, 52 toddlers (27 girls) assessed at 14 months, and 48 (25 girls) assessed at 24 months; 92% Caucasian; middle class sample | Gestational age 38-42 weeks, birth weight 2,500-4,090g, Apgar scores >7 at 1 min and 8 at 5 min, and normal pediatric examination | USA |
| Calkins et al., 1998a | Cross-Section Laboratory | 65 toddlers (35 girls) assessed at 24 months; 88% Caucasian; middle class sample | Excluded if prenatal/perinatal complications, gestational age < 38 weeks or child’s birthweight < 5 lbs | USA |
| Calkins et al. 1998b | Cross-Section Laboratory | 73 toddlers (41 girls) assessed at 18 months; 89% Caucasian; middle class sample | Excluded if any prenatal/perinatal complications or gestational age < 38 weeks | USA |
| Calkins et al. 2000 | Cross-Section Laboratory | 50 toddlers (25 girls) assessed at 24 months; 60% European American, 30% African American, 5% other; 25% lower class, 61% middle class, 14% upper class | NA | USA |
| Cho et al 2017 | Cross-Section Laboratory | 62 toddlers (gender NA) assessed at 24 months; 90% Caucasian; middle class sample | NA | USA |
| Dawson et al 2001 | Cross-Section Laboratory | 159 infants (89 girls) assessed at 13–15-months; 69 classified as non-depressed and included here; 85% Caucasian; middle class sample | no substance use/abuse, serious medical or mental health concerns, significant pregnancy/birth complications; Infants full term with no complications | USA |
| Eisenberg et al. 2012 | Cross-Section Laboratory | 213 toddlers (94 girls) assessed at 18 months; 84% Caucasian; middle class sample | Mothers 18 years of age or older, read English fluently, and have infants born full term and without birth complications | USA |
| Fracasso et al 1994 | Longitudinal Laboratory | 58 infants (32 girls) assessed at 5 months, 53 (29 girls) at 7 months, 44 (25 girls) at 10 months, and 49 (24 girls) at 13 months; 100% Caucasian; upper-middle class sample | First-born, Caucasian from upper-middle-class, two-parent families | USA |
| Hill-Soderlund et al 2008 | Cross-Section Laboratory | 132 infants (67 girls) assessed at 14 months; 56% African American, 44% European American; 51% low income sample, 49% middle class sample | Equal number of European American and African American families from lower- and higher-income groups | USA |
| Liew et al 2011 | Longitudinal Laboratory | 247 children (gender NA) assessed at 18 and 30 months; 72% Caucasian, 11% Hispanic, 17% other; middle class sample | Mothers and fathers at least 18 years old with healthy, full-term infants born without complications | USA |
| Qu et al 2018 | Longitudinal Laboratory | 206 infants (gender NA) assessed at 6 and 14 months; 49% Caucasian, 51% African American; middle class sample | From larger study | USA |
| Vaughn et al 1979 | Cross-Section Laboratory | 16 infants (gender NA) assessed at 8-16 months; 100% Caucasian; middle class sample | Artifact free HR data and had to cry during assessment | USA |
| **Between 2 and 3 years 11 months of age (n = 10)** | | | | |
| Baker et al 2012 | Longitudinal Laboratory | 70 toddlers (34 girls) assessed at 12, 24, and 36 months; ethnicity not provided; SES not provided | NA | England |
| Blankson et al 2012 | Cross-Section Laboratory | 263 children (gender NA) assessed at 40 months; 58% European American, 35% African American; 7% other ethnicities; middle class sample | NA | USA |
| Calkins 1997 | Cross-Section Laboratory | 41 toddlers (20 girls) assessed at 2-3 years;82% Caucasian; middle class sample | NA | USA |
| Calkins et al. 2004 | Cross-Section Laboratory | 154 toddlers (76 girls) assessed at ages 2 and 4.5 years; 65% European American; low- and middle-class | Sample diverse in socioeconomic status (SES) and ethnicity | USA |
| Gilissen et al 2007 | Cross-Section Laboratory | 78 toddlers (40 girls) assessed at 3-4 years; 100% Caucasian; SES not provided | NA | Netherlands |
| Liew et al 2011 | Longitudinal Laboratory | 247 children (gender NA) assessed at 18 and 30 months; 72% Caucasian, 11% Hispanic, 17% other; middle class sample | Mothers and fathers at least 18 years old with healthy, full-term infants born without complications | USA |
| Paret et al 2015 | Cross-Section Laboratory | 48 children (25 girls) assessed at 3-4 years; majority Caucasian; elevated SES sample | NA | Canada |
| Perry et al 2012 | Cross-Section Laboratory | 197 children (98 girls) assessed at 3-4 years; 61% Caucasian; 37% lower class, 50% middle class, 13% upper class sample | Efforts made to recruit equal number of male and female participants from economically and racially diverse backgrounds | USA |
| Scrimgeour et al 2016 | Cross-Section Laboratory | 125 children (62 girls) assessed at 3.5 years; 90% Caucasian; middle class sample | from larger prospective study | USA |
| Wagner et al. 2018a | Cross-Section Laboratory | 108 children (54 girls) assessed at 2 years; 97% Caucasian; SES not provided | from longitudinal study of children’s socio-emotional development | USA |
| **Between 4 and 4 years 11 months of age (n = 5)** | | | | |
| Calkins et al. 2004 | Cross-Section Laboratory | 154 toddlers (76 girls) assessed at ages 2 and 4.5 years; 65% European American; low- and middle-class | Sample diverse in socioeconomic status (SES) and ethnicity | USA |
| Gilissen et al 2008 | Cross-Section Laboratory | 78 preschoolers (40 girls) assessed at 4 years; ethnicity not provided; SES not provided | NA | Netherlands |
| Noten et al., 2019a | Cross-Section Laboratory | 61 preschoolers (33 boys) assessed at 4 years; 85% Caucasian; SES not provided | NA | Netherlands |
| Wagner et al. 2018b | Cross-Section Laboratory | 88 children (42 girls) assessed at 4 years; 97% Caucasian; SES not provided | drawn from a study of children’s socioemotional development | USA |
| Zeytinoglu et al., 2019 | Cross-Section Laboratory | 278 children (153 gilts) assessed at 4 years; 59% European American, 30% African American, 11% other; middle class sample | drawn from a study of children’s early academic readiness | USA |
