# Supplementary material for: Physiological measurement of emotion from infancy to preschool: A systematic review and meta‐analysis
Source: Brain Behav. 2020 Dec 17;11(2):e01989. doi: 10.1002/brb3.1989 (PMC7882167; doi:10.1002/brb3.1989)
Supplement: Supplementary file 3 — Table S3 [file BRB3-11-e01989-s003.docx]

| Table 3. Descriptions of Emotional-Evoking Tasks | | | | |
| --- | --- | --- | --- | --- |
| **Probed Emotion** | **Task** | **Description** | **Duration** | **Article Using Task** |
| Anger | Narrated Comic Strip | Children watched narrated comic strip film which presented multiple emotionally salient stories; Two anger‐inducing portions of video depicted child expressing anger toward antagonistic peer and child having heated argument with her/his mother; Both scenarios were accompanied by dramatic music and had positive resolution | Two 60 second clips | Wagner et al 2018b |
| Disappointment | Disappointment Task | Three phases: (1) Waiting for Gift - experimenter announced that prize had been earned following other task completion; experimenter left room and returned with wrapped box containing prize that had been ranked as least desired; (2) Wrong Gift - experimenter maintained neutral expression and detached demeanor as undesirable gift was opened, remained in the room, and then exited. Sixty seconds later, second experimenter entered, feigned surprise and asked how child felt when received least-desired prize; (3) Resolution - experimenter re-entered the room, explained the mix-up with prizes and gave child the opportunity to exchange the prize | Waiting for Gift 30 seconds; Wrong Gift 40 seconds; Resolution 60 seconds | Scrimgeour et al 2016; |
| Distress | Arm Restraint | Caregiver (Calkins 1992) or Researcher (Johnson 2014) hold child’s arms at their side (discontinued if child cries) | Each trial 120 seconds | Calkins et al 1992; Johnson et al 2014; Perry et al 2016 |
| Distress | Arm Restraint - Modified 1 | Mother stood behind child and gently grasped child forearms and firmly hold them to their side while an attractive toy was placed directly in front of child; Recovery period followed a second trial with child playing with toy or comforted by parent | Arm restraint 30 seconds; Recovery 15 seconds | Rash et al 2015; 2016 |
| Distress | Arm Restraint - Modified 2 | Mothers instructed to gently hold child’s arms to their sides of the child and then to release the arms while maintaining a still face with no verbal interactions. | Both trials 90 seconds | Stone et al 2013 |
| Distress | Arm Restraint - Modified 3 | During toy removal task, mother engaged her child in play with an interesting toy; mother then held toy out of child’s reach, retaining eye contact but silent with still face. Mother next gently restrained her child’s arms against his/her sides while maintaining a still face and silence. | Toy play 30 seconds; toy removal and arm restraint 120 seconds | Morasch et al 2012 |
| Distress | Arm Restraint and Toy Play | Child was first presented and encouraged to play with an attractive toy; experimenter stood behind child, placed hands on the child's forearms and moved them to child's sides and held them while maintaining a neutral expression; After first trial, child was allowed to play with the toy again followed by a second arm restraint. The child was again allowed to play with the toy after arm restraint | Toy presentation and Arm restraint each 30 seconds | Eiden et al 2018 |
| Distress | Distressing Audio or Video | Child looked at books while audiotape of a crying toddler was played just outside the playroom door (at age 2) or child was shown a videotape in which a young child experiences the death of a pet (age 4) | Audio was 120 seconds; Video was 240 seconds | Calkins et al 2000; 2004 |
| Distress | Still Face Paradigm | During Play, parent plays with child without toys. During Still-Face, parent maintains a neutral expression and does not touch or interact with child. During Reunion, there is a resumption of play with parent responding to child; Feldman et al (2010) had a 3-minute Play; Gray et al (2017) had 2.5-minute episodes for each phase | Each episode 120 seconds | Bush et al 2017; Busuito et al 2017; 2019; Gray et al 2017; Ham et al 2006; Holochwost et al 2014; Moore et al 2004; Moore 2009; Moore et al 2009; Provenzi et al 2015; Qu et al 2018; Weinberg et al 1996; Noten et al., 2019b |
| Distress | Still-Face Paradigm Modified | Second Still-Face and Reunion added. During Play, parent played with child and were given a toy. Parents did not touch their child during the procedure. During Still-Face, parents expressed a neutral expression, remained still, and looked slightly above child’s head to avoid eye contact. During Reunion, there is a resumption of play with parent responding to child | Each episode 120 seconds | Haley et al 2003; |
| Distress | Still-Face with Touch | During Play, parent plays with child without toys. During Still-Face, parent maintains a neutral expression and does not touch or interact with child. Mother may be asked to provide tactile contact during Still-Face. During Reunion, there is a resumption of play with parent responding to child | Each episode 180 seconds | Feldman et al 2010 |
| Distress | Still-Face with Touch or Arm Restraint | During Play, parent plays with child without toys. During Still-Face, parent maintains a neutral expression and does not touch or interact with child. Mother may be asked to provide tactile contact or restrain arms of child during Still-Face. During Reunion, there is a resumption of play with parent responding to child | Play 180 seconds; Still Face and Reunion 120 seconds | Pratt et al 2015 |
| Distress | Strange Situation Modified | Play with parent, followed by a brief separation from mother, and then a reunion | Play 600 seconds; Separation 180 seconds; Reunion 300 seconds | Calkins et al 1992 |
| Distress | Teddy Bear Picnic | Two costumed characters (the “Birthday Lady” and the “Teddy Bear”): Birthday Lady encouraged children to sit around picnic mat and play with plastic food and parents return to sofa in room. The Teddy Bear entered, pausing in doorway until each child had seen him. Birthday Lady invited Teddy Bear to sit down near picnic blanket, and he offered each child a piece of plastic birthday cake. Birthday Lady and Bear then danced while singing “Round and Round the Garden” before offering each child, with the help of their parents, the opportunity to dance with Bear. Birthday Lady instructed families to let children play in any way they would like and then she left the room. . | NA | Hay et al 2017 |
| Distress | Videos - Crying | Children seated in high chair with mother’s present; video depicted babies crying | Clip 42 seconds | Eisenberg et al. 2012; Liew et al 2011 |
| Fear | Interesting but Scary | Mothers opened cupboard containing witch-like mask with speaker inserted behind it; Mother shown mask prior and asked to act as normally would if child became frightened; Experimenter, from another room, engaged child in conversation (via the mask) in a friendly voice. First, examiner asked child about toys in which he or she had played then invited child to touch her nose (the mask) | 120 seconds | Paret et al 2015 |
| Fear | Multiple Stranger Approach | Using four different combinations, Stranger 1 enters and departs, then Stranger 2 enters and departs. Mother departs and Mother re-enters | Four 15 second phases | Campos et al 1975 |
| Fear | Spider | Experimenter presented child with large, realistic, moving spider and encouraged child to touch it | 120 seconds | Calkins et al 2000 |
| Fear | Strange Situation - Ainsworth | Child seated in high chair and completed seven episodes: mother and child together, stranger enters room with mother and child, mother leaves stranger and child alone, stranger leaves and mother and child together, mother leaves and child is alone, stranger re-enters with child, and stranger leaves and mother returns with child. | NA | Hill-Soderlund et al 2008; Spangler et al 1993 |
| Fear | Stranger Approach Modified | Child seated and affectively neutral, male stranger entered; Stranger paused near door before approaching half of distance toward child; Stranger then paused, addressed child, approached rest of the distance to child, and knelt near him/her for period of time; At the end of this period, stranger rose and exited room | Knelt 120 seconds | Brooker et al 2013; Buss et al 2004; 2005; Skarin 1997 |
| Fear | Stranger Approach Modified 3 | Mothers seated on chair behind child; During task, male stranger approached and talked to the child and then picked up and held child | Talking 30 seconds; Holding 30 seconds | Zeegers et al 2017 |
| Fear | Stranger Challenge | Male stranger entered room and stared at child without speaking then left the room | Episode each 60 seconds | Wagner et al 2018a |
| Fear | Stranger Wariness | Mother put child in high-chair; Female stranger entered room and approached child in a standard stepwise fashion consisting of six episodes (pause and call child’s name 1, pause and call child’s name 2, approach, pause at a distance of 1 meter, reach out to child, and touch child); Stranger then left room without any comments | Six episodes each 5 seconds (30 seconds total) | Anderson et al 1999; Bohlin & Hagekull, 1993; Waters et al., 1975 |
| Fear | Unpredictable Mechanical Toy | Mother leaves room and unfamiliar experimenter enters and placed robot 1.5 m away from child; Experimenter makes robot approach child, stopping 15 cm from child, while making movements with its arms and emitting noise. The robot then walks backward and stops at back of room for 10 s before moving forward again; This was repeated three times | NA | Baker et al 2012 |
| Fear | Video | Episodes contained one neutral and one fear-inducing video clip; Neutral clips were from a videotape (Tik Tak 15), which showed colorful moving shapes; Fear-inducing clips chosen from movie ‘Dinosaur,’ (Walt Disney); Children watched two episodes; one with parent and one alone; Clips were counterbalanced | Each clip 60 seconds | Gilissen et al 2007; 2008 |
| Frustration | Food Denial | Experimenter entered room and placed sealed crackers (in a plastic bag) on sofa and told child that crackers could not be eaten until play was finished | 120 seconds | Calkins et al 1998b; 2000 |
| Frustration | Frustrating Puzzle Task | Child given a wood toy with many holes with string laced through the holes (middle of string was glued to inside of toy, making it impossible to untangle completely). Experimenter asked child to untangle toy while he/she worked on paperwork in other room. The experimenter left the room and upon return, experimenter presented second unglued puzzle to child and allowed child to completely unlace string and solve puzzle | 180 seconds | Perry et al 2012 |
| Frustration | Green Circles | Experimenter repeatedly asks child to draw circles with green marker. Experimenter criticizes child’s circles but does not say how to do better. Experimenter continues to prompt, ‘I need the perfect green circle’ for the duration of the task | 240 seconds | Blankson et al 2012 |
| Frustration | High-Chair | Experimenter placed child in high chair and told child to wait for a special toy; mother sat nearby with magazine and responded normally to child if child spoke to her but did not remove child from chair | 300 seconds | Calkins et al 1998b |
| Frustration | Locked Box with Snack | Experimenter placed clear plastic container of cookies on table that child is unable to open and left room; Child was free to manipulate container while experimenter was gone; Mother was instructed not to open container | 120 seconds | Calkins et al. 2004 (@ 2 years) |
| Frustration | Locked Box with Toy | Child offered choice of two highly desirable toys; after child makes selection, toy is placed in transparent box that is locked with a padlock. After showing child how to open lock with key, experimenter gave child large ring of keys, none of which was the correct key, and told child to open box to get toy. Experimenter leaves room while child attempts to open box and re-enters to present child with correct key. Child then opens box and plays with toy | 240 seconds | Blankson et al 2012; Calkins et al. 2004 (@ 4.5 years); Zeytinoglu et al., 2019 |
| Frustration | Plexiglass Barrier | Experimenter and child play with musical telephone; After play, experimenter took toy away and placed it behind Plexiglas barrier out of child's reach | Play 60 seconds; Removal 120 seconds | Calkins et al 1998b |
| Frustration | Plexiglass Barrier 2 | Child engaged with stuffed rabbit. After play, experimenter placed plexiglass barrier in front of child and asked parent to remove toy from child and place it behind barrier; Toy remained behind barrier and then child was allowed to play with it again; repeated two additional times | Play 15 seconds; Removal 30 seconds | Rash et al 2015; 2016 |
| Frustration | Toy Removal 1 | After child played with toy for a few minutes, parent takes toy away saying, ‘‘I don’t want you to play with this anymore.’’ For task, mother stands up and places toy on shelf directly in front of child and sits back down in her chair; toy then returned to child | Removal 30 seconds; Returned 60 seconds | Buss et al 2005; Calkins et al. 1998a; 1998b |
| Frustration | Toy Removal 2 | Experimenter takes toy away from child and plays with it for 2 minutes, commenting on how fun it is to play with | 120 seconds | Zeytinoglu et al., 2019 |
| Frustration | Toy Removal in Box | Experimenter gave child attractive electronic musical toy to play with; Experimenter then tasked toy away, placed it in clear plastic box that child was not able to open, and put box on the table in front of child | Play 60 seconds; Removal 120 seconds | Calkins 1997 |
| Frustration | Toy Retraction | Child played with novel toy, after which mother removed toy from child’s reach; Toy then returned to the child; repeated two additional times | Play 15 seconds; Removal 30 seconds | Rash et al 2015; 2016 |
| Frustration | Car Seat Task | Child was bucked into car seat and mother stood behind child (who the child could see if they turned their head) | 60 seconds | Noten et al., 2019b |
| Guilt | Mishap Guilt Paradigm | Child presented with tower, which experimenter says is her favorite toy and had made it herself; She told child that she would share it as long as they were very careful. Because tower is rigged, it fell apart as soon as child began to handle it; Experimenter then says “Oh my” with regret and sits still in front of child with her face covered with her hands She asked, “What happened?”, “Who did it?”, and “Did you do it?”; Child is told that it was not their fault and there was a problem with tower. She gives partially built tower to child and asks child to help her make it; Experimenter tells child damage was not their fault and assumed responsibility for it | Face covering 30 seconds; Other parts NA | Baker et al 2012 |
| Positive | Absurd Event | Research assistant showed parents two ordinary events (narration of playing with a ball/drinking from a cup and read a book) and two absurd events (ball worn as a clown nose and continuously poked while saying ‘beep’ and book/cup worn like hat and continuously raised and lowered while saying ‘zoop’). Each absurd event was presented twice, once with parents holding neutral affect and once with positive affect (i.e., smiling and laughing). Parental affect was not manipulated during ordinary events | Each event 45 seconds | Mireault et al 2018 |
| Positive | Peek-a-Boo Play | Mother played peek-a-boo with child and familiar experimenter played peek-a-boo | Each session 60 seconds | Dawson et al 2001 |
| Positive | Puppet Play | Game of peek-a-boo with a puppet named Spot | NA | Calkins et al 1998a; 2000; Calkins 1997; Cho et al 2017 |
| Positive, Angry, Neutral | Emotion Evoking Task | Mothers instructed to turn towards experimenter while experimenter enacted script directed towards them in angry, excited, or neutral tone of voice; Same script used for each emotion; Mothers were instructed not to respond in any way | Episodes each 60 seconds | Moore 2009 |
| Positive, Fear | Audio - ID speech | Mothers expressed either comfort, surprise, or fear as they said ‘‘Hey, honey, come over here’’ to their children; Conditions were constructed such that samples expressing that emotion were played in random order | Episode each 60 seconds with 30 second inter-trial pause | Santesso et al 2007 |
| Positive, Fear | Peek-a-boo then scary mask | Child and mother played peek a boo; After child was positively engaged, mother called child name and appeared from behind screen wearing a full-face mask; She then returned behind screen and repeated procedure; On second trial, stranger wore mask, after which, mask was removed and stranger approached child | NA | Vaughn 1979 |
| Positive, Fear, Frustration | Strange Situation and Toy Box | Six episodes: (1) exploration - child explores with mom in room, (2) play with mom - mom shows child how pull toy works, (3) frustration - mom puts toys in box and restrains child on his back, (4) reaction to stranger - experimenter approaches child, (5) isolation - child is left alone in room, and (6) reunion - mom comes back in with child and shows child how the pull toy works | Episodes each 180 seconds | Provost et al 1979 |
| Positive, Fear, Sadness | Musical Pieces | Three orchestra excerpts that are known to vary in affective valence and intensity: Adagio by Barber reflected sadness; Peter and the Wolf by Prokofiev reflected fear; and Spring by Vivaldi (second movement) reflected joy | Pieces each 30 seconds | Schmidt et al 2003 |
| Positive, Negative | Videos | Children placed in seat and shown variety of stimuli designed to elicit both positive and negative emotions | 20 minutes (7, 10 months); 30 minutes (13 months) | Fracasso et al 1994 |
| Positive, Neutral | Smiling/Blank Face | Female experimenter sat facing child and completed two trials: (1) Smiling Face - experimenter smiled while looking at child without moving her head, touching, or speaking to engage child, (2) Blank Face - experimenter stopped smiling and held a blank face while looking at child (discontinued if child became distressed) | Smiling Face 30 seconds; Blank Face 50-130 seconds | Bazhenova et al 2007 |
| Positive, Sadness, Fear | Videos | Children watched three different videos (presented in random order); happy (a happy child opening a gift), fear (a child being scared by a toy spider), and sadness (a sad child flushing his dead goldfish down the toilet) | Each video clip was 50 seconds | Noten et al., 2019a |
