# Supplementary material for: Physiological measurement of emotion from infancy to preschool: A systematic review and meta‐analysis
Source: Brain Behav. 2020 Dec 17;11(2):e01989. doi: 10.1002/brb3.1989 (PMC7882167; doi:10.1002/brb3.1989)
Supplement: Supplementary file 4 — Table S4 [file BRB3-11-e01989-s004.docx]

| Table 4. Baseline Tasks Characteristics | | | | | |
| --- | --- | --- | --- | --- | --- |
| **Article** | **Sample Size, Age Assessed** | **Baseline Description** | **Length (Epochs)** | **Measurement** | **Mean (SD)** |
| **Unknown Task (n=5)** | | | | | |
| Hay et al., 2017 | n=275, 12m | "Baseline period" | 180 seconds (15 second epochs) | Mean HR | 132.81 (21.04) bpm |
| Santesso et al., 2007 | n=39, 9m | "Baseline period" | 60 seconds (na) | Mean HP (to HR) | 138 (NA) bpm |
| Schmidt et al., 2003 | n=33 to 52 at 3, 6, 9, and 12m | "Baseline period" | 60 seconds (na) | Mean HP (to HR) | 3m: 147 (15.53) bpm; 6m: 144 (11.74) bpm; 9m: 136 (11.42) bpm; 12m: 131 (11.56) bpm |
| Stone et al., 2013 | n=101, 6m | "Baseline period" | 180 seconds (na) | Mean HP and RSA | Bradycardia: 154 (12.08) bpm; Non-Bradycardia: 151 (11.88) bpm |
| Zeegers et al., 2017 | n=135, 4 and 12m | "Baseline period" | 120 seconds (na) | Mean HRV | 4m: 16.57 (9.85) msec; 12m: 29.88 (16.75) msec |
| **Sitting Quietly (n=12)** | | | | | |
| Busuito et al., 2017 | n=53, 6m | Infants sat quietly in chair across from mothers while mother read description of study | 180 seconds (30 second epochs) | Mean RSA | 3.65 (1.07) In(msec2) |
| Calkins et al., 1992 | n=50, 5m; n=52, 14m; n=48; 24m | Infant sat in mothers lap | 300 seconds (na) | Mean HP (to HR) and RSA | NA |
| Campos et al., 1975 | n=80, 5m; n=40, 9 | Resting period | 240-300 seconds (3 second epochs) | Mean HR | NA |
| Fracasso et al., 1994 | n=58, 5m; n=53, 7m; n=44, 10m; n=49, 13m | Infants sat on mothers’ laps in awake, quiet, and attentive state | 300 seconds (30 second epochs) | Mean HP (to HR) and RSA | 5m: 142 (9.76) bpm; 7m: 138 (9.90) bpm; 10m: 135 (10.30) bpm; 13m: 136 (10.80) bpm; 5m: 3.02 (.71) In(msec2); 7m: 3.25 (.72) In(msec2); 10m: 3.27 (.72) In(msec2); 13m: 3.20 (.71) In(msec2) |
| Holochwost et al., 2014 | n=95, 6m | Child seated facing away from mother on her lap | 120-240 seconds (15 second epochs) | Mean RSA | 3.72 (.91) In(msec2) |
| Johnson et al., 2014 | n=41, 6m | Infant in car seat in quiet, low-lit room while mother sat behind occlusion screen | 15 seconds (na) | Mean RSA | 3.58 (1.76) In(msec2) |
| Moore, 2009 | n=48, 6m | Sit quietly | 180 seconds (30 second epochs) | Mean RSA | NA |
| Moore et al., 2004 | n=72, 3m | Minimize stimulation, infants not receiving attention from mothers or toys | 180 seconds (30 second epochs) | Hean HR and RSA | 146 (10.89) bpm; 2.82 (.75) In(msec2) |
| Moore et al., 2009 | n=152, 6m | Minimize stimulation, infants not receiving attention from mothers or toys | 120 seconds (15 second epochs) | Mean HP (to HR) and RSA | 3.68 (.85) In(msec2) |
| Rash et al., 2015 | n=194, 6m | Seated on mother’s lap, with movement/interaction at a minimum. | 180 seconds (45 second epochs) | Mean RSA | 461.93 (425.52) msec2/Hz |
| Rash et al., 2016 | n=254, 6m | Seated on mother’s lap, with movement/interaction at a minimum. | 180 seconds (45 second epochs) | Mean RSA | 495.31 (402.72) msec2/Hz |
| Vaughn et al., 1979 | n=16, 8-16m | Seated in high chair | 180 seconds (na) | Mean HR | 130 (NA) bpm |
| **Sedentary Task (n=5)** | | | | | |
| Busuito et al., 2019 | n=140, 6m | Infants seated in mother’s lap, mother’s asked to show/read book to infants. | 420 seconds (30 second epochs) | Mean HP (to HR) and RSA | 138 (9.79) bpm; 3.18 (0.85) In(msec2) |
| Cho et al., 2017 | n=62, 24m | Engaged in quiet, sedentary activities (e.g., coloring) with experimenter. | 300 seconds (30 second epochs) | Mean RSA | 4.46(1.07) In(msec2) |
| Dawson et al., 2001 | n=159, 13-15m | Experimenter blew soap bubbles from behind black curtain | 60 seconds (na) | Mean HR | 128 (NA) bpm |
| Perry et al., 2016 | n=230, 5 or 10m | Seated with mother, watching RA manipulate toy with brightly colored balls | 60 seconds (na) | Mean RSA | 5m: 3.88 (1.18) In(msec2); 10m: 4.61 (1.10) In(msec2) |
| Scrimgeour et al., 2016 | n=125, 42m | Children sat quietly while coloring or reading a book with experimenter | NA (30 second epochs) | Mean RSA | NA |
| **Face-to-Face Episode/Play (n=15)** | | | | | |
| Bush et al., 2017 | n-135, 6m | Free play episode | 120 seconds (30 second epochs) | Mean RSA | 4.27 (1.04) In(msec2) |
| Baker et al., 2012 | n=70, 12, 24, and 36m | Mother and child quietly playing together | 210 seconds (30 second epochs) | Mean HR | 12m: 136.43 (12.15) bpm; 24m: 121.86(9.74) bpm; 36m: 113.72 (9.84) bpm |
| Bazhenova et al., 2007 | n=16, 4m | Mother and child quietly playing together | 210 seconds (30 second epochs) | Mean HP (to HR) and RSA | 155 (7.75) bpm; 3.2 (0.3) In(msec2) |
| Feldman et al., 2010 | n=53, 6m | Free play episode | 120 seconds (na) | Mean RSA | Touch: 3.56 (.85) In(msec2); No Touch 3.65 (.74) In(msec2) |
| Gray et al., 2017 | n=167, 4m | Free play episode | 120 seconds (na) | Mean RSA | 2.7 (.47) In(msec2) |
| Haley et al., 2003 | n=43, 5m | Free play episode | 120 seconds (na) | Mean HR | 146.60(11.92) bpm |
| Ham et al., 2006 | n=12, 5m | Free play episode | 120 seconds (na) | Mean HR and RSA | Recovered: 145 bpm, 3.3 [units unknown]; Stable: 142 bpm, 3.7 [units unknown]; Dysregulated: 137 bpm, 3.5 [units unknown]; Protest: 152 bpm, 3.5 [units unknown] |
| Ham et al., 2009 | n=18, 5m | Free play episode | 120 seconds (na) | Mean HR and RSA | 143.57 (9.97) bpm |
| Hill-Soderlund et al., 2008 | n=132, 14m | Free play episode | 180 seconds (30 second epochs) | Mean RSA | 3.70 (1.03) In(msec2) |
| Mireault et al., 2018 | n=37, 5, 6, and 7m; n=46, 4, 6, and 8m | "Ordinary play" | NA (45 second epoch) | Mean HR | 139.4 (12.43) bpm |
| Pratt et al., 2015 | n=122; 5m | Free play episode | 180 seconds (15 second epochs) | Mean RSA | NA |
| Provenzi et al., 2015 | n=94, 4m | Free play episode | 120 seconds (10 second epochs) | Mean RSA | NA |
| Qu et al., 2018 | n=206, 14m | Free play episode | 120 seconds (15 second epochs) | Mean RSA | 3.65 (.99) In(msec2) |
| Spangler et al., 1993 | n=41, 12m | Stranger approach episode 2 | NA (na) | Mean HR | Insecure: 142.3 (18.2) bpm; Disorganized: 134.2 (6.9) bpm; Secure: 140.7(12.0) bpm |
| Weinberg et al., 1996 | n=50, 6m | Free play episode | 120 seconds (10 second epochs) | Mean HR and RSA | 138.20 (NA) bpm, 3.165 (NA) In(msec2) |
| **Before/Between Tasks (n=5)** | | | | | |
| Anderson et al., 1999 | n=45, 5 and 10 m | Episode preceding the entrance of the stranger | 5 seconds | Mean HR | NA |
| Bohlin & Hagekull, 1993 | n=31, 10-13m | Episode preceding the entrance of the stranger | 5 seconds (na) | Mean HR | Mpres: 130.2 bpm; Mabs: 146.8 bpm |
| Provost et al., 1979 | n=40, 9-12m | Immediately before each episode | 15 seconds (5 second epochs) | Mean HR | With Mother: 147.2 bpm; Frustration: 148.5(NA) bpm; Isolation: 147.7 (NA) bpm; Reunion: 156.4 (NA) bpm |
| Skarin, 1977 | n=32, 5-7 or 10-12m | Immediately before each approach step | 5 seconds (500 msec epochs) | Mean HR | NA |
| Waters et al., 1975 | n=26, 5 and 7m | Immediately before task | 10 seconds (na) | Mean HR | 120 (NA) bpm |
| **Watched Video (n=22)** | | | | | |
| Blankson et al., 2012 | n=263, 40m | Watched video (Spot) | 300 seconds (na) | Mean RSA | 6.41 (1.32) In(msec2) |
| Brooker et al., 2013 | n=124, 6m | Watched video (Baby Mugs) | not provided (30 second epochs) | Mean RSA | 3.55 (.77) In(msec2) |
| Buss et al., 2004 | n=80, 24m | Watched video (Baby Mugs) | 300 seconds (30 second epochs) | Mean HR and RSA | 119.03 (8.53) bpm, 5.06 (.95) In(msec2) |
| Buss et al., 2005 | n=68, 24m | Watched video (Baby Mugs) | 300 seconds (30 second epochs) | Mean HP (to HR) and RSA | 119.03 (8.53) bpm, 5.06 (.95) In(msec2) |
| Calkins, 1997 | n=41, 24-36m | Watched video (Spot) | 300 seconds (30 second epochs) | Mean RSA | 5.66 (NA) In(msec2) |
| Calkins et al., 2000 | n=50, 24m | Watched video (Spot) | 300 seconds (30 second epochs) | Mean HP (to HR) and RSA | 5.53 (NA) In(msec2) |
| Calkins et al., 1998b | n=73, 18m | Watched video (Barney) | 300 seconds (30 second epochs) | Mean HP (to HR) and RSA | NA |
| Calkins et al., 2004 | n=154, 24 and 54m | Watched video (Spot) | 300 seconds (30 second epochs) | Mean HP (to HR) and RSA | 24m: 109 (10.89) bpm; 5.76 (1.4) In(msec2); 54m: 97 (12.86) bpm, 5.95 (1.35) In(msec2); |
| Calkins et al., 1998a | n=65, 24m | Watched video (Spot) | 300 seconds (30 second epochs) | Mean RSA | 5.36 (1.22) In(msec2) |
| Eiden et al., 2018 | n=69, 9m | Watched video (Baby Einstein) | 180 seconds (na) | Mean RSA | Boys: 0.016 (.01) sec; Girls: 0.02 (.01) sec |
| Eisenberg et al. 2012 | n=213, 18, 30, 42, and 54m | Watched video (Neutral/smiling babies with cheerful music) | 181 seconds (na) | Mean RSA | 0.02 (0.01) sec |
| Gilissen et al., 2007 | n=78, 36-48m | Watched video (Tik Tak # 15) | 90 seconds (na) | Mean HRV | Alone: 0.66 (12.6); With Parent: 0.50 (11.12);  [units unknown] |
| Gilissen et al., 2008 | n=78, 48m | Watched video (Tik Tak # 15) | 90 seconds (na) | Mean HRV | 0.66 (12.69)  [units unknown] |
| Liew et al., 2011 | n=247, 18 and 30m | Watched video (NA) | NA (na) | Mean RSA | 18m:0 .19 (.11); 30m: 0.30 (.17) [units unknown, multiplied by constant of 10] |
| Morasch et al., 2012 | n=106, 5 and 10m | Watched video (Sesame Street) | 45 seconds (na) | Mean HR and HRV | NA |
| Noten et al., 2019a | n = 61, 45 months | Watched video (calm music and abstract animations) | 60 seconds (na) | Mean HR | Happy: 107.74 (10.86) bmp, Sad: 105.46 (10.48) bpm, Fear: 106.25 (11.17) bmp |
| Notel et al., 2019b | n = 125, 6m | Watched video (relaxing movie) | 120 seconds | RSA | Distress: 3.39 (.38) In(msec2); Frustration: 3.3 (.40) In(msec2) |
| Paret et al., 2015 | n=48, 36-48m | Watched video (NA) | 120 seconds (15 second epochs) | Mean HR and RSA | 103.47 (10.21) bpm, 7.37 (1.08) msec2 |
| Perry et al., 2012 | n=197, 36-48m | Watched video (NA) | 300 seconds (30 second epochs) | Mean RSA | 6.60 (1.12) In(msec2) |
| Wagner et al., 2018a | n=108, 24m | Watched video (NA) | 300 seconds (na) | Mean RSA | 4.86 (.98) In(msec2) |
| Wagner et al., 2018b | n=88, 48m | Watched video (NA) | 120 seconds (120 second epoch) | Mean RSA | 6.20 (1.18) In(msec2) |
| Zeytinoglu et al., 2019 | n = 278, 56 m | Watched video (swimming colourful fish) | 120 seconds (30 second epochs) | Mean RSA | 7.21 (1.11) In(msec2) |
