# Supplementary material for: Physiological measurement of emotion from infancy to preschool: A systematic review and meta‐analysis
Source: Brain Behav. 2020 Dec 17;11(2):e01989. doi: 10.1002/brb3.1989 (PMC7882167; doi:10.1002/brb3.1989)
Supplement: Supplementary file 5 — Table S5 [file BRB3-11-e01989-s005.docx]

| Table 5. Description of Physiological Measurement | | | | | | | | |
| --- | --- | --- | --- | --- | --- | --- | --- | --- |
| **First Author and Year** | **Emotion/ Emotion Task/ Baseline Task** | **Electrode Number/ Placement** | **Num/Duration of Epochs** | **Measurement(s)** | **HR, HP, HRV, or RSA Description** | **Data Loss** | **Data Editing** | **Behavioral Coding** |
| Anderson et al 1999* | **Fear**/ Stranger Wariness/ Before | 3 electrodes/chest | 8/5 seconds | Mean HR for eight episodes | Mean HR in beats per minute | 20-25% loss across tasks at 5 months and 23-25% at 10 months due to incomplete data, equipment failure, or refusal of electrodes | Artifacts handled by abbreviating scoring period to leave a minimum of three artefact free seconds | Verbal and physical fear |
| Baker et al 2012* | **Fear and Guilt**/ Unpredictable Toy and Mishap Guilt Paradigm/ Play | 3 electrodes/back | 7/30 seconds | Mean HR for fear and guilt tasks | Mean HR in beats per minute | 0-13% loss across tasks due to equipment failure or refusal to participate | Data imputation was implemented using linear trend computation | Bodily and facial tension and fear and distress vocalizations |
| Bazhenova et al 2007*^ | **Positive and Neutral**/ Smiling Face and Blank Face/ Play | 3 electrodes/chest | 1-4/30 seconds | Mean HP and RSA for Blank and Smiling episode | ECG signal amplified and HP times to nearest millisecond and sampled every 250 msec; RSA quantified using Porges method with MXedit Software (respiration bandwidth range of 0.24 to 1.04) | 19% loss due to artifacts | Editing consisted of visual detection of outlier points followed by integer division or summation in MXedit Software | Gaze, affect, motor movements |
| Blankson et al 2012^ | **Frustration**/ Locked Box Task and Green Circle Task/ Video | 2 electrodes/chest | NA/NA | Mean RSA and Vagal Withdrawal for frustration task | Quantified using Porges method with MXedit Software (respiration bandwidth range not provided) | % data loss not reported. | Edited and corrected by visual inspection using MXedit Software | Verbal and physical frustration and global regulation |
| Bohlin & Hagekull, 1993* | **Fear/** Stranger Wariness / Before | 3 electrodes/chest | 7/5 seconds | Mean HR for each of the seven episodes | Mean HR in beats per minute | 6% loss due to equipment failure | Artifacts controlled for by excluding all segments showing evidence of body movements and substituting them with the mean of their adjacent values | Verbal and physical fear |
| Brooker et al 2013^ | **Fear**/ Stranger Approach/ Video | NA/NA | NA/ 30 seconds | Mean RSA for each episode | Quantified using Porges method with MXedit Software (respiration bandwidth range of 0.24 to 1.04) | 29% loss, reasons not provided | Edited and corrected by visual inspection using MXedit Software | Facial fear |
| Bush et al 2017^ | **Distress/** Still Face Procedure/ Sitting Quietly | 4 electrodes/right clavicle, lower left rib, right abdomen | 4/30 seconds | Mean RSA for each episode | Quantified using Porges method (respiration bandwidth range of 0.24 to 1.04) | 50% loss due to artifacts and incomplete data | Data deleted if more than 25% of epoch was unscorable; Data cleaning procedures included checking all outliers (.3 SD) by interval and summary scores using MXedit Software | Affect and gaze |
| Buss et al 2004*^ | **Fear**/ Stranger Approach/ Video | NA/NA | NA/30 seconds | Mean HR and RSA for each episode | Quantified using Porges method with MXedit Software (respiration bandwidth range of 0.24 to 1.04) | 25-43% loss across tasks, reasons not provided | Less than 3% of data points edited and majority of 5-min baseline signal and the 2 1⁄2-minute stranger signals preserved; Edited and corrected by visual inspection using MXedit Software | Facial fear |
| Buss et al 2005*^ | **Fear and Distress**/ Stranger Approach and Toy Removal/ Video | 4 electrodes/NA | 1-3/20 seconds | Mean HP and RSA for each episode and/or task | Quantified using Porges method with MXedit Software (respiration bandwidth range of 0.24 to 1.04) | 29-37% loss across tasks due to equipment failure, sensor removal, and artifact | Edited data for 75% of sample; Edited and corrected by visual inspection using MXedit Software | Facial fear, and anger using AFFEX system; crying |
| Busuito et al 2017^ | **Distress**/ Face-to-Face Still-Face/ Sitting Quietly | 2 electrodes/chest | 8/15 seconds | Mean RSA from each episode | Quantified using Porges method with MXedit Software (respiration bandwidth range of 0.24 to 1.04) | 32-36% loss across tasks due to artifacts, child distress, and equipment failure | Outliers replaced by dividing or summing them so they were consistent with adjacent data. Data files requiring editing of more than 2% or had a standard deviation across epochs greater than 1.00 excluded using MXedit Software | Affect and gaze |
| Busuito et al 2019*^ | **Distress**/ Face-to-Face Still-Face/ Sedentary Task | 3 electrodes/chest | 4/30 seconds | Mean HP and RSA for each episode | Quantified using Porges method with MXedit Software (respiration bandwidth range of 0.24 to 1.04) | 4-27% across tasks for HP and 4%-32% for RSA; reasons not provided | Data were edited using the R-peak Editor system, which uses an algorithm to systematically insert missed or correct outlier R-peaks using MXedit Software | Affect and gaze |
| Calkins 1997^ | **Positive and Frustration**/ Puppet Play, Toy Removal in Box/ Video | 3 electrodes/inverted triangle on chest | 8/15 seconds | Mean RSA for each task | Quantified using Porges method with MXedit Software (respiration bandwidth range not provided) | 7-16% loss across tasks due to refusal to participate, other reasons not provided | Data scanned for outlier points relative to adjacent data and replacing those points by dividing them or summing them; Files requiring editing of more than 2% of data were excluded; Typically, less than 1% of HR data required editing using MXedit Software | Duration and latency to smile, fuss, or frown; temperamental reactivity; ER strategies |
| Calkins et al 1992 | **Distress and Fear**/ Arm Restrain, Strange Situation, Stranger Approach/ Sitting Quietly | NA/NA | NA/NA | Mean HP and RSA for each episode and/or task | Quantified using Porges method (respiration bandwidth range not provided) | % data loss not reported | Edited and corrected by visual inspection using MXedit Software | Frequency of crying |
| Calkins et al 2000^ | **Positive, Fear, Frustration, and Distress/** Puppet Play, Spider, Food Denial, and Crying Audio/ Video | 3 electrodes/inverted triangle on chest | 8/15 seconds | Mean HP and RSA for each task | Quantified using Porges method with MXedit Software (respiration bandwidth range of 0.24 to 1.04) | 32% loss due to refusal to wear electrodes, equipment failure, and artifacts | Data scanned for outlier points relative to adjacent data and replacing those points by dividing them or summing them; Files requiring editing of more than 2% of data were excluded using MXedit Software | Verbal and facial affect and behavior |
| Calkins et al. 1998b | **Frustration**/ Plexiglass Barrier, Food Denial, High-Chair, and Toy Removal/ Video | 3 electrodes/inverted triangle on chest | 4/30 seconds | Mean HP and RSA for each task | Quantified using Porges method with MXedit Software (respiration bandwidth range not provided) | 12% loss due to refusal of electrodes, equipment failure, and artifacts | Edited and corrected by visual inspection using MXedit Software | Latency to cry; intensity of distress; frequency of fussing; duration of crying |
| Calkins et al. 2004*^ | **Frustration and Distress** /Crying Audio and Locked Box/ Video | 3 electrodes/inverted triangle on chest | 4/30 seconds (audio) and 8/30 seconds (video) | Mean HP and RSA for each task | Quantified using Porges method with MXedit Software (respiration bandwidth range of 0.24 to 1.04) | 8-11% loss at 2 years and 3-7% at 4 years due to refusal of electrodes, equipment failure, and artifacts | If SD across epochs greater than 1.00 for RSA, episode was excluded; Data scanned for outliers relative to adjacent data and replacing those points by dividing or summing them; Data requiring editing of more than 5% were excluded using MXedit Software | Verbal and physical frustration and global regulation |
| Calkins et al., 1998a^ | **Positive and Frustration**/ Puppet Play and Toy Removal/ Video | 3 electrodes/inverted triangle on chest | NA/NA | Mean RSA for each task | Quantified using Porges method with MXedit Software (respiration bandwidth range not provided) | 4-20% loss across tasks due to artifacts or refusal of electrodes | Edited and corrected by visual inspection using MXedit Software | Duration of and latency to smile or fuss; facial and vocal affect |
| Campos et al 1975* | **Fear**/ Multiple Stranger Approach/ Sitting Quietly | NA/NA | 5/every 3 seconds per phase | Mean HR for each episode | HR sampled every 3 seconds during each phase | 19% loss due to equipment failure or experimental error | NA | Facial affect, gaze, motor activity, and global distress |
| Cho et al 2017^ | **Positive**/ Puppet Play/ Sedentary Task | NA/NA | NA/30 seconds | Mean RSA for task | Quantified using Porges method with Mindware software (respiration bandwidth range of 0.24 to 1.04) | 45% loss due to invalid data | Visual inspection and editing of artifacts in data completed by three scorers who achieved good interrater reliability (agreement = 86%) on 25% of the files using Mindware Software | Duration and latency to freeze, duration of facial fear and bodily fear |
| Dawson et al 2001 | **Positive**/ Peek-A-Boo Play/ Sedentary Task | 2 electrodes/ sternum and left costal region | NA/NA | Mean HR for task | HR samples, collected every 10 millisecond (ms), used to calculate mean HR | % data loss not reported | Output of the R-wave detection was edited and corrected by visual inspection using MXedit Software | NA |
| Eiden et al 2018^#^ | **Distress**/ Arm Restraint/ Video | 3 electrodes/ triangulated on chest; respiration bellows at bottom of the sternum | NA/NA | Mean RSA for task | Quantified using Grossman's method with IBI Analysis software (James Long Company) | 17% loss, reasons not provided | Data files of R-wave intervals were manually edited to remove incorrect detection of R-wave or artifacts by blind assessor | Demandingness, affect, responsiveness to mother, and self-reliance |
| Eisenberg et al. 2012^#^ | **Distress**/ Videos - Crying/ Video | 2 electrodes/armpits at chest level and on back; Respiration bellows placed around abdominal area | NA/NA | Mean RSA for task | ECG data analyzed with IBI analysis software using peak-to-valley method; Baseline RSA was regressed onto Distressed RSA and multiplied by −1 to create measure of RSA suppression that is orthogonal to baseline RSA | 14% loss due to child distress, equipment failure, and artifacts | Edited and corrected by manual visual inspection | NA |
| Feldman et al 2010^ | **Distress**/ Still Face, Face to Face/ Play | NA/NA | NA/NA | Mean RSA for episodes | Quantified using Porges method with MXedit Software (respiration bandwidth range of 0.24 to 1.04) | % data loss not reported | Edited and corrected by visual inspection using MXedit Software | Gaze, affect, vocalizations, and touch |
| Fracasso et al1994*^ | **Positive and Negative**/ Positive and Negative Stimuli/ Sitting Quietly | NA/chest | 40/30 seconds (7 and 10 months) and 60/30 seconds (13 months) | Mean HP and RSA for episodes | Quantified using Porges method with MXedit Software (respiration bandwidth range of 0.24 to 1.04) | 21-40 %, reasons not provided | Edited and corrected by visual inspection using MXedit Software; aberrant values adjusted by integer addition and division of sequential R-intervals; less than 1% of data adjusted because of artifacts | NA |
| Gilissen et al 2007 | **Fear**/ Video - Scary/ Video | 3 electrodes/inverted triangle on chest | 1/60 seconds | Mean HRV for task | RMSSD computed from raw IBI data to index HRV; RMSSD sampled every 10 seconds | 20% loss due to refusal to participate and equipment failure | Unacceptable physiological values (z>3.29) were found and changed into the next most extreme score | Blocking and escape behaviors |
| Gilissen et al 2008 | **Fear**/ Video - Scary/ Video | 3 electrodes/inverted triangle on chest | 1/60 seconds | Mean HRV for task | RMSSD measured as index of HRV | 11% loss due to equipment failures and outliers | Missing and unacceptable values replaced with mean HRV values for subgroup matched for child gender and age. | NA |
| Gray et al 2017^ | **Distress**/ Still Face Paradigm/ Play | NA/NA | NA/NA | Mean RSA for episodes | IBIs detrended using moving polynomial filter to remove slow trends; RSA estimated from power spectral analysis using discrete Fourier transform and quantified in range for 4-month-old infants (0.30 - 0.75 Hz); RSA values were winsorized to 3 standard deviations from mean and natural log transformed | 17% loss due to artifacts, equipment failure, human error, or incomplete data | NA | NA |
| Haley et al 2003* | **Distress**/ Still Face Paradigm/ Play | 2 electrodes/chest | 1/120 seconds | Mean HR for episodes | NA | 12% loss due to distress or data loss | Data files digitally filtered and reviewed for artifacts; filtered data replaced with mean values of the surrounding intervals; no more than 2-3% of any file required filtering | Gaze, affect, fussing, and crying |
| Ham et al 2006 | **Distress**/ Still Face Paradigm/ Play | NA/NA | 1/120 seconds | Mean HR and RSA for episodes | Pilot study; details not provided | 25% due to equipment failure | NA | Used Infant Caregiver Engagement Phases, codes affect, gaze, and vocalizations |
| Ham et al 2009*^ | **Distress**/ Still Face Paradigm/ Play | 3 electrodes/shoulders and side | 1/120 seconds | Mean HR and RSA for episodes | Quantified using algorithm with filter for infant respiration (respiration bandwidth range of 0.24 to 1.04) in Chart software | 6 - 28% loss across tasks due to equipment failure or noncompliance | NA | Used Infant Caregiver Engagement Phases, codes affect, gaze, and vocalizations |
| Hay et al 2017* | **Distress**/ Teddy Bear Picnic/ Unknown Task | NA/ upper left leg | NA/15 seconds | Mean HR for episodes | HR sampled at 30 HZ and represented as bpm | 7% loss due to incorrect procedure, wrong sensors, refusal to wear electrodes, and equipment failure | Logarithmic transformation applied to HR data to improve normality of distribution | Used Distress Observation System (DOS) to code vocal distress |
| Hill-Soderlund et al 2008^ | **Fear**/ Ainsworth Strange Situation / Play | 2 electrodes/chest | 4/15-30 seconds | Mean RSA for episodes | Quantified using Porges method (respiration bandwidth range of 0.24 to 1.04) | 46-62%% loss across tasks due to equipment failure and artifacts; Excluded only if they were completely missing all physiological data. | Data files that required editing of more than 10% of the data were not included in the analyses and were considered missing. | NA |
| Holochwost et al 2014^ | **Distress**/ Still Face Paradigm/ Sitting Quietly | 2 electrodes/chest | 8/15 seconds | Mean RSA for episodes | Quantified using Porges method with MXedit software (respiration bandwidth range of 0.24 to 1.04) | 12-23% loss across tasks, reasons not provided | Edited for artifacts using MXEdit software; Data files requiring editing of more than 10% were excluded | NA |
| Johnson et al 2014^ | **Distress**/ Arm Restraint, Still Face Paradigm/ Sitting Quietly | 3 electrodes/forehead and chest | 8/15 seconds | Mean RSA for episodes | Computed using Fast Fourier transformation with Mindware software (respiration bandwidth range of 0.24 to 0.40) | 24% loss due to artifacts, electrode placement difficulties, and equipment failure | Correction of artifact, motion, and error in automated marking of R waves was performed manually; | Facial affect coded using Mangold INTERACT software |
| Liew et al 2011^#^ | **Distress**/ Videos - Crying/ Video | 3 electrodes /lower ribs and back; respiration cord around abdominal area | 1/42 seconds | Mean RSA for task | ‘Peak-to-valley’ metric using James Long software; RSA data multiplied by constant value of 10 for analyses. RSA suppression indexed by reversed score of standardized residualized RSA change score (calculated by computing regression with RSA during neutral film as predictor and RSA during emotion film as outcome and multiplying value by −1) | 13% loss at 18 months and 10% at 30 months, reasons not provided | For baseline, scores three SDs above/below mean coded as missing so values were estimated in SEM or multiple imputations procedures. | NA |
| Mireault et al 2018* | **Positive**/ Absurd Event/ Play | 3 electrodes/inverted triangle on chest | 1/45 seconds | Mean HR for episodes | HR in beats per minute | 50% loss across sample, reasons not provided; included only children with complete data | NA | Duration of smiling/laughing and gaze |
| Moore 2009 | **Positive, Angry, Neutral, and Distress**/ Emotion Evoking Task and Still Face paradigm/ Sitting Quietly | NA/chest | 8/15 seconds | Mean RSA for episodes and/or task | Quantified using Porges method with MXedit software (respiration bandwidth range not provided) | 44% loss due to distress, equipment failure, and artifacts | Data scanned for outliers; editing of more than 2% or RSA standard deviation across epochs greater than 1.00 were excluded using MXedit Software | Affect and gaze |
| Moore et al 2004*^ | **Distress**/ Still Face Paradigm/ Sitting Quietly | 3 electrodes/inverted triangle on chest | 8/15 seconds | Mean HR and RSA for episodes | Quantified using Porges method with MXedit software (respiration bandwidth range of 0.24 to 1.04) | 14-17% loss due to equipment failure and movement artefacts | Data scanned for outliers relative to adjacent data and replacing those points by dividing them or summing them; Data files requiring editing of more than 2% of the data were excluded using MXedit software | Affect and gaze |
| Moore et al 2009^ | **Distress**/ Still Face Paradigm/ Sitting Quietly | NA/chest | 8/15 seconds | Mean HP and RSA for episodes | Quantified using Porges method with MXedit software (respiration bandwidth range not provided) | 37-45% loss across tasks due to equipment failure, artifacts, distress, falling asleep | Data scanned for outliers using MXedit Software | Affect and gaze |
| Morasch et al 2012 | **Distress**/ Arm Restraint/ Video | 3 electrodes/right collarbone, lower left rib, ground at scalp | NA/NA | Mean HR and HRV for task | Mean heart rate in beats per minute and heart rate variability | 19% loss at 5 months and 9% at 10 months due to artifacts, equipment failure, and electrode misplacement | Artifact scored by trained research assistant for movements using IBI Analysis software developed by James Long Company | Gaze and affect |
| Noten et al., 2019a* | **Positive, Sadness, and Fear**/ Video clips/ Video clip | 3 electrodes/right collarbone, left apex of heart, and right side between lower two ribs | 1/50 seconds | HR | Mean heart rate in beats per minute | 11% loss due to refusal to wear electrodes or loose electrodes | Data were visually checked by trained researcher and adjusted manually | NA |
| Noten et al., 2019b^ | **Distress and Frustration**/ Still-Face Paradigm and Car Seat Task/ Video | 3 electrodes/right collarbone, left apex of heart, and right side between lower two ribs | 1/120 seconds for Still-Face and 1/60 seconds for Car Seat | RSA | Quantified using Grossman's method with IBI Analysis software (VU-DAMS software) | 8% loss for still-face paradigm and 15% loss for car seat task due to artefacts, technical problems, and child refusal | Data were visually checked by trained researcher and adjusted manually | Vocalizations, self-soothing, and escape behaviours |
| Paret et al 2015*^ | **Fear**/ Interesting but Scary/ Video | 3 electrodes/inverted triangle on chest | 8/15 seconds | Mean HR and RSA for tasks | RMSSD is sensitive to fluctuations in IBI in respiratory range, representing a high-pass filter that captured high frequency RSA; its natural logarithm (lnRMSSD) used in analyses | 45% loss due to equipment failure, artifacts, and child refusal | Waveforms examined for artifacts that interfere with accurate extraction of IBI length; edited by replacement with non-voltage peak values; Epochs requiring more than 10% editing or with standard deviation greater than 1.00 were excluded | Nonverbal anxiety and verbal responsiveness |
| Perry et al 2012^ | **Frustration**/ Frustrating Puzzle Task/ Video | 2 electrodes/chest and stomach | 12/15 seconds | Mean RSA for task | Quantified using Porges method with MXedit software (respiration bandwidth range of 0.24 to 1.04) | 19% loss due to refusal to wear electrodes, artifacts | Editing consisted of examining outliers and dividing or summing; Data files requiring editing of more than 10% of the data were excluded using MXedit software | Verbal and physical distractions |
| Perry et al 2016^ | **Distress**/ Arm Restraint/ Sedentary Task | 2 electrodes/right collarbone and lower left rib | NA/NA | Mean RSA for task | Spectral analysis used to calculate RSA using discrete Fourier transform with frequency band for quantification of 0.24–1.04 Hz. The RSA data were transformed using natural log to normalize the distribution. | 47% loss due to artifacts, equipment failure, and incomplete data | ECG signal visually inspected for software-detected R-waves; Movement artifact was designated by absence of at least three consecutive R-waves. These epochs were excluded | Mother-orientation and distraction |
| Pratt et al. 2015^ | **Distress**/ Still Face with Touch or Arm Restraint/ Play | 3 electrodes/NA | 8-12/15 seconds | Mean RSA for episode | Quantified using Porges method with MXedit software (respiration bandwidth range of 0.24 to 1.04) | 16% due to equipment failure, and child discontinuation | Data scanned for outliers using MXedit Software | Gaze, affect, vocalizations, touch, autonomic response, motor response, and self-soothing behavior |
| Provenzi et al 2015 | **Distress**/ Face-to-Face Still-Face/ Play | 3 electrodes/inverted triangle on chest | 12/10 seconds | Mean RSA for episode | Quantified using Porges method with MXedit software (respiration bandwidth range of 0.24 to 1.04) | 41% due to equipment failure, missing data, and artifacts | IBI data screened offline to correct potential errors in automatic detection of R-wake peaks | Negative, positive, and object/ environment engagement, and social monitoring |
| Provost et al 1979* | **Fear and Frustration**/ Stranger Situation with Locked Box/ Before | 2 electrodes/left nipple and left shoulder blade in lining of jacket | 36/5 seconds | Mean HR for episode and/or task | Mean heart rate in beats per minute | 35% loss due to child distress and unusable recordings | NA | Motor, hedonic tone, affect, and vocalizations |
| Qu et al 2018^ | **Distress**/ Face-to-Face Still-Face / Play | 3 electrodes/ collarbone and lower ribs | 8/15 s epochs | Mean RSA for episode | Quantified using Porges method (respiration bandwidth range not provided) | 20% loss due to artifacts | NA | Affect coded using INTERACT |
| Rash et al 2015^#^ | **Distress and Frustration**/ Toy Retraction, Plexiglass Barrier, Arm Restraint/ Sitting Quietly | 2 electrodes/clavicle and ribcage | 3/45 seconds | Mean RSA for task | Quantified as average power spectral density of R-R fluctuations occurring in respiratory band (0.24—1.04 Hz) using Grossman method | 28% loss due to acquisition error, unusable data, incomplete recordings, artifacts, and infant fussing | Outliers from previous and subsequent 50 IBIs by a value greater than 20% were interpolated; No participant in final sample required more than 5% data interpolation | NA |
| Rash et al 2016^#^ | **Distress and Frustration**/ Toy Retraction, Plexiglass Barrier, Arm Restraint/ Sitting Quietly | 2 electrodes/ clavicle and ribcage | 3/45 seconds | Mean RSA for task | Quantified using power spectral density of R-R fluctuations during respiration (bandwidth range of 0.24 to 1.04) | 17% loss due to equipment failure, artifacts, missing data, and outliers | Outliers identified as values exceeding z-score of 3.29 and adjusted according to recommendations; No more than four values (1.5%) were adjusted for any variable | NA |
| Santesso et al 2007* | **Positive and Fear**/ Audio - ID speech/ Unknown Task | 2 electrodes/ chest | 1/60 seconds | Mean HP for task | HR bandpass filtered between 1 - 100 Hz and sampled at 512 Hz | % loss and reasons not provided | All data were visually inspected for artifact identification and edited by a second team of coders. | NA |
| Schmidt et al 2003* | **Positive, Fear, and Sadness**/ Musical Pieces/ Unknown Task | 2 electrodes/ chest | 1/30 seconds | Mean HP for task | HR bandpass filtered between 1 - 100 Hz and sampled at 512 Hz | 2% at 9 months and 38% at 12 months due to equipment failure | All data were visually inspected for artifact identification and edited by a second team of coders. | NA |
| Scrimgeour et al 2016 | **Disappointment**/ Disappointment Task/ Sedentary Task | 3 electrodes/ torso | 1/30 Seconds | Mean RSA for task | RSA defined as natural log integral of .24 to 1.04 Hz power band using Mindware HRV | 56% had incomplete data (but were imputed in SPSS); reasons not provided | All data were visually inspected for artifact identification and edited by a second team of coders. | NA |
| Skarin 1977* | **Fear**/ Stranger Approach/ Before | 3 electrodes/ breasts and ankle | NA/0.5 seconds | Mean HR for task | Each 5 seconds segment of HR was subtracted from baseline HR in each segment of approach | 3% loss due to equipment failure | NA | Facial expressions |
| Spangler et al 1993* | **Fear/** Ainsworth Strange Situation/ Play | 3 electrodes/inverted triangle on chest | NA/NA | Mean HR for episodes | HP converted to HR in beats per minute for each 1-sec interval | 26% loss due to non-valid HR scores | HP were controlled for by artifacts by computer using Foerster (1984) criteria | Facial emotion and orientation to mother or objects |
| Stone et al 2013 | **Distress**/ Arm Restraint and Still Face-Face to Face/ Unknown Task | NA/NA | NA/NA | Mean HP and RSA for episodes and/or task | Quantified using Porges method with MXedit software (respiration bandwidth range not provided) | 23% loss due to incomplete data | MXedit software used to edit outliers due to movement and recording artifacts | NA |
| Vaughn et al 1979* | **Positive and Fear**/ Peek a Boo then Mask/ Sitting Quietly | NA/sternum | NA/NA | Mean HR and time to crying for task | Nothing reported on HR | Only included infants with complete records, original sample not reported | NA | First cried and facial expression changed to cry face |
| Wagner et al 2018a^ | **Fear**/ Stranger Challenge/ Video | 3 electrodes/chest | NA/NA | Mean RSA for task | MxEdit computed RSA in 0.24–1.04 Hz frequency bandwidth | 4% loss for baseline and 10% for stranger procedure due to unusable cardiac data | Recording errors in IBIs edited using MxEdit software | NA |
| Wagner et al 2018b^ | **Anger**/ Narrated Comic Strip/ Video | 3 electrodes/chest | 1/60 seconds | Mean RSA for task | MxEdit computed RSA in 0.24–1.04 Hz frequency bandwidth | 5% loss, reasons not provided | Recording errors in IBIs edited using MxEdit software | NA |
| Waters et al 1975 | **Fear**/ Stranger Wariness/ Before | NA/NA | NA/NA | Mean HR and time to peak HR | NA | % data loss not reported; removed participants who smiled or cried from analyses | NA | Positive, neutral, and negative responses |
| Weinberg et al 1996*^ | **Distress/** Face to Face Still Face/ Play | NA/NA | 12/10 seconds | Mean HR and RSA for episodes | Analyzed with MXEdit software (respiration bandwidth range not reported | % data loss not reported | Recording errors in IBIs edited using MxEdit software | Affect coded using AFFEX system and behavior coded using Infant Regulatory Scoring System |
| Zeegers et al 2017^#^ | **Fear**/ Stranger Approach/ Unknown Task | 2 electrodes/collarbone and rib | 1/30 seconds | Mean HRV for task | RMSSD of successive normal-to-normal intervals | 17% loss at 4 months and 27% at 12 months for Baseline; 28% loss at 4 months and 38% at 12 months for tasks due to families not visiting lab, artifacts, and equipment failure | R-waves were identified and adjusted for artifacts. | NA |
| Zeytinoglu et al., 2019^ | **Frustration**/ Locked Box Task and Toy Removal/ Videos | 2 electrodes/collarbone, lower left rib, and lower right rib | 8/30 seconds for lock box and 4/30 seconds for toy removal | Mean RSA | Computed using Fast Fourier transformation with Mindware software (respiration bandwidth range of 0.24 to 1.04) | 11% loss in locked box and 13% in toy removal due to artefacts, equipment failure, and electrode placement errors | All data were visually inspected for artifact identification and edited by trained researchers. | Overall composite of global regulation, verbal negativity, and latency to distress |

Notes: * Included in HR meta-analysis; ^ Included in RSA meta-analysis; # Included in HRV meta-analysis.
