# Supplementary material for: Physiological measurement of emotion from infancy to preschool: A systematic review and meta‐analysis
Source: Brain Behav. 2020 Dec 17;11(2):e01989. doi: 10.1002/brb3.1989 (PMC7882167; doi:10.1002/brb3.1989)
Supplement: Supplementary file 6 — Table S6 [file BRB3-11-e01989-s006.docx]

| Table 6. Description of Physiological Results | | | | | | |
| --- | --- | --- | --- | --- | --- | --- |
| **Measure** | **Article** | **Sample Size/Age** | **Baseline Mean (SD)** | **Task Mean (SD) and Effect Size (Cohens d)** | **Significant Differences**  **(Condition, Age, and Sex)** | **Significant Relationship(s) with Behavior** |
| **Heart Rate (HR)** | | | | | | |
| HR | Anderson et al 1999* | **45**/ 5 and 10 months | NA | 5 months: -4.1 bpm; 10 months +1.5 bpm from baseline; all ds=NA | Condition x Age Effect (↓ HR at 5 months and ↑ HR at 10 months from baseline); Did not test for Sex Effect | ↑ wariness = ↑ HR |
| HR | Baker et al. 2012* | **70, 64, 61**/ 12, 24, and 36 months | Year 1: 136.43 (12.15) bpm; Year 2: 121.86(9.74) bpm; Year 3: 113.72 (9.84) bpm | Year 1: 141.34 (15.78) bpm, d=.35; Year 2: 133.11 (13.97) bpm, d=.94; Year 3: 117.72 (14.77) bpm, d=.32 | Age (HR ↓ with age) and Condition Effect; No Sex Effect | None reported |
| HR | Bazhenova et al 2007*^ | **16/** 3-4 months | 155 (7.75) bpm | 148 (4.69) bpm, d=1.13 | Condition Effect; Did not test for Sex Effect | None reported |
| HR | Bohlin & Hagekull, 1993* | **31**/ 10-13 months | Mpres: 130.2 bpm; Mabs: 146.8 bpm | Distance Phases Mpres = 130.3 bpm, d=NA and Mabs = 147.7 bpm; d=.98; Intrusion Phases were Mpres = 136.4 bpm, d=NA and Mabs = 150.9 bpm, d=1.02 | Condition Effect (Intrusion vs Baseline); Did not test for Sex Effect | ↑ wariness = ↑ HR |
| HR | Buss et al 2004*^ | **46**/ 24 months | 119.03 (8.53) bpm | 122.98 (9.27) bpm, d=.45 | Did not test for Condition or Sex Effect | ↑ Freeze composite = ↓HR |
| HR | Buss et al 2005*^ | **68/ 24 months** | 119.03 (8.53) bpm | Stranger: 122.75 (9.27) bpm, d=.42; Toy Removal: 126.98 (14.46) bpm, d=.68 | Condition Effect (Toy Removal vs. Stranger and Baseline; Stranger vs Baseline;) No Sex Effect | ↑ negative affect = ↑ HR in Stranger Approach and Toy Removal |
| HR | Busuito et al 2019*^ | **116/** 6 months | 138 (9.79) bpm | Play: 137 (10.72) bpm, d=.10; SF: 145 (12.49) bpm, d=.63; Reunion: 144 (13.14) bpm, d=.52 | Condition Effect (SF vs Play); No Sex Effect | ↑ positive and negative affect = ↓HR in play and reunion; ↑ negative affect = ↓ HR in SF |
| HR | Calkins et al 1992 | **48-52**/ 5, 14, and 24 months | NA | NA; d=NA | Did not test for Condition or Sex Effect | None reported |
| HR | Calkins et al 2004*^ | **135 and 115 /** 2 and 4.5 years | Age 2: 109 (10.89) bpm; Age 4.5: 97 (12.86) bpm | Age 2-Empathy: 113 (10.16) bpm, d=.38; Frustration: 116 (10.91) bpm, d=.64; Age 4.5-Empathy: 98 (12.11) bpm, d=.08; Frustration: 101 (11.24) bpm, d=.33 | Did not compare HR by Condition; Did not test for Sex Effect | None reported |
| HR | Calkins et al 1998b | **73**/ 18 months | NA | 147 bpm; d=NA | Did not test for Condition Effect; No Effect of Sex | None reported |
| HR | Campos et al 1975* | **80**/ 5 and 9 months | NA | NA; all ds=NA | Age Effect (9 months ↑ HR vs. 5 months ↓HR during Task); No Sex Effect | ↑ negative affect = ↑ HR |
| HR | Dawson et al 2001 | **61/**13-15 months | 128 bpm | Mother Play: 131 bpm, d=.44; Experimenter play: 126 bpm, d=NA | Condition Effect (Both tasks vs Baseline); Did not test for Sex Effect | NA |
| HR | Fracasso et al 1994*^ | **44-58**/ 5, 7, 10, and 13 months | 5 months: 142 (9.76) bpm; 7 months: 138 (9.90) bpm; 10 months: 135 (10.30) bpm; 13 months: 136 (10.80) bpm | 7 months: 144 (10.05) bpm, d=.61; 10 months: 137 (9.89) bpm, d=.20; 13 months: 139 (10.92) bpm, d=.28 | Age and Condition effects; Did not test for Sex Effect | NA |
| HR | Haley et al 2003* | **43**/ 3-4 months | Play 1: 146.60(11.92) bpm | SF1: 148.79 (11.60) bpm, d=.19; Reunion 1: 146.55 (12.07) bpm, d=.004; SF2: 151.15 (12.21) bpm, d=.38; Reunion 2: 149.11(13.15) bpm, d=.20 | Condition Effect (SF1 versus Play1 and Reunion; SF2 versus Reunion 1); Sex Effect (Boys had ↓ HR than girls) | ↑ negative affect = ↑ HR in SF II |
| HR | Ham et al 2006 | **12/ 5 months** | FF: Recovered: 145 bpm Stable: 142 bpm; Dysregulated: 137 bpm; Protest: 152 bpm | SF-Recovered:145 bpm; Stable: 145 bpm; Dysregulated: 155 bpm; Protest: 153 bpm; Reunion-Recovered: 150 bpm; Stable: 145 bpm; Dysregulated: 170 bpm; Protest: 160 bpm; all ds=NA | Descriptive; Did not test for Condition or Sex effect | ↑ protest = ↑ HR in all SF episodes |
| HR | Ham et al. 2009*^ | **18/ 5 months** | 143.57 (9.97) bpm | 151.67 (11.93) bpm, d=.76 | Condition Effect (SF versus FF;) Did not test for Sex Effect | ↑ mother engagement = ↓ HR; ↑ negative affect = ↑ HR in SF and Reunion; ↑ social engagement = ↑ HR overall |
| HR | Hay et al 2017* | **255**/ 12 months | 132.81 (21.04) bpm | 141.16 (15.21) bpm, d=.46 | Sex Effect (Boys had ↓ HR during task) | ↑ vocal distress = ↑ HR |
| HR | Mireault et al 2018* | **34, 17**/4, 6 months | 139.4 (12.43) bpm | 4 months: Absurd-Neutral: 136.1 (10.85) bpm, d=.29; Absurd-Cued: 138.9 (11.62) bpm, d=.04; 4 Months: HR Absurd Neutral 136.5 (10.65) bpm, d=.25; 6 months: 130.7 (11.07) bpm, d=.74 | Condition Effect (Absurd Cued vs Play, Absurd Ordinary at 4 and 6 months), Age Effect (4 mos had ↑ HR than 6 mos for Absurd Neutral); Did not test for Sex Effect | ↓ gaze to parent = ↑ HR at 4 months; ↑ gaze to parent = ↑ HR at 6 months |
| HR | Moore et al 2004*^ | **60**/ 3 months | 146 (10.89) bpm | Play: 147(10.33) bpm, d=.010; SF: 152 (12.19) bpm, d=.52; Reunion: 148 (11.90) bpm, d=.18 | Condition Effect (Baseline vs SF and Reunion; SF vs Play and Reunion; Reunion vs Play; Change SF vs Play and Reunion; Reunion vs Play) | ↑ negative affect = ↑ HR during in Reunion; ↑ negative affect = ↑ change HR in SF and Reunion |
| HR | Morasch et al 2012 | **75 and 84/ 5 and 10 months** | NA | 5 months: NA; 10 months: NA; d=NA | Condition Effect (for HR, not HRV at 5 mos and both at 10 mos); No Effect of Sex; Did not test for Age Effect | ↑ negative affect = ↑ HR in post-distress phase at 5 and 10 months; ↑ negative affect = ↑ HRV and ↓ gaze duration = ↑ HR in post-distress phase at 10 months |
| HR | Noten et al., 2019a* | **54/ 45 months** | Happy: 107.62 (11.08) bpm; Sad: 105.49 (10.48) bpm; Fear: 106.25 (11.17) bpm | Happy: 105.35 (10.87) bpm, d=.21; Sad: 101.49 (11.31) bpm, d=.36; Fear: 103.22 (11.47) bpm, d=.27 | Condition Effect (Each video vs baseline); No Effect of Sex | NA |
| HR | Paret et al. 2015*^ | **48**/ 44 months | 103.47 (10.21) bpm | 111.55 (11.00) bpm, d=.77 | Condition Effect (↑ HR for IbS ); No Effect of Sex | None reported |
| HR | Provost et al 1979* | **26/ 9-12 months** | With Mother: 147.2 bpm; Frustration Baseline: 148.5 bpm; Isolation Baseline: 147.7 bpm; Reunion Baseline: 156.4 bpm | Play with Mother: 144.2 bpm, d=.28; Frustration: 156.5 bpm, d=.87; Isolation: 162.5 bpm, d=.70; Reunion: 148.0 bpm, d=.38 | Condition Effect (↑ HR in anger and distress tasks); Did not test for Sex Effect | None reported |
| HR | Santesso et al 2007* | **34/** 9 months | 138 bpm | Comfort: 134 bpm, d=.97; Surprise: 135 bpm, d=.88; Fear: 134 bpm, d=.69 | Condition Effect (↓ HR in task); Did not test for Sex Effect | NA |
| HR | Schmidt et al 2003* | **33-52/** 3, 6, 9, and 12 months | 3 months: 147 (15.53) bpm; 6 months: 144 (11.74) bpm; 9 months: 136 (11.42) bpm; 12 months: 131 (11.56) bpm | 3 months: 142(14.13) bpm, d=.34; 6 months: 139 (10.48) bpm, d=.46; 9 months: 139 (11.54) bpm, d=.26; 12 months: 130 (11.74) bpm, d=.09 | Condition Effect (Music vs Baseline at 3, 6, and 9 months); Did not test for Age or Sex Effect | NA |
| HR | Skarin 1977* | **32**/ 5-7, 10-12 months | NA | NA; d=.83 | Condition Effect (HR↑ with task); Age Effect (HR↑ with age); No Effect of Sex | None reported |
| HR | Spangler et al 1993* | **30**/ 12 months | Insecure: 142.3 (18.2) bmp; Disorganized: 134.2 (6.9) bpm; Secure: 140.7(12.0) bpm | Infant Alone 1 Insecure: 148.5 (17) bpm, d=.36; Disorganized: 139.8 (22) bpm, d=.35; Secure: 149.6 (11.8) bpm, d=.76; Infant and Stanger 2 Insecure: 139.9(17.6) bpm, d=.14; Disorganized: 150 (22.8) bpm, d=.95; Secure: 147.2 (19.6) bpm, d=.41 | Condition Effect (Infant Alone vs Baseline); Did not test for Sex Effect | None reported |
| HR | Stone et al 2013 | **78**/ 6 months | Bradycardia: 154 (12.08) bpm; Non-Bradycardia: 151 (11.88) bpm | Bradycardia: 153 (11.94) bpm, d=.08; Non-Bradycardia: 152 (20.97) bpm, d=.06 | Did not test for Condition Effect; No Effect of Sex | NA |
| HR | Vaughn et al 1979* | **16**/ 8-16 months | 130 bpm | NA, d=1.08 | Condition (↑ HR prior to crying onset) | Each child showed a ‘cry face’ prior to crying |
| HR | Waters et al. 1975 | **26**/ 5 and 7 months | 120 bpm | Wary vs Non-Wary Peak: 5 months: + 4 bpm for both; 7 months: + 12 bpm vs +4 bpm; 9 months: +10 bmp vs + 4 bpm; Peak HR Pick-Up 5 months: +16 bmp vs + 6 bpm; 7 months: +10 bpm vs +6 bpm; 9 months: +14 bpm vs +4 bpm; all ds=NA | Did not test for Condition, Age, or Sex Effect | ↑ gaze aversion just before ↑ peak HR acceleration |
| HR | Weinberg et al 1996*^ | **45**/ 6 months | 138.20 bpm | SF: 143.88 bpm; Reunion: 139.91 bpm; d=.73 | Condition Effect (↓HR in SF); Did not test for Sex Effect | None reported |
| **Heart Rate Variability (HRV)** | | | | | | |
| HRV^$^ | Eiden et al 2018^#^ | **57/ 9 months** | Boys: 0.016 (.01) sec; Girls: 0.02 (.01) sec | Boys change: -.003 (.006) sec, d=.35; Girls change: -.004 (.01) sec, d=.40 | Did not test for Condition Effect; No Effect of Sex | ↑ regulation = ↓ RSA suppression |
| HRV^$^ | Eisenberg et al. 2012^#^ | **202/ 18 months** | 0.02 (0.01) sec | Distress: 0.03 (.02) sec, d=.5; Suppression: 0.00 (.01), d=.00 | No Effects of Age or Sex | NA |
| HRV^$^ | Gilissen et al 2007 | **78/ 44 months** | Alone: 0.66 (12.6); With Parent: 0.50 (11.12); [units unknown] | Fearful alone: -5.06 (11.55), d=.63; Fearful with parent: -4.21 (13.63), d=.30 [units unknown] | Condition Effect (no effect of Parent presence); Did not test for Sex Effect | None reported |
| HRV^$^ | Gilissen et al 2008 | **78/ 44 months** | 0.66 (12.69) [units unknown] | Fear: -5.06 (11.55), d=.63 [units unknown] | Condition Effect; Did not test for Sex Effect | NA |
| HRV^$^ | Liew et al 2011^#^ | **247/ 18 and 30 months** | Residuals 18 months: .19 (.11); 30 months: .30 (.17) [units unknown] | Suppression 18 months: .00 (.07), d=.00; 30 months: .00 (.07), d=.00 [units unknown] | Did not test for Condition Effect; No Effect of Sex; Age Effect (Baseline 18 months vs 30 months) | NA |
| HRV^$^ | Rash et al 2015^#^ | **194/ 6 months** | 461.93 (425.52) ms2/Hz | 396.92 (320.24) ms2/Hz, d=.16 | Condition Effect (↓ RSA in task); No Effect of Sex | NA |
| HRV^$^ | Rash et al 2016^#^ | **254/ 6 months** | 495.31 (402.72) ms2/Hz | Change = -55.14 (387.70), d=.14 | Condition Effect (↓ RSA in task); No Effect of Sex | NA |
| HRV^$^ | Zeegers et al 2017^#^ | **84, 97/ 4, 12 months** | 4 months: 16.57 (9.85) msec; 12 months: 29.88 (16.75) msec | Decline 4 months: −1.16 (10.01) msec, d=.12; 12 months: −1.92 (15.30) msec, d=.12 | Did not test for Condition, Age or Sex Effect | NA |
| **Respiratory Sinus Arrhythmia (RSA)** | | | | | | |
| RSA | Bazhenova et al 2007*^ | **16/ 3-4 months** | 3.2 (0.3) In(msec2) | 2.8 (0.3) In(msec2), d=1.38 | Condition Effect | None reported |
| RSA | Blankson et al 2012^ | **263/ 42 months** | 6.41 (1.32) In(msec2) | Locked Box RSA withdrawal 1.25 (.84) In(msec2), d=1.09; Green circle RSA withdrawal 0.67 (.69) In(msec2), d=.64 | Did not test for Condition or Sex Effect | None reported |
| RSA | Brooker et al 2013^ | **88/ 6 months** | 3.55 (.77) In(msec2) | Suppression = .01 (.96) In(msec2), d=.01 | Did not test for Condition or Sex Effect | ↑ stranger fear = ↓ RSA at baseline |
| RSA | Bush et al 2017^ | **135/ 6 months** | Play 1 = 4.27 (1.04) In(msec2) | SF1 = 3.99(1.21) In(msec2), d=.18; Reunion 1 = 4.21 (1.21) In(msec2), d=.05; SF2 = 3.73 (1.16) In(msec2), d=.49; Reunion 2 = 4.10 (1.15) In(msec2), d=.16; Last SF (1 or 2) = 3.69 (1.27) In(msec2), d=.50 | Condition Effect (SF 1 and 2 vs Play); Did not test for Sex Effect | None reported |
| RSA | Buss et al 2004*^ | **46**/ 24 months | 5.06 (.95) In(msec2) | 4.71 (1.05) In(msec2), d=.35 | Did not test for Condition or Sex Effect | None reported |
| RSA | Buss et al 2005*^ | **46/ 24 months** | 5.06 (.95) In(msec2) | Stranger: 4.71 (1.05) In(msec2), d=.35; Toy Removal: 4.48 (1.37) In(msec2), d=.50 | Condition Effect (Stranger and Toy Removal vs Baseline); No Sex Effect | None reported |
| RSA | Busuito et al 2017^ | **50/ 6 months** | 3.65 (1.07) In(msec2) | SF = 3.40 (.93) In(msec2), d=.25; Reunion = 3.61 (1.09) In(msec2), d=.04 | Condition Effect (Play vs SF); Sex Effect (Boys ↑ RSA at Baseline) | None reported |
| RSA | Busuito et al 2019*^ | **109/ 6 months** | 3.18 (0.85) In(msec2) | SF: 3.85, (1.13) In(msec2), d=.59; Reunion: 3.79 (1.19) In(msec2), d=.59 | No Effect of Task (by episode); Sex Effect (Boys ↑ RSA at Baseline) | ↑ positive affect = ↑ RSA in play |
| RSA | Calkins 1997^ | **37/ 24-36 months** | 5.66 In(msec2) | RSA Positive: 5.4 In(msec2), d=.48; Negative: 5.27 In(msec2), d=.7; RSA difference Positive: 0.30 (range 2.56 to 2.42), Negative: 0.33 (range 1.25 to 1.97); | Condition Effect (all tasks vs Baseline); No Effect of Age or Sex | ↑ positive and negative reactivity = ↑ RSA in baseline |
| RSA | Calkins et al 1992 | **48-52**/ 5, 14, and 24 months | NA | NA; d=NA | Did not test for Condition or Sex Effect | None reported |
| RSA | Calkins et al 2000^ | **85/ 24 months** | 5.75 (11.41) In(msec2) | Puppets: 5.17 (1.24) In(msec2), d=.07; Spider: 4.94 (1.15) In(msec2), d=.10; Audio: 5.25 (1.28) In(msec2), d=.06; Food Denial: 5.00 (1.25) In(msec2), d=.09 | Condition Effect (all tasks vs Baseline); Sex Effect for Baseline (Boys ↑ RSA than girls) | None reported |
| RSA | Calkins et al 2004*^ | **135 and 115/ 24 and 53 months** | Age 2: 5.76 (1.4) In(msec2); Age 4.5: 5.95 (1.35) In(msec2); | Age 2: Empathy: 5.26 (1.25) In(msec2), d=.38; Frustration: 5.02 (1.24) In(msec2), d=.56; Age 4.5: Empathy: 6.00 (1.39) In(msec2), d=.04; Frustration: 5.65 (1.34) In(msec2), d=.22 | Condition Effect (Baseline vs Empathy and Frustration at Age 2 and vs Frustration at age 4.5); Age Effect; Did not test for Sex Effect | None reported |
| RSA | Calkins et al 1998b | **73/ 18 months** | NA | 4.05 In(msec2), d=NA | Did not test for Condition Effect; No Effect of Sex | ↑ distress = ↑ RSA in barrier task |
| RSA | Calkins et al., 1998a^ | **52/ 18 months** | 5.36 (1.22) In(msec2) | Positive: 4.98 (1.19) In(msec2), d=.32; Negative: 4.60 (1.13) In(msec2), d=.65; Suppression-positive: .33 (.77), d=NA; negative = .72 (.71), d=NA | Did not test for Condition Effect; no Effect of Sex | ↑ distraction and ↓ aggression = ↑ RSA suppression in Barrier task |
| RSA | Cho et al 2017^ | **62/ 24 months** | 4.46(1.07) In(msec2) | Change: .00 (.99), d=.00 | Did not test for Condition Effect; No Effect of Sex | ↓ fear = ↑ RSA suppression |
| RSA | Feldman et al 2010^ | **53/ 6 months** | Touch: 3.56 (.85) In(msec2); No Touch 3.65 (.74) In(msec2) | Touch SF: 3.37 (.56) In(msec2), d=.27; Reunion: 3.51 (.87) In(msec2), d=.06; No Touch SF: 2.74 (.68) In(msec2), d=1.29; Reunion: 2.83 (.75) In(msec2), d=1.11 | Condition Effect (SF and Reunion vs Baseline in both tasks); Did not test for Sex Effect | None reported |
| RSA | Fracasso et al 1994*^ | **44-58/ 5, 7, 10, and 13 months** | 5 months: 3.02 (.71) In(msec2); 7 months: 3.25 (.72) In(msec2); 10 months: 3.27 (.72) In(msec2); 13 months: 3.20 (.71) In(msec2) | 7 months: 2.97 (.86) In(msec2), d=.37; 10 months: 3.25 (.68) In(msec2), d=.03; 13 months: 3.13 (.75) In(msec2), d=.10 | Condition effect; No Age Effect; Did not test for Sex Effect | NA |
| RSA | Gray et al 2017^ | **167/ 4 months** | PP1 = 2.7 (.47) In(msec2 | SF = 2.53 (.55), d=.33; PP2 = 2.56 (.61) In(msec2), d=.26 | Did not test for Condition Effect; Sex Effect (Boys with prenatal stress ↑ RSA in PPI compared to girls) | NA |
| RSA | Ham et al 2006 | **12/ 5 months** | FF: Recovered: 3.3; Stable: 3.7; Dysregulated: 3.5; Protest: 3.5 [units unknow; pilot study] | SF Recovered: 3.5; Stable: 3.5; Dysregulated: 3.5; Protest: 3.0; Reunion: Recovered: 4.4; Stable: 4.2; Dysregulated: 3.9; Protest: 3.0, all ds=NA [units unknown] | Descriptive; Did not test for Condition or Sex effect | None reported |
| RSA | Hill-Soderlund et al 2008^ | **84/ 13 months** | 3.70 (1.03) In(msec2) | Episode 3: 3.71 (1.06) In(msec2), d=.01; Episode 4: 3.64 (1.10) In(msec2), d=.06; Episode 5: 3.53 (.90) In(msec2), d=.18; Episode 6: 3.39(1.16) In(msec2), d=.21; Episode 7: 3.5 (.98) In(msec2), d=.20; Episode 8: 3.66 (1.07) In(msec2), d=.04 | Condition Effect (Baseline versus Episodes 5, 6, and 7); No Effect of Sex | NA |
| RSA | Holochwost et al 2014^ | **95/ 6 months** | 3.72 (.91) In(msec2) | Normal: 3.57 (1.00) In(msec2), d=.16; SF: 3.46 (1.09) In(msec2), d=.26; Reunion: 3.51 (1.08) In(msec2), d=.31 | Did not test for Condition or Sex Effect | NA |
| RSA | Johnson et al 2014^ | **41/ 6 months** | 3.58 (1.76) In(msec2) | Start Arm Restraint: 3.42 (1.71) In(msec2), d=.09; End Arm Restraint: 2.91 (2.43) In(msec2), d=.32; Start SF: 3.42 (2.62) In(msec2), d=.07; End SF: 2.81 (2.31) In(msec2), d=.38 | Did not test for Condition Effect; No Effect of Sex | None reported |
| RSA | Moore 2009 | **48/ 6 months** | NA | NA, d=NA | Condition Effect (SF, reunion vs Play); Sex Effect (boys had ↑ RSA at baseline) | ↑ negative affect = ↓ ΔRSA in each SFP episode |
| RSA | Moore et al 2004*^ | **60/ 3 months** | 2.82 (.75) In(msec2) | Play: 2.84 (.76) In(msec2), d=.04; SF: 2.64(.71) In(msec2), d=.24; Reunion: 2.92 (.89) In(msec2), d=.12; Change in RSA Play: -.02 (.47), d=.03; SF: .18 (.46), d=.28; Reunion: -.11(.65), d=.14 | Condition Effect (Baseline vs SF; Play and SF; SF vs Reunion; RSA change SF vs Play and Reunion); No Sex Effect | None reported |
| RSA | Moore et al 2009^ | **89/ 6 months** | 3.68 (.85) In(msec2) | FF: 3.50 (.93) In(msec2), d=.20; SF: 3.38 (.96) In(msec2), d=.33; Reunion: 3.44 (.95) In(msec2), d=.27 | Condition Effect (Baseline vs SF); No Effect of Sex | ↑ positive and negative affect = ↑ RSA in FP; ↑ positive affect = ↑ RSA in Reunion; ↑ positive and negative affect = ↑ HP in reunion |
| RSA | Noten et al., 2019b^ | **116/ 6 months** | Still-face: 3.39 (.38) In(msec2); car seat: 3.30 (.40) In(msec2) | Still-face: 3.22 (.42) In(msec2), d=.44; Car seat: 3.30 (.55) In(msec2), d=0 | Condition Effect (SF vs Baseline); Did not test for Sex Effect | ↑ comfort seeking = ↑ RSA for still-face |
| RSA | Paret et al. 2015*^ | **48**/ 44 months | 7.37 (1.08) In(msec2) | 6.68 (1.11) In(msec2), d=.64 | Condition Effect (RSA ↓ in IbS); No Effect of Sex | No correlations |
| RSA | Perry et al 2012^ | **197/ 42 months** | 6.60 (1.12) In(msec2) | 5.34 (1.07) In(msec2), d=1.15; Suppression: 1.26 (.73) | Did not test for Condition Effect; No Effect of Sex | ↑ distraction = ↑ RSA suppression |
| RSA | Perry et al. 2016^ | **230/ 5 and 10 months** | 5 months: 3.88 (1.18) In(msec2); 10 months: 4.61 (1.10) In(msec2) | Withdrawal 5 months: -0.54 (1.45), d=.41; 10 months: -0.10 (1.40), d=.08 | Did not test for Condition or Age Effect; No Effect of Sex | ↑ maternal orienting = ↑ RSA withdrawal at 10 months |
| RSA | Pratt et al 2015^ | **122/ 5 months** | NA | Change: 0.4 (.73), d=.71; Reunion: 2.83 (1.01) In(msec2); all d=NA | Did not test for Condition Effect; No Effect of Sex | ↑ distress and ↓ disengagement = ↑ Change RSA |
| RSA | Provenzi et al 2015 | **65/ 4 months** | NA | NA, d=NA | No Effect of Condition or Sex. | None reported |
| RSA | Qu et al 2018^ | **206/ 14 months** | 3.65 (.99) In(msec2) | SF: 3.40 (1.09) In(msec2), d=.24; Reengage: 3.51 (1.31) In(msec2), d=.12 | Condition Effect (↓ RSA in SF and Reengagement); No Effect of Sex | ↑ negative affect = ↓ RSA in Reengage |
| RSA | Scrimgeour et al 2016 | **125/ 41 months** | NA | Change: .00 (1.0), d=NA | Did not test for Condition or Sex Effect | NA |
| RSA | Stone et al 2013 | **78/ 6 months** | If infant has bradycardia or not | Bradycardia: 3.22 (.75) In(msec2); Non-Bradycardia: 3.43 (.78) In(msec2), all ds=NA | Did not test for Condition Effect; No Effect of Sex | NA |
| RSA | Wagner et al 2018a^ | **97/ 24 months** | 4.86 (.98) In(msec2) | Suppression: .71 (.77), d=.88 | Did not test for Condition Effect; No Effect of Sex | NA |
| RSA | Wagner et al 2018b^ | **84/ 48 months** | 6.20 (1.18) In(msec2) | Anger: 6.34 (1.09) In(msec2), d=.12; Augmentation: −0.14 (range = 0.83 to −2.19) | Condition Effect; No Effect of Sex | NA |
| RSA | Weinberg et al 1996*^ | **45/ 6 months** | 3.165 In(msec2) | SF: 3.03 In(msec2); Reunion: 3.237 In(msec2), d=.36 | Condition Effect (↓RSA in SF); Did not test for Sex Effect | None reported |
| RSA | Zeytinoglu et al., 2019^ | **244/ 56 months** | 7.21 (1.11) In(msec2) | Locked box: 6.17 (1.14) In(msec2), d=.92; Toy removal: 6.84 (1.13) In(msec2), d=.33 | Did not test for Condition or Sex Effect | ↑ RSA = emotion regulation composite for toy removal only |

Abbreviations. HR = heart rate; HRV = heart rate variability; RSA = respiratory sinus arrhythmia; NA = not available Notes: (1) Studies included in meta-analysis are denoted by a symbol; * HR, ^ RSA, # HRV; (2) $ is used to denote studies that calculated HRV on raw data or used performed transformations on the data that did not allow collapsing into overall RSA meta-analysis.
